# Diversity for endoribonuclease nsp15-mediated regulation of alpha-coronavirus propagation and virulence

**DOI:** 10.1128/spectrum.02209-23

**Published:** 2023-11-08

**Authors:** Yunfei Xie, Chener Chen, Ding Zhang, Zhe Jiao, Yixi Chen, Gang Wang, Yubei Tan, Wanpo Zhang, Shaobo Xiao, Guiqing Peng, Yuejun Shi

**Affiliations:** 1 State Key Laboratory of Agricultural Microbiology, College of Veterinary Medicine, Huazhong Agricultural University, Wuhan, Hubei, China; 2 Key Laboratory of Preventive Veterinary Medicine in Hubei Province, The Cooperative Innovation Center for Sustainable Pig Production, Wuhan, Hubei, China; 3 Veterinary Pathology Laboratory, College of Veterinary Medicine, Huazhong Agricultural University, Wuhan, Hubei, China; Changchun Veterinary Research Institute, Changchun, China

**Keywords:** alpha-coronavirus, endoribonuclease, nsp15, propagation, virulence

## Abstract

Endoribonuclease non-structural protein 15 (nsp15) (EndoU) is conserved among coronaviruses (CoVs) and is crucial for viral replication, evasion of the innate immune system, and virulence. EndoU-deficient CoVs can activate the interferon (IFN) response and attenuate their virulence, and nsp15 is considered the target of attenuated vaccine development. Among alpha-CoVs, transmissible gastroenteritis virus (TGEV) and feline infectious peritonitis virus (FIPV) cause lethal diseases in piglets and cats, but the role of EndoU in viral propagation and virulence remains unclear. Here, we verified the TGEV and FIPV EndoU active sites His226 and His241 and found that the antagonization of SeV-induced IFN-β production by nsp15 depends on its EndoU activity. Furthermore, we constructed infectious clones of wild-type (WT) and EndoU-deficient (EnUmt) TGEV and FIPV. Unexpectedly, we found that both the WT and EnUmt viruses propagated efficiently in multiple types of immunocompetent (PK-15, IPI-2I, ST, CRFK, F81, and Fcwf-4) cells. Moreover, the results of infection experiments showed that compared with piglets and cats infected with the WT, the EnUmt virus-infected piglets and cats did not exhibit significantly reduced mortality, tissue injury, or viral shedding. Specially, the death of cats infected with EnUmt-FIPV occurred earlier than that of cats infected with WT. Hence, our results suggest that the function of EndoU is conserved, but nsp15-mediated regulation of the propagation and pathogenesis of CoVs are diverse. Our findings provide a reference for an in-depth understanding of EndoU-mediated immune escape and pathogenicity in CoVs.

**IMPORTANCE:**

Understanding the role of the endoribonuclease non-structural protein 15 (nsp15) (EndoU) in coronavirus (CoV) infection and pathogenesis is essential for vaccine target discovery. Whether the EndoU activity of CoV nsp15, as a virulence-related protein, has a diverse effect on viral virulence needs to be further explored. Here, we found that the transmissible gastroenteritis virus (TGEV) and feline infectious peritonitis virus (FIPV) nsp15 proteins antagonize SeV-induced interferon-β (IFN-β) production in human embryonic kidney 293 cells. Interestingly, compared with wild-type infection, infection with EnUmt-TGEV or EnUmt-FIPV did not change the IFN-β response or reduce viral propagation in immunocompetent cells. The results of animal experiments showed that EnUmt viruses did not reduce the clinical presentation and mortality caused by TGEV and FIPV. Our findings enrich the understanding of nsp15-mediated regulation of alpha-CoV propagation and virulence and reveal that the conserved functions of nonstructural proteins have diverse effects on the pathogenicity of CoVs.

## INTRODUCTION

Coronaviruses (CoVs) are a large group of viruses with widespread distribution that can cause diseases of the respiratory tract, digestive tract, and central nervous system of humans and animals and pose a serious threat to human health and economic development (1). CoVs, enveloped positive-sense RNA viruses, belong to the family *Coronaviridae* of the order *Nidovirales* and have four genera: *alpha-*, *beta-*, *gamma-* and *delta-CoVs* (2, 3). Among them, the majority of viruses are *alpha-* and *beta-CoVs*, which infect humans, livestock, and companion animals. Highly transmissible and pathogenic zoonotic CoVs include severe acute respiratory syndrome coronavirus (SARS-CoV), Middle East respiratory syndrome coronavirus (MERS-CoV), and the emerging SARS-CoV-2, which are *beta-CoVs* (4 – 6). Transmissible gastroenteritis virus (TGEV) and feline infectious peritonitis virus (FIPV), which share a close evolutionary relationship, are alpha-CoVs, which are widely circulated worldwide, causing major losses to the pork and pet industries (7).

CoVs have the longest known RNA genomes (approximately 30 kb), which encode 15–16 nonstructural proteins (nsp1 or nsp2 to nsp16), four structural proteins [spike (S), envelope (E), membrane (M), and nucleocapsid (N)], and a variety of genus-specific accessory proteins (8 – 12). Nonstructural protein 15 (nsp15) is an RNA uridylate-specific endoribonuclease (EndoU) that is conserved among CoVs and is considered an integral component of the coronaviral replicase-transcriptase complex (RTC); hexamerization is necessary for its function (13). However, EndoU activity may not be required for CoV RNA synthesis but is crucial for preventing the activation of host immune responses (14 – 16). In addition, EndoU-deficient mutant CoVs cause robust and early induction of type I interferon (IFN) and attenuated viral infection [porcine epidemic diarrhea virus (PEDV), mouse hepatitis virus (MHV) and infectious bronchitis virus (IBV)] in pigs, mice, and chickens (14, 17 – 20). Similarly, in HCoV-229E, EndoU activity is key to preventing early induction of dsRNA-mediated host cell responses. Replication of EnUmt-HCoV-229E is greatly attenuated and severely restricted in primary cells during the early phase of infection (20). Besides, EnUmt-MERS-CoV infection had little effect on the activation of innate immune pathways. EndoU and the accessory proteins 4a and 4b together suppress dsRNA-induced innate immunity during MERS-CoV infection (21). Hence, EndoU has been considered the key target for attenuating CoV infection.

TGEV causes severe diarrhea, vomiting, dehydration, and high mortality in piglets (22). Previous studies have shown that TGEV infection induces IFN-I production (23), unlike other CoVs, which inhibit IFN production (14, 16 – 19, 24). Feline infectious peritonitis (FIP), caused by a virulent feline CoV, is the leading infectious cause of death in cats (25, 26). FIPV is also divided into two serotypes: type I and type II (27). Since type I FIPV is difficult to isolate or rescue *in vitro*, in our previous research, the recombinant virus that replaced the S gene of the QS strain with the type II FIPV 79-1146 strain was rescued and designated as icQS-79. Administration of icQS-79 allowed successful establishment of an animal model of FIPV infection (26). Hence, we explored the effect of nsp15 function on the propagation and virulence of the QS79 strain. In addition, previous studies showed that FIPV strain DF2 infection could inhibit Sendai virus (SeV) or polyinosinic-polycytidylic acid [poly(I:C)]-induced IFN-β production (24).

In CoVs, nsp15 promotes virus replication by inhibiting the expression of host immune factors through EndoU activity. Among alpha-CoVs, TGEV infection significantly facilitates IFN-β production, but FIPV antagonizes SeV-induced IFN-β production. Here, we chose TGEV and FIPV as models to explore the effect of EndoU activity on virus propagation and virulence. We generated icTGEV and icFIPV with mutations in the catalytic histidine residues of EndoU and found that the EndoU-deficient viruses replicated as well as the wild-type (WT) virus, revealing that lack of EndoU activity does not affect viral propagation. Additionally, we evaluated the virulence of EnUmt-TGEV and EnUmt-FIPV in piglets and cats.

## RESULTS AND DISCUSSION

### TGEV and FIPV nsp15 antagonize SeV-induced IFN-β production in a manner dependent on their EndoU activity

**Fig 1:**
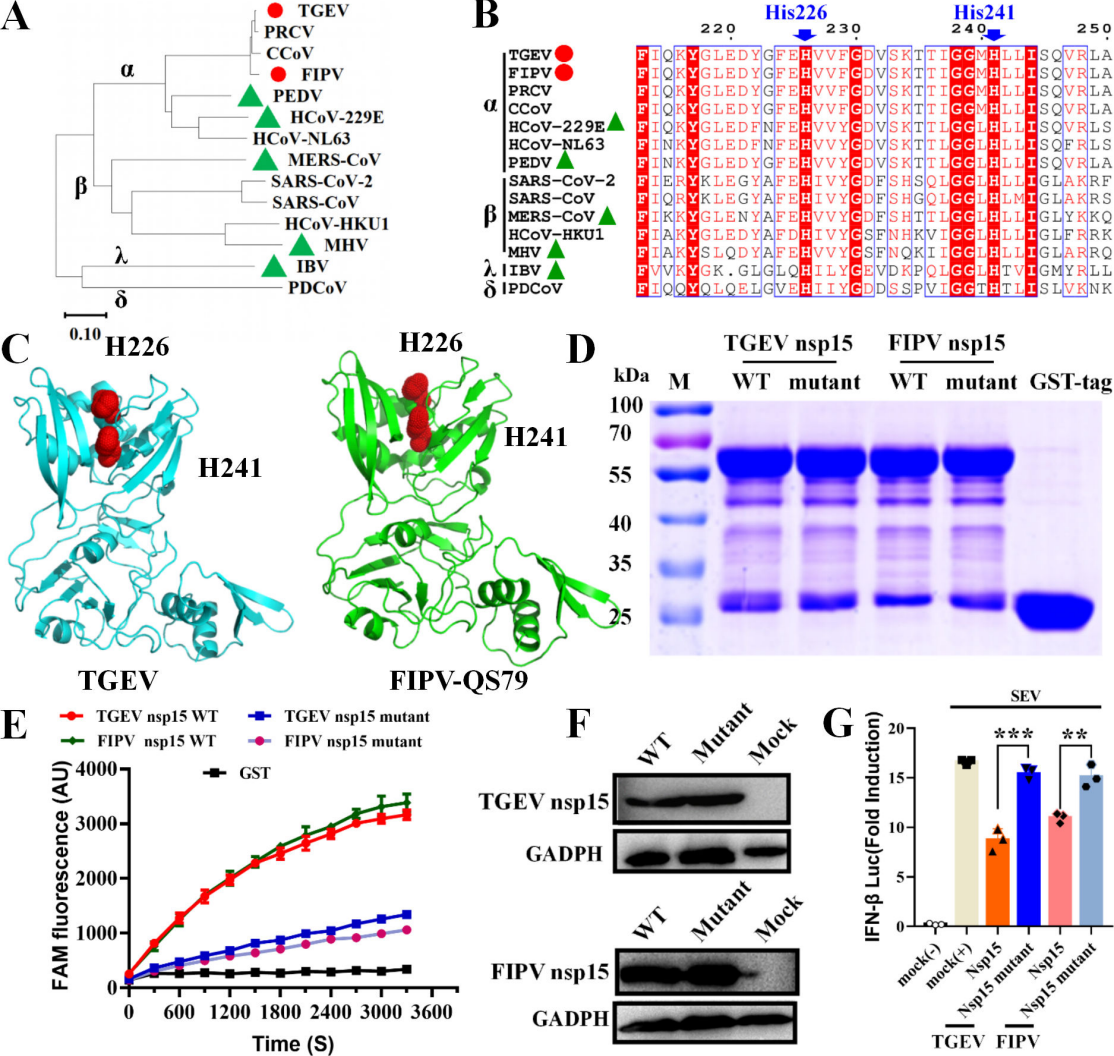
EndoU activity of TGEV and FIPV nsp15 antagonizes SeV-induced IFN-β production. (A) The phylogenetic relationships were analyzed based on the CoV nsp15 amino acid sequence. The red circle represents the virus strain described in this study; the green triangle indicates that the virus strain has been previously reported. (B) Sequence alignment of CoV (including *α-, β-, γ-, δ-CoVs*) nsp15 proteins. The residue numbers at the top refer to TGEV nsp15. The key residues for potential EndoU activity sites are marked with blue arrows. (C) Structures of TGEV and FIPV nsp15 proteins. Structural models were predicted using SWISS-MODE. EndoU activity sites (His226 and His241) are marked with red dots. (D) SDS–PAGE analysis of nsp15 and mutants. WT and mutant nsp15 proteins fused with a GST tag (approximately 64 kDa). The purified GST tag (approximately 26 kDa) was used as a mock. (E) Fluorescence resonance energy transfer (FRET)-based EndoU activity analysis. The enzymatic activity values of the nsp15 WT or mutants (H226A, H241A) are depicted with different colors. The values of the triplicate experimental results are shown. (F) Western blotting analysis of TGEV and FIPV nsp15 in human embryonic kidney 293 (HEK-293T) cells. (G) Loss of EndoU activity led to significant recovery of the IFN response in HEK-293T cells. The data shown represent the means ± standard deviations (SDs), and all experiments were repeated three times. The significant differences are indicated as follows: *P < 0.05, **P < 0.01, and ***P < 0.001.

nsp15 is a uridine-specific endoribonuclease that can antagonize the host immune system and promote viral replication (16). Evolutionary analysis showed that TGEV and FIPV belonged to the same evolutionary clade, but the evolutionary relationship between them and PEDV, HCoV-229E, MERS-CoV, MHV, and IBV is distant (Fig. 1A). Sequence alignment and structural analysis of CoV nsp15 proteins demonstrated that His226 and His241 were potential active site residues of TGEV and FIPV (Fig. 1B and C). To verify the EndoU activity of the WT and EnUmt proteins, we expressed and purified the recombinant protein in *Escherichia coli*. Because WT nsp15 expression was toxic to *E. coli* (17, 28), we expressed WT and mutant recombinant proteins fused with a glutathione S-transferase (GST) tag (nsp15 with GST tag, approximately 64 kDa; GST tag, 26 kDa) (Fig. 1D). Next, we performed enzyme activity assays for WT nsp15 and the H226A and H241A mutants of TGEV and FIPV under identical conditions, and the mutant activity was at least threefold less than that of the WT (Fig. 1E). Hence, the H226A and H241A mutations severely diminished EndoU activity, revealing that the two residues are crucial for catalytic activity. Furthermore, we verified the effect of TGEV and FIPV nsp15 overexpression on the IFN response. We found that the overexpression of nsp15 markedly inhibited the activity of the IFN-β luciferase reporter induced by SEV, while the mutants (H226A and H241A) lost the capacity to block the activation of the IFN-β promoter (Fig. 1F and G). The above results suggest that His226 and His241 are the key amino acid residues of nsp15 inhibiting IFN-β production.

### Construction of infectious clones of WT and EnUmt for TGEV and FIPV

**Fig 2:**
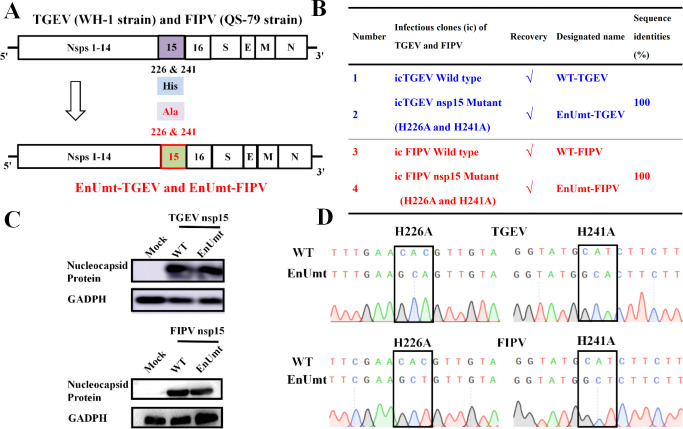
Recovery of infectious clones of the WT and EndoU mutants for TGEV and FIPV. (A) Schematic diagram of the TGEV and FIPV genomes and the location of mutations in the mutant viruses. (B) List of recombinant viruses rescued in this study. The sequence identities were aligned using ClustalW2. (C) Western blotting analysis of the nucleocapsid protein in the WT and EnUmt virus strains. Glyceraldehyde-3-phosphate dehydrogenase (GAPDH) was used as an internal reference. (D) Mutation stability analysis of EnUmt-TGEV and EnUmt-FIPV.

To evaluate the role of nsp15 during viral replication, we used our reverse-genetics system to construct infectious clones of TGEV and FIPV (QS-79) (26, 29). Single alanine substitutions at the catalytic H226 and H241 sites of nsp15 were constructed in the TGEV and FIPV strains, and the strains obtained were designated as EnUmt-TGEV and EnUmt-FIPV, respectively (Fig. 2A and B). Western blotting showed that the N protein was present in all the recombinant strains, which indicated that the recombinant virus was successfully rescued (Fig. 2C). To evaluate the stability of these EnUmt viruses, after five passages, the full-length gene sequence was analyzed by sequencing, and no other mutation sites were found except the H226A and H241A mutations, indicating that the EnUmt viruses had good genetic stability (Fig. 2B and D; Supplemental sequence information).

Meanwhile, we verified the effect of nsp15 on viral replication and found that the rescue of the icTGEV with deletion of the nsp15 gene was unsuccessful. Moreover, we performed deletion mutations in the N-terminal regions (15–19 aa, 15–24 aa, 20–24 aa, 20–28 aa, and 25–28 aa) that affected the formation of nsp15 hexamers. For each mutant, we used three independent clones for viral recovery and found that the corresponding icTGEV could not be rescued. These results suggest that the hexameric conformation of CoV nsp15 plays an important role in viral genome replication and may function in the recruitment of transcriptional replication complexes (13). For CoVs, the loss of EndoU activity does not significantly reduce virus replication, but nsp15 is still very important for normal virus replication, and the specific mechanism needs to be further explored.

### TGEV infection induces IFN-β production, and deficiency in EndoU activity does not reduce TGEV propagation *in vitro*

**Fig 3:**
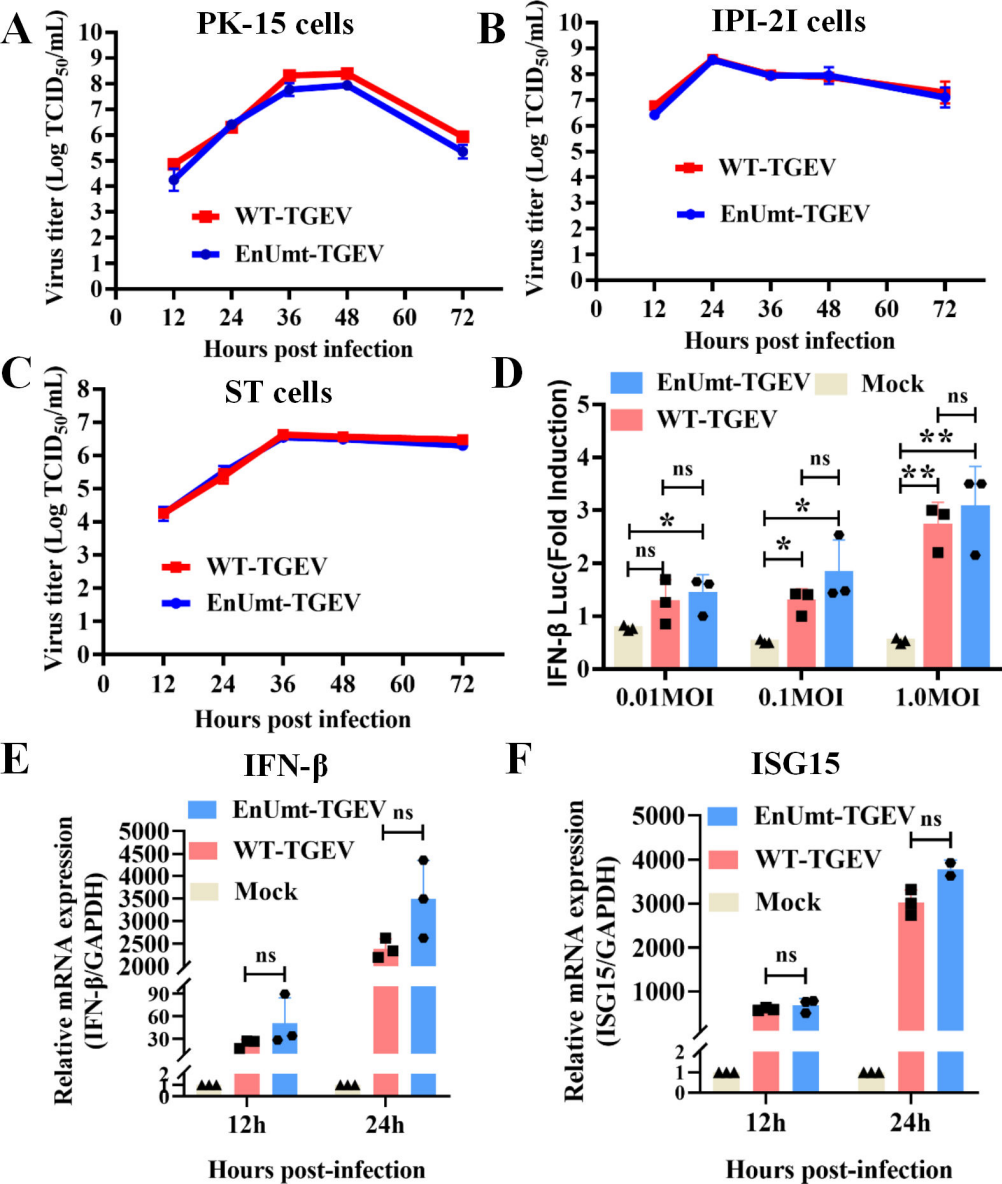
Deficiency in EndoU activity did not impair TGEV propagation. (A–C) Viral titer determination for WT- and EnUmt-TGEV in PK-15, IPI-2I, and ST cells. Cell culture supernatants [infection dose, multiplicity of infection (MOI) = 0.1] were collected at the indicated hours post-infection (hpi). The titer of infectious virus was determined using a 50% tissue culture infectious dose (TCID_50_) assay performed in triplicate, and the results are shown as the mean ± SD. Data sets at the same time point were analyzed with an unpaired *t*-test. (D) TGEV dose-dependently triggers activation of the IFN-β signaling pathway. PK-15 cells were cotransfected with pRL-TK and IFN-β-Luc, followed by infection with increasing doses of TGEV (MOI = 0.01, 0.1, and 1) at 12 h post-transfection. At 24 h after the initial transfection, the cells were infected with SeV. The cell lysates were collected for dual luciferase assays at 24 hpi. (E and F) WT- and EnUmt-TGEV infection induces a consistent type I IFN response. At 12 h and 24 h after TGEV infection (MOI = 0.1), cells were lysed to collect total RNA for cDNA synthesis, and quantitative PCR was used to measure the relative expression levels of the indicated mRNAs for IFN-β (E) and ISG15 (F). The representative data sets of three independent experiments are shown. Values are presented as the mean ± SD and were analyzed by unpaired *t*-test. The significant differences are indicated as follows: *P < 0.05 and **P < 0.01.

Previous studies demonstrated that the immune antagonism of nsp15 was cell-type dependent and that only immunocompetent cells could reveal the effect of the loss of EndoU activity (14, 17 – 19). To determine whether the mutation affected viral replication, we compared the replication kinetics of EnUmt-TGEV in porcine kidney (PK-15) cells, porcine small intestinal epithelial (IPI-2I) cells, and swine testicular (ST) cells over 72 h. Unexpectedly, we found that EnUmt-TGEV replicated efficiently in immunocompetent cells, with kinetics that were similar to those observed for WT-TGEV (Fig. 3A through C). These results are not consistent with those for PEDV, HCoV-229E, MHV, and IBV, and the replication ability of EnUmt viruses decreased significantly in immunocompetent cells (17 – 19, 30).

To explore the mechanism underlying the differences in the IFN response, we analyzed the effect of TGEV on IFN-β in PK-15 cells and performed dual luciferase assays. We found that TGEV infection significantly induced the activation of the IFN-β promoter in a dose-dependent manner, but there was no significant difference in IFN level induced by WT- and EnUmt-TGEV (Fig. 3D). Moreover, the mRNA expression levels of IFN-β and its downstream factor interferon-stimulated gene 15 (ISG15) were also upregulated in WT- and EnUmt-TGEV-infected cells (Fig. 3E and F); similarly, there was no significant difference between them. Previous research has shown that loss of EndoU activity leads to the accumulation of dsRNA, which stimulates a strong innate immune response, affects CoV replication and reduces virulence (14, 17 – 21). Hence, we measured the abundance of dsRNA in PK-15 cells and did not observe a significant difference between WT- and EnUmt-infected PK-15 cells (Fig. S1A). This may have led to no significant difference in IFN-β and ISG15 expression levels caused by EnUmt-TGEV infection. Moreover, previous data also suggest that MHV nsp15 may not affect the abundance of cytosolic dsRNA, but may affect the distribution of dsRNA in MHV-infected bone marrow-derived macrophages (BMDMs) (14). Therefore, how TGEV nsp15 affects the formation of dsRNA and how viral genome replication is regulated need to be further explored. Overall, compared with the WT, EnUmt-TGEV did not significantly activate IFN response, which could explain why there was no significant difference in their replication.

### FIPV infection antagonizes SeV-induced IFN-β production, and deficiency in EndoU activity does not inhibit viral propagation *in vitro*

**Fig 4:**
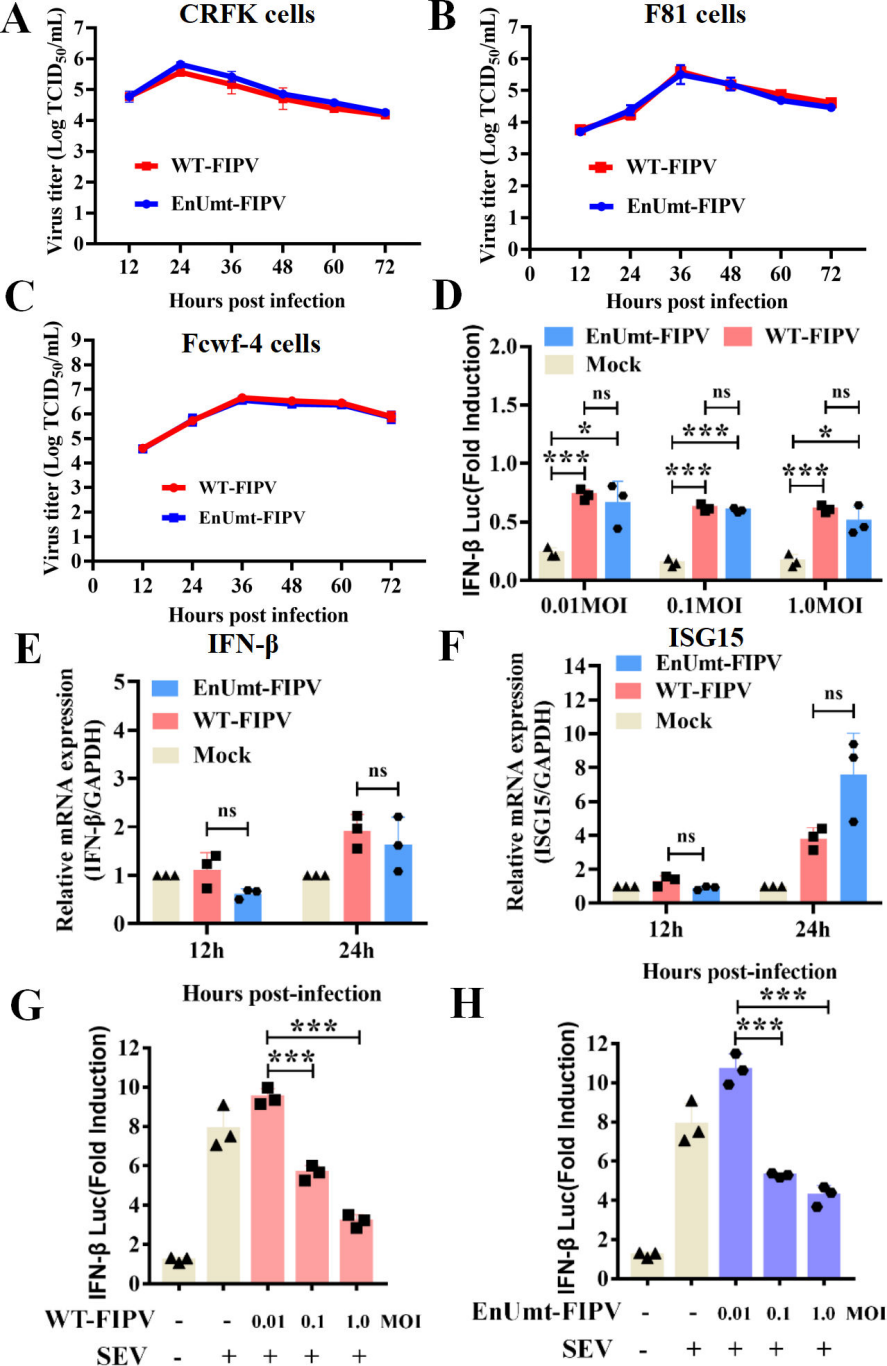
Deficiency in EndoU activity did not impair FIPV propagation. (A–C) Viral titer determination for WT- and EnUmt-FIPV in CRFK, F81, and Fcwf-4 cells. Cell culture supernatants (infection dose, MOI = 0.1) were collected at the indicated hours post-infection (hpi). The titer of infectious virus was determined using a TCID_50_ assay performed in triplicate, and the results are shown as the mean ± SD. Data sets at the same time point were analyzed with an unpaired *t*-test. (D–F) FIPV infection could not activate IFN production in CRFK cells. CRFK cells were cotransfected with pRL-TK and IFN-β-Luc, followed by infection with increasing doses of FIPV (MOI = 0.01, 0.1, and 1) at 12 h post-transfection. The cell lysates were collected for dual luciferase assays at 24 hpi (D). At 12 h and 24 h after FIPV infection (MOI = 0.1), cells were lysed to collect total RNA for cDNA synthesis, and quantitative PCR was used to measure the relative expression levels of the indicated mRNAs for IFN-β (E) and ISG15 (F). The representative data sets of three independent experiments are shown. Values are presented as the mean ± SD and were analyzed by an unpaired *t*-test. (G and H) FIPV dose-dependently inhibited SeV-induced IFN-β production. CRFK cells were cotransfected with pRL-TK and IFN-β-Luc, followed by infection with increasing doses of FIPV (MOI = 0.01, 0.1, and 1) at 12 h post-transfection. At 24 h after the initial transfection, the cells were infected with SeV. The cell lysates were collected for dual luciferase assays at 24 hpi. The representative data sets of three independent experiments are shown. The values are presented as the mean ± SD and were analyzed by an unpaired *t*-test. The significant differences are indicated as follows: *P < 0.05, **P < 0.01, and ***P < 0.001.

Among alpha-CoVs, TGEV and FIPV are in the same evolutionary branch and are closely related (Fig. 1A and B). However, unlike TGEV, FIPV infection could inhibit the IFN response in Crandell-Rees feline kidney (CRFK) cells (24). Hence, we explored the replication ability of WT- and EnUmt-FIPV in immunocompetent cells. Interestingly, we found that the results were consistent with those for TGEV. EnUmt-FIPV replicated efficiently in CRFK, F81, and Felis catus whole fetus (Fcwf)-4 cells, with kinetics that were similar to those observed with WT-FIPV (Fig. 4A through C). To further clarify the relationship between viral replication and the IFN response of FIPV-infected cells, the IFN-β promoter luciferase reporter system and qRT-PCR were used to analyze the production of IFN-β and its downstream factor ISG15. As shown in Fig. 4D through F, IFN-β promoter activity was barely detectable in WT- and EnUmt-infected cells, indicating that EnUmt-FIPV infection failed to activate IFN-β promoter activity. Furthermore, we found that SeV-induced activation of the IFN-β promoter was significantly inhibited by WT- and EnUmt-FIPV infection in a dose-dependent manner (Fig. 4G and H). Similarly, there was no significant difference in dsRNA accumulation in WT- and EnUmt-FIPV-infected CRFK cells (Fig. S1B). These results indicate that WT and EnUmt-FIPV infection could inhibit type I IFN signaling, which resulted in no change in the replication ability between WT- and EnUmt-FIPV.

### Deficiency in EndoU activity did not attenuate TGEV virulence in piglets

**Fig 5:**
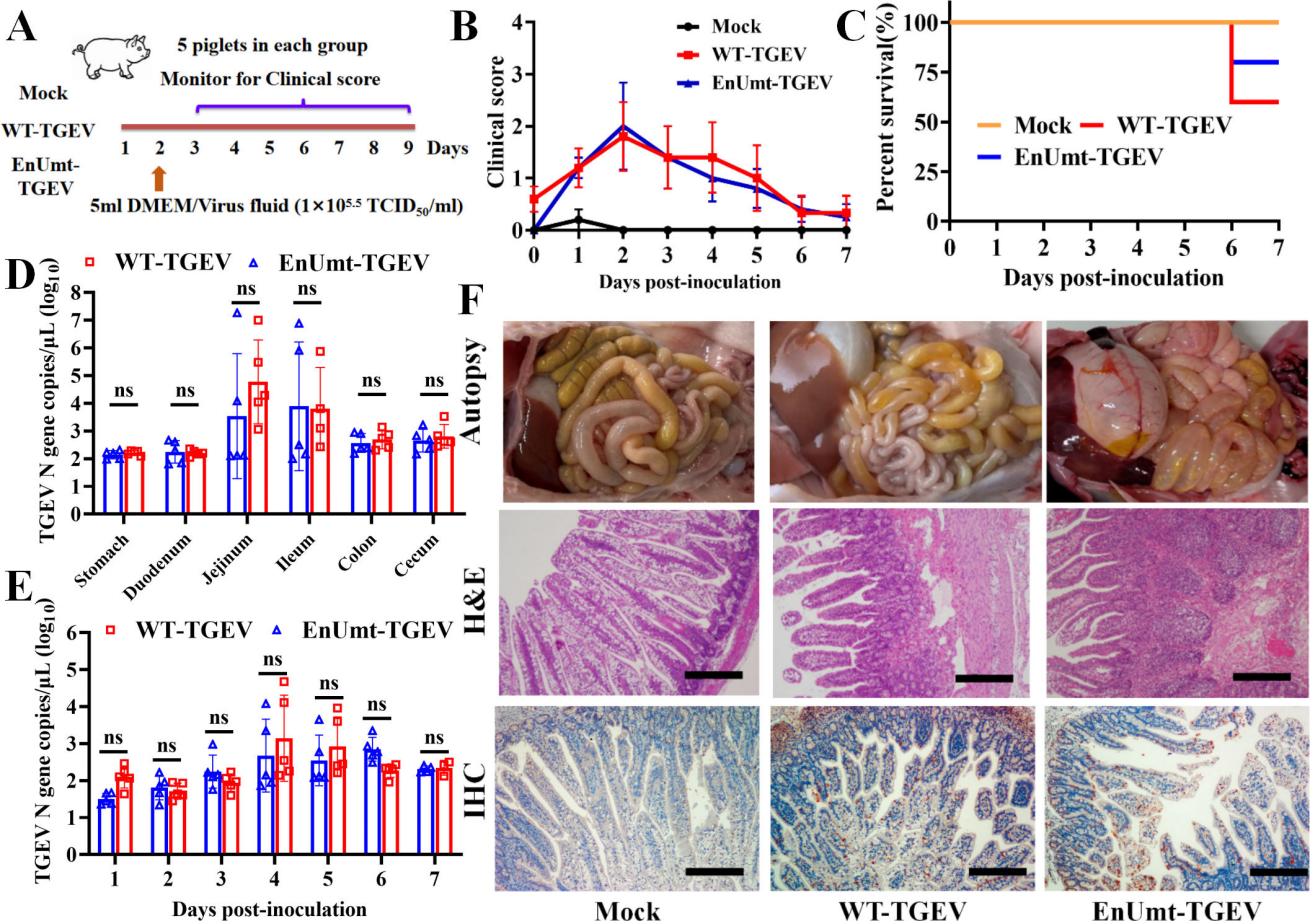
Evaluating the clinical presentation, mortality, and shedding of WT- and EnUmt-TGEV in piglets. (A) A total of 15 piglets were randomly grouped into three groups. These piglets were either mock or infected with WT- or EnUmt-TGEV. (B) Clinical symptoms were evaluated and scored daily. The score was based on the status of feces and the overall appearance of each piglet. Individual values and geometric means are presented. (C) Percent survival of the piglets in the different infected groups. (D) Statistical analysis of TGEV antigen distribution in the gastrointestinal tract of the piglets. RNA was isolated from different tissues (stomach, duodenum, jejunum, ileum, colon, and cecum) and subjected to quantitative PCR to determine the number of genomic RNA copies per microliter of the sample. Values were analyzed with unpaired *t*-tests used for comparisons between the groups. (E) Analysis of fecal shedding in piglets infected with WT- or EnUmt-TGEV. RNA was isolated from rectal swab samples and subjected to quantitative PCR to determine the number of genomic RNA copies per microliter of the sample. Values were analyzed with unpaired *t*-tests used for comparisons between the groups. (F) Histology and immunohistochemical staining of uninfected mock, WT-TGEV-infected, and EnUmt-TGEV-infected piglet ileum. Images show representative histological slides of ileum specimens showing hematoxylin and eosin (H&E) staining and immunohistochemistry (IHC) staining using rabbit anti-TGEV N protein antibody. Scale bar, 200 µm. The representative data sets (B–E) of five independent experiments are shown.

Then, we evaluated the clinical presentation, mortality, and shedding of EnUmt-TGEV in piglets. As shown in Fig. 5A, 15 piglets were randomly divided into three groups. The infection groups were orally inoculated with 5 mL (1 × 10^5.5^ TCID_50_/mL) of WT- and EnUmt-TGEV, and the mock group was orally inoculated with 5 mL of Dulbecco’s modified Eagle’s medium (DMEM). All the piglets in the WT- and EnUmt-TGEV groups exhibited clinical symptoms and weight loss. One of the five pigs in the EnUmt-TGEV infection group and two of the five pigs in the WT-TGEV infection group appeared dead at 6 days post-inoculation, whereas the piglets in the mock group remained healthy (Fig. 5B and C). The WT group had a mortality rate of 40% at day 6 post-challenge, while the EnUmt-TGEV infection group had a mortality rate of 20% (Fig. 5C). Meanwhile, we detected viral antigen distribution in different tissues (stomach, duodenum, jejunum ileum, colon, and cecum) and the fecal shedding of viral RNA and found that there was no significant difference between the WT- and EnUmt-TGEV infection groups (Fig. 5D and E).

Additionally, necropsy observation of the moribund piglets in the WT- and EnUmt-TGEV groups revealed that the small intestines were filled with watery contents. In particular, the intestinal walls in the ileum section of the intestines of these piglets were clearly thinner and more transparent compared with those of the mock group (Fig. 5F). Moreover, we carried out hematoxylin and eosin (H&E) staining analysis and found that compared with the mock group, the piglets infected with WT- and EnUmt-TGEV exhibited classic microscopic lesions within sections of the small intestine exhibiting villus atrophy with degeneration and necrosis of villus tip enterocytes. Besides, the viral antigen was detected by immunohistochemistry (IHC) within intestinal sections of the WT- and EnUmt-TGEV-infected piglets (Fig. 5F), which are consistent with the results of antigen detected by RT-PCR. Furthermore, we measured the levels of IFN-β and ISG15 in the tissues (stomach, duodenum, jejunum, ileum, colon, and cecum) of piglets infected with WT- and EnUmt-TGEV, and there was no significant difference among them (Fig. S2A and B). Overall, these results suggest that EnUmt-TGEV did not significantly alter enteric tropism or reduce virulence compared to the WT-infected piglets.

### Deficiency in EndoU activity did not attenuate FIPV virulence in cats

**Fig 6:**
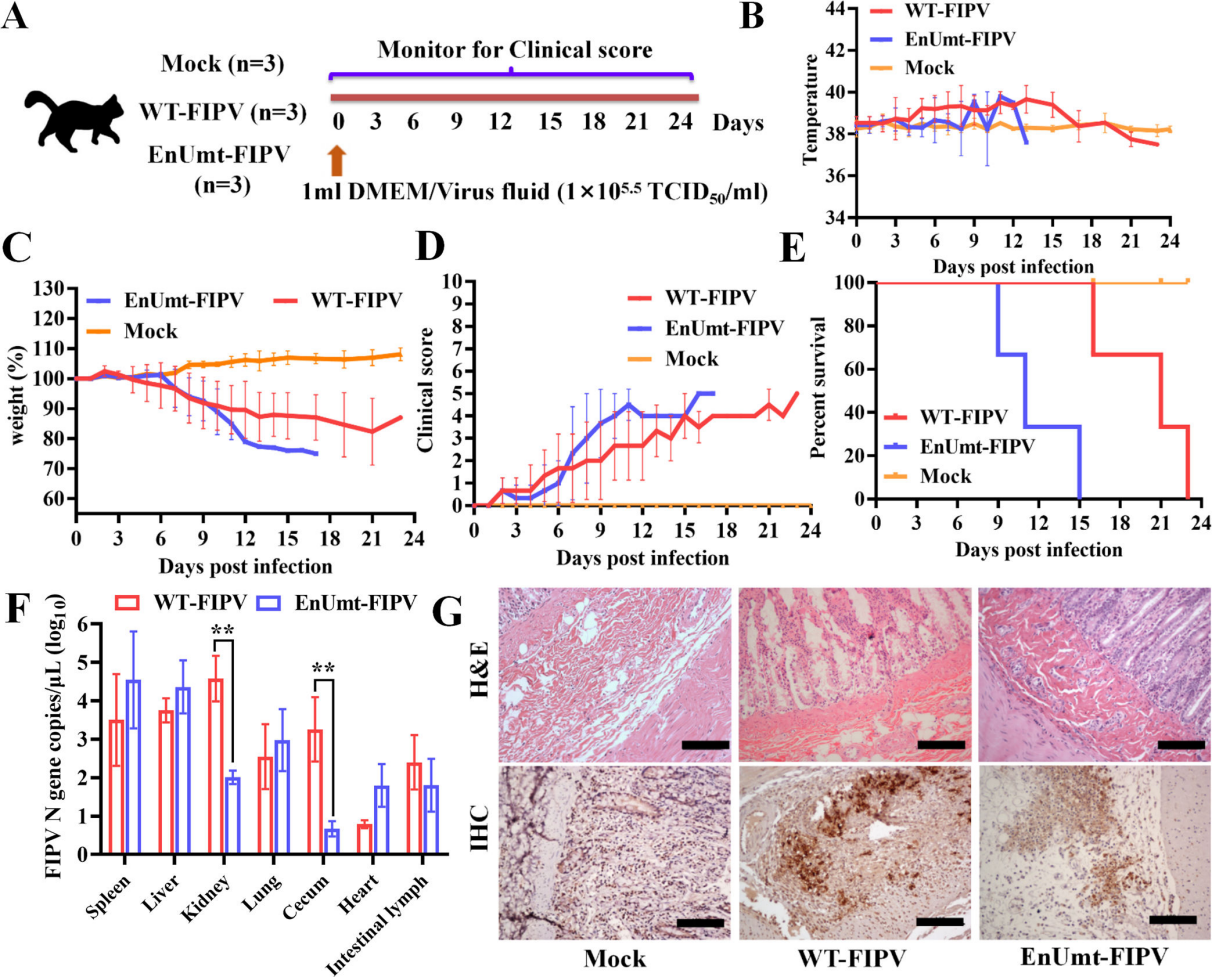
Evaluating the clinical presentation and mortality of WT- and EnUmt- FIPV in cats. (A) Schematic diagram of the animal experiment. A total of nine cats were randomly grouped into three groups. These cats were either mock or infected with WT- and EnUmt-FIPV. (B) Response of cat body temperatures to WT- and EnUmt-FIPV infection over time. (C) Results of weight monitoring after WT- and EnUmt-FIPV infection. On day 0 before infection, the body weight was 100%. (D) The clinical status scores of the cats (1 point was assigned in any of the following cases) were as follows: 1, cats that had a fever (>39°C); 2, cats that were anorexic; 3, cats that were lethargic; 4, cats that had weight loss (>5%); and 5, cats that were unable to stand, had severe dyspnea, or were moribund. (E) Percent survival of the cats in the different infection groups. (F) Statistical analysis of FIPV antigen distribution in different tissues of cats. RNA was isolated from different tissue (spleen, liver, kidney, lung, cecum, heart, and intestinal lymph) samples and subjected to quantitative PCR to determine the number of genomic RNA copies per microliter of sample. Values were analyzed with unpaired *t*-tests used for comparisons between the groups, **P  <  0.01. (G) Histology and immunohistochemical staining of uninfected mock, WT-FIPV-infected and EnUmt-FIPV-infected cat cecum. Images show representative histological slides of ileum specimens showing H&E staining and IHC staining using rabbit anti-FIPV N protein antibody. Scale bar, 200 µm. The representative data sets (B–F) of three independent experiments are shown.

To evaluate the pathogenicity of EnUmt-FIPV in cats, nine adult cats with good mental status and no maternal antibodies were selected and randomly divided into three groups. Three cats in the virus (WT- and EnUmt-FIPV) infection group were orally administered 1 mL of 10^5.5^ TCID_50_/mL virus solution, and three cats in the mock group were orally administered 1 mL of DMEM; the clinical symptoms of the cats in the three groups were continuously monitored (Fig. 6A). The cats in both the WT- and EnUmt-FIPV infection group developed symptoms such as fever (body temperature higher than 39°C), loss of appetite, weight loss (weight loss greater than 5%), and depression within a week of infection, and the mortality rate was 100%. The mock group had no obvious symptoms and no deaths (Fig. 6B through E). In addition, we found that the death of cats infected with EnUmt-FIPV occurred earlier than that of cats infected with WT (Fig. 6E). Moreover, we measured the viral content in the tissues by RT-PCR, and the WT-FIPV content in the kidney and cecum was higher than the EnUmt-FIPV content (Fig. 6F). The reason for the low viral load in the tissue of the EnUmt-FIPV infection group may have been its rapid death. The results of H&E staining and IHC also showed that viral infection caused tissue damage and neutrophil infiltration, and the virus was detected in the cecum (Fig. 6G). Furthermore, we measured the levels of feline serum amyloid A (fSAA) and total bilirubin (TBIL) in the serum of cats infected with WT- and EnUmt-FIPV and the expression levels of IFN and ISG15 in infected tissues (heart, liver, cecum, spleen, kidney, and intestinal lymph) (Fig. S2C and D, Fig. S3). Although there was no significant difference in the expression of IFN and ISG15 between WT- and EnUmt-FIPV-infected cats, the expression of fSAA and fTBIL increased significantly in cats infected with EnUmt-FIPV, indicating that inflammatory reaction and liver injury caused by EnUmt-FIPV infection were more serious. This may be one of the causes of rapid death in EnUmt-FIPV infection group, but the specific pathogenesis still needs to be further studied. The above results verified that the virulence of the EnUmt-FIPV was not attenuated.

### Diversity in nsp15-mediated regulation of coronavirus propagation and virulence

nsp15 is an EndoU that has conserved functions in CoVs and arteriviruses (31 – 33). The structures of EndoU have been determined, revealing their function as hexamers or dimers, and two histidine residues are key conserved catalytic residues (28, 33 – 36). Mutagenesis studies of the arterivirus EndoU revealed pleiotropic effects on replication; however, loss of EndoU activity is nonlethal to CoVs, only affecting their level of replication (17 – 19, 30). Previous studies have shown that the biological unit of CoV nsp15 is a hexamer, and the N-terminal domain (1–27 aa) is the major determinant of hexamerization (37). In the atomistic model of the SARS-CoV-2 replication-transcription complex, hexameric nsp15 forms a larger complex with nsp10/nsp14 and nsp10/nsp16, which is then capable of recruiting the polymerase complex. Hence, the N-terminal deletion mutant of EndoU cannot form a hexamer, which may affect the function of RTC and viral proliferation (13). During the rescue process of the mutant virus, we performed three parallel experiments. Indeed, N-terminal deletion (15–19 aa, 15–24 aa, 20–24 aa, 20–28 aa, 25–28 aa) in EndoU led to a nonviable phenotype in TGEV. Hence, we also confirmed this conclusion that TGEV with EndoU-deleted or truncated could not propagate effectively.

**Fig 7:**
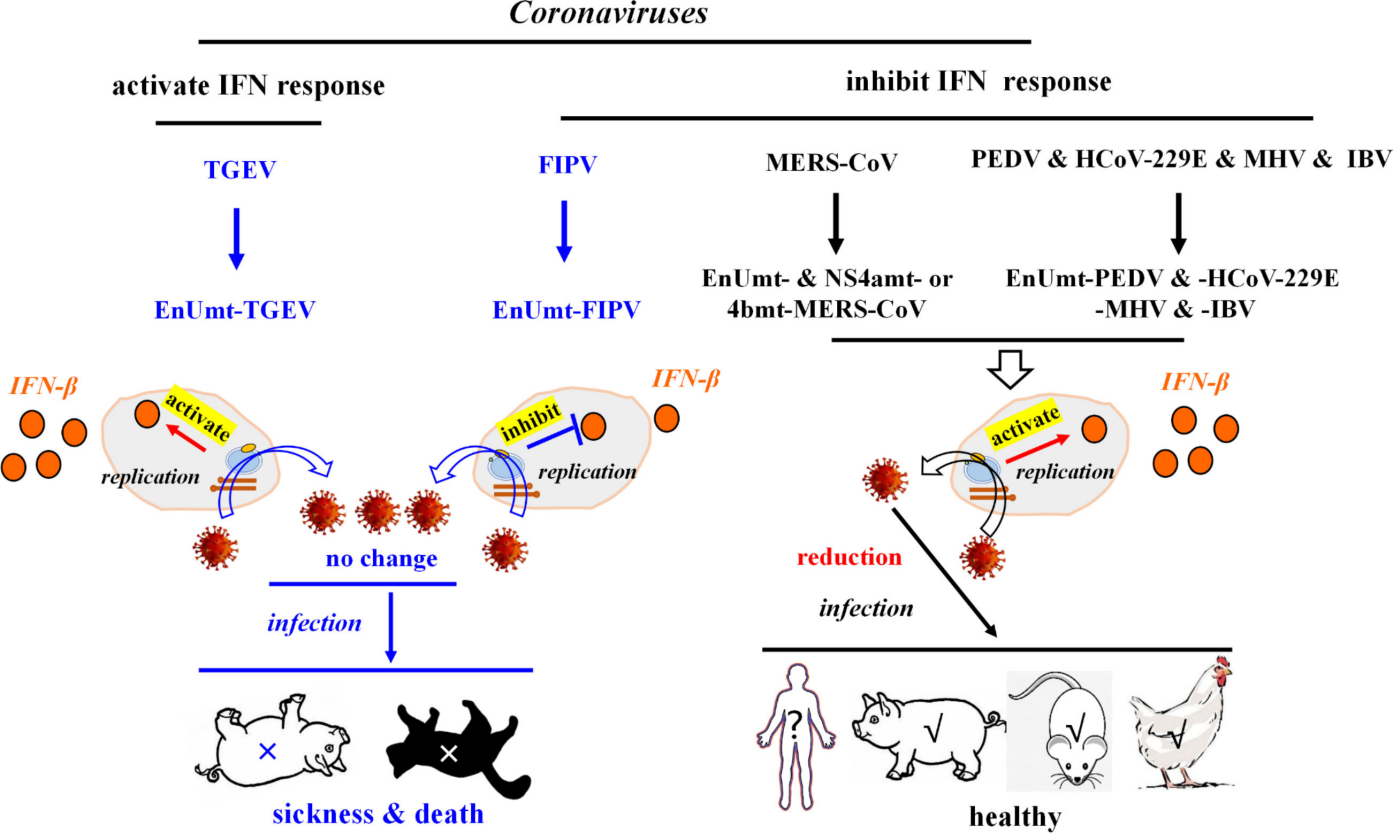
Diversity of nsp15-mediated regulation of coronavirus propagation and virulence. There are two patterns that affect the coronavirus virulence, one including TGEV, and the other including FIPV, MERS-CoV, PEDV, HCoV-229E, MHV, and IBV. Previous studies indicated that loss of EndoU activity in MERS-CoV [combined mutation of EndoU and the accessory protein 4a (NS4a) or 4b (NS4b)], PEDV, HCoV-229E, MHV, and IBV, IFN-β expression levels were significantly activated, thereby, reducing their propagation and virulence. However, we found that EnUmt-TGEV- and EnUmt-FIPV-regulated IFN production and propagation were basically consistent with those regulated by the WT strain. Moreover, the propagation ability and virulence of the EnUmt-TGEV and EnUmt-FIPV were not attenuated in piglets and cats.

Previous studies have reported that there were differences in the IFN response induced by CoV infection, especially TGEV infection, which could induce the transcriptional expression of IFN-I and increase extracellular IFN-β levels (38, 39). Although overexpression of TGEV nsp15 significantly inhibited activation of the IFN promoter in human embryonic kidney 293 (HEK-293T) cells (Fig. 1G), TGEV infection activated the IFN response in PK-15 cells. This indicates that nsp15 cannot play a strong role in IFN inhibition during TGEV replication; thus, EnUmt-TGEV could not significantly activate the IFN response to inhibit viral replication (Fig. 3A through C, Fig. 7). In addition, FIPV infection inhibited the IFN response, and the replication ability of EnUmt-FIPV was consistent with that of WT (Fig. 4A through C, Fig. 7), which was also surprising. In FIPV, a related study showed that nsp5 inhibited IFN-β production by cleaving nuclear factor κB (NF-κB) essential modulator (NEMO) (24). Our results showed that EnUmt-FIPV can inhibit the production of IFN-β (Fig. 4H). The change in IFN level caused by EnUmt-TGEV and EnUmt-FIPV infection was not significant, which may have been due to the difference in accessory proteins or differences in the activities of some of the other nsps.

Previous studies have shown that there is diversity in the number and function of accessory proteins encoded by CoVs (10, 11, 21). Indeed, TGEV contains three accessory genes: 3a, 3b, and 7 (12). Infection with icTGEV − Δ7 (lacking protein 7) led to increased proinflammatory responses and acute tissue damage *in vitro* and *in vivo* (12). FIPV contains five accessory proteins: 3a, 3b, 3c, 7a, and 7b (40, 41). FIPV protein 7a is a type I IFN antagonist that needs the presence of ORF3-encoded proteins to exert its antagonistic function (40, 41). Furthermore, only nsp15 in combination with the accessory protein 4a or 4b can significantly suppress dsRNA-induced innate immunity during MERS-CoV infection (21). Additionally, the function of CoV nsp15 is conserved, and His226 and His241 are the key active sites. Mutation of these sites led to a decrease in EndoU activity and the ability to inhibit IFN. Compared with that in other CoVs, the EndoU activity of TGEV and FIPV nsp15 mutant proteins was severely diminished but not completely lost (Fig. 1E). Therefore, the differences in the number and function of accessory proteins encoded by TGEV and FIPV as well as the activity of EnUmt together led to differences in proliferation and virulence between EnUmt-TGEV, EnUmt-FIPV, and other CoVs.

In this study, we found that the replication of EnUmt-TGEV and EnUmt-FIPV did not decrease in immunocompetent cells (Fig. 3A through C, Fig. 4A through C), resulting in clinical symptoms and death consistent with that observed with the WT in piglets or cats (Fig. 5C, Fig. 6E, Fig. 7). Our results are significantly different from those for other CoVs (PEDV, HCoV-229E, MHV, and IBV) and similar to those for MERS-CoV. Moreover, the pathogenicity of these EnUmt-CoVs (PEDV, MHV, and IBV) in piglets, mice, and chickens was significantly reduced (14, 17 – 21) (Fig. 7). Hence, we conclude that the effects of EndoU activity on the replication and pathogenicity of CoVs are diverse, and the development of attenuated vaccines in the future requires a comprehensive understanding of the role of nsps in CoV virulence (Fig. 7).

## MATERIALS AND METHODS

### Cells and viruses

The TGEV strain WH-1 (GenBank accession number HQ462571) and FIPV strain QS-79 are stored in our laboratory (26). Porcine kidney (PK-15) cells, porcine small intestinal epithelial (IPI-2I) cells, ST cells, CRFK cells, feline kidney (F81) cells, and HEK-293T cells were propagated at 37°C in a 5% CO_2_ incubator in DMEM (Gibco, Waltham, MA, USA) supplemented with 10% fetal bovine serum (Gibco, Waltham, MA, USA). Besides, the Fcwf-4 cells were propagated at 37°C in a 5% CO_2_ incubator in Eagle’s minimum essential medium (Gibco, Waltham, MA, USA) supplemented with 15% fetal bovine serum. The cells used in this experiment were purchased from the American Type Culture Collection (ATCC).

### Plasmid construction, protein expression, and purification

The alanine-substituted TGEV or FIPV nsp15 mutants (H226A and H241A) were cloned into the pGEX-6p-1 vector by homologous recombination with a GST tag. All constructs were validated by DNA sequencing. The recombinant plasmids described above were transformed into the *E. coli* strain BL21 (DE3) (Beijing TransGen Biotech, Co., Ltd.). The cells transformed with plasmids were cultured at 37°C in Luria-Bertani (LB) medium containing 50 µg/mL ampicillin. When the optical density (OD_600_) value of the culture reached 0.6–0.8, expression was induced with 0.5 mM isopropyl β-d-1-thiogalactopyranoside, and the cells were cultured at 37°C for an additional 4 h. Protein expression and purification were conducted according to our previously reported procedure (28, 42, 43). The proteins were eluted and diluted with buffer (20 mM Tris-HCl and 200 mM NaCl, pH 7.4). The concentration of purified nsp15 protein was determined by measuring the absorbance at 280 nm (A280) using a NanoDrop 2000c UV‒Vis spectrophotometer (Thermo Fisher Scientific).

### Enzymatic activity assay

According to a previously reported procedure (44), the EndoU activity assay was performed as follows: TGEV or FIPV nsp15 (WT, H226A, and H241A) protein, or GST-tagged protein (as a mock), each at a concentration of 2 µM was mixed with 1 µM RNA substrate (5′,6-carboxyfluorescein-dA-rU-dA-dA-6-carboxy-N,N,N′,N′-tetramethylrhodamine-3′, purchased from Sangon Biotech) in the reaction buffer (50 mM KCl, 50 mM HEPES, pH 7.5) with 5 mM MnCl_2_ and 1 mM DL-dithiothreitol (DTT) dissolved in water with 0.1% diethyl pyrocarbonate and incubated at 25°C for 60 min, measuring the absorbance at wavelengths ranging from 492 to 518 nm every 5 min. The values obtained in three independent measurements are shown.

### Luc reporter gene assay

According to a previously reported procedure (28), HEK-293T, PK-15, or CRFK cells were seeded into 48-well plates and incubated until the cells reached approximately 80% confluence. Then, the cells were cotransfected with 0.1 µg of the reporter plasmid [beta interferon-luciferase (IFN-β-Luc)], 0.01 µg of plasmid pRL-TK (Promega) encoding Renilla luciferase, 0.4 µg of wild-type plasmid, or 0.4 µg of mutant (H226A and H241A) plasmids using jetPRIME transfection reagent. The total transfected DNA amount was normalized to 0.51 µg by the addition of empty pCAGGS vector. At 24 h after the initial transfection, the cells were infected with SeV. Cell extracts were collected at the indicated time points, and luciferase activity was measured with a dual-specific luciferase assay kit (Promega). All reporter assays were independently repeated three times.

### Construction and recovery of recombinant viruses

Recombinant viruses were constructed according to our previously reported procedure (26, 29). The design of the relevant primers is shown in Table 1. Each corresponding mutant virus was constructed by CRISPR‒Cas9 technology. Briefly, bacterial artificial chromosome (BAC) plasmid (pBAC)-TGEV and pBAC-FIPV were digested in a 50 mL reaction mixture consisting of 5 mg of pBAC-TGEV and pBAC-FIPV, 5 mL of Cas9 (New England Biolabs), 10 mg of sgRNA, and 5 mL of nuclease reaction buffer by incubation at 37°C overnight. For virus recovery, HEK-293T cells seeded in six-well plates were transfected with 2 µg of purified BAC DNA and 4 µL of the jetPRIME transfection reagent (Vazyme) according to the manufacturer’s instructions. Transfected cultures were added to PK-15 or CRFK cells at 24–48 h after transfection. Virus recovery was monitored by cytopathic effect (CPE), and the newly reconstituted viruses were then plaque-purified and propagated in PK-15 or CRFK cells.

**TABLE 1:** Primers used to produce the specific sgRNA for pBAC-TGEV and pBAC-FIPV digestion

Primer	Sequence
sgRNA	AAAAGCACCGACTCGGTGCCACTTTTTCAAGTTGATAAC GGACTAGCCTTATTTTAACTTGCTATTTCTAGCTCTAAAAC
sgTGEV nsp15-F	GATCACTAATACGACTCACTATAGGGATGTGTTATTACTGAAAATTTTAGAGCTAGAAA
sgTGEV nsp15-R	GATCACTAATACGACTCACTATAGGGTGCATCTGGTGTTGCTCCTTTTAGAGCTAGAAA
sgFIPV nsp15-F	GATCACTAATACGACTCACTATAGACACTTATATGTTGTGGACAG TTTTAGAGCTAGAAA
sgFIPV nsp15-R	GATCACTAATACGACTCACTATAGGTGCTAATGGTGTTGCTCCG TTTTAGAGCTAGAAA

### Viral growth curves and western blotting

PK-15, IPI-2I, ST, CRFK, F81, and Fcwf-4 cells were infected with the recombinant virus (WT- and EnUmt-TGEV or WT- and EnUmt-FIPV) at a multiplicity of infection of 0.01 in six-well plates for 1 h and then washed three times with phosphate buffered saline (PBS). Subsequently, the supernatants of the infected cells at 12, 24, 36, 48, 60, and 72 h post-infection were collected and stored at −80°C. The viral titers at each time point were determined by the TCID_50_ method. The TCID_50_ assays were independently repeated three times. In addition, for western blotting, TGEV- and FIPV-infected cell samples were separated by 10% SDS–PAGE and transferred to polyvinylidene difluoride (PVDF) membranes (Bio-Rad). Each PVDF membrane was blocked with 5% (wt/vol) skim milk in Tris-buffered saline with Tween 20 (TBST) and then incubated overnight with the anti-TGEV and anti-FIPV N protein PAb (1:1,000) and an anti-GAPDH monoclonal antibody (1:5,000; Proteintech) at 4°C. Then, the membranes were incubated with goat anti-mouse secondary antibody (Boster Biological Technology Co., Ltd.) for 1 h (1:5,000). Finally, the membranes were visualized using an enhanced chemiluminescence system (Amersham Imager 600, GE Healthcare).

### Analysis of gene expression using RT-qPCR

Total RNA was extracted using TRIzol reagent (Invitrogen). Real-time RT-qPCR was performed using SYBR Green Real-Time PCR Master Mix (Toyobo Biologics, Osaka, Japan) and the ABI PRISM 7000 sequence detection system (Applied Biosystems). Individual transcripts in each sample were assayed three times. The fold change in gene expression relative to the control was calculated using the delta-delta cycles to threshold (ΔΔCT) method. Primers (Table 2) were designed using Primer Express software (version 3.0; Applied Biosystems, Carlsbad, CA, USA). The values obtained in the three independent measurements are shown.

**TABLE 2:** RT-qPCR primers

Species	Primer	Sequence
Porcine	IFN-β-F	GCTAACAAGTGCATCCTCCAAA
	IFN-β-R	AGCACATCATAGCTCATGGAAAGA
	ISG15-F	CCTGTTGATGGTGCAAAGCT
	ISG15-R	TGCACATAGGCTTGAGGTCA
	GAPDH-F	ACATGGCCTCCAAGGAGTAAGA
	GAPDH-R	GATCGAGTTGGGGCTGTGACT
TGEV	Nucleocapsid Protein-F	ACGCTTGGTAGTCGTGGTG
	Nucleocapsid Protein-R	GAATTGTTGCCTGCCTCT
Feline	IFN-β-F	GAAGGAGGAAGCCATATTGGT
	IFN-β-R	CTCCATGATTTCCTCCAGGAT
	ISG15-F	TCCTGGTGAGGAACCACAAGGG
	ISG15-R	TTCAGCCAGAACAGGTCGTC
	18s-F	CGGCTACCACATCCAAGGAA
	18s-R	GCTGGAATTACCGCGGCT
FIPV	Nucleocapsid Protein-F	TCGTGGTGACTCTAA
	Nucleocapsid Protein-R	GATGGAACGCATTCA

### Virulence of EnUmt-TGEV in piglets

Fifteen 2-day-old piglets from a TGEV-free sow were randomly divided into three groups and fed fresh liquid milk every 4 h. All piglets were confirmed to be free of TGEV, PEDV, porcine delta coronavirus, and rotavirus via an RT-PCR assay of piglet feces before viral challenge. The infection groups were orally inoculated with 5 mL (1 × 10^5.5^ TCID_50_/mL) of WT- and EnUmt-TGEV, and the mock group was orally inoculated with 5 mL of DMEM.

After infection, the clinical status was monitored every day. Any piglets exhibiting moribund signs or surviving piglets at 7 days post-infection were euthanized. Piglets were rectally swabbed and given a clinical diarrhea score at 0–7 days post-infection (dpi). Clinical diarrhea scores were assigned by the following criteria: 0 = normal feces, 1 = soft stool, 2 = semiliquid stool, 3 = liquid feces, and 4 = voluminous watery diarrhea. At necropsy, viral antigen distribution in different tissues (stomach, duodenum, jejunum, ileum, colon, and cecum) and fecal shedding were detected by RT-qPCR. The primers are shown in Table 2. The representative data sets of five independent experiments are shown.

### Virulence of EnUmt-FIPV in cats

According to our previous research (26), cats (from Jiaxiang Huarong Cat Farm, Jining, China) aged 1–2 years were used. Two months of feeding was performed to acclimatize the cats before FIPV infection. Nine cats were randomly divided into three groups, with three cats in the WT and EnUmt-FIPV infection group and three cats in the mock group. The three groups of cats were kept separately in different catteries in three strictly separated rooms. All FIPV-infected group cats were orally inoculated with 1 mL of viral liquid (WT- or EnUmt-FIPV) at 1 × 10^5.5^ TCID_50_/mL. All cats were monitored for body temperature, body weight, clinical state, and percent survival (including euthanasia) daily. When the last cat in the WT-FIPV-infected group died from FIP, cats in the mock group were euthanized. At necropsy, viral antigen distributions in different tissues (spleen, liver, kidney, lung, cecum, heart, and intestinal lymph) were determined by RT-qPCR. The primers are shown in Table 2. The representative data sets of three independent experiments are shown.

### Histopathological observation and IHC

Fresh tissues were fixed in 10% formalin solution at room temperature for 48 h, embedded in conventional paraffin, cut into 4 µm thin slices and stained with HE. Finally, the histological and pathological changes were observed and recorded under a light microscope. In addition, the slides were sealed with 10% normal goat serum (lot no. 15K06A09, Boster, China) at 37°C for 1 h and then incubated with rabbit anti-TGEV or anti-FIPV N protein polyclonal antibodies (1:500, prepared in our laboratory) in a wet box at 4°C overnight. The sections were then incubated with horseradish peroxidase (HRP) goat anti-mouse or anti-rabbit IgG polymer (clone no. GK600711A, Gene tech, China) in a wet box at room temperature for 1 h. Those slices were scanned by an Aperio CS2 (Leica, Germany). The positivity rate of IHC was analyzed by Leica imagescope ×64 software.

### Statistical analysis

Statistical analysis was carried out using GraphPad Prism 7.0. Statistical significance was determined using an unpaired two-tailed Student’s *t*-test. Data are presented as the mean values ± standard deviations (95% CI). *P  <  0.05 statistically significant; **P  <  0.01 highly significant; and ***P  <  0.001 and ****P  <  0.0001 extremely significant.

## SUPPLEMENTAL MATERIAL

Supplementary information spectrum.02209-23-s0001.docx Supplementary tables and figures.

ASM does not own the copyrights to Supplemental Material that may be linked to, or accessed through, an article. The authors have granted ASM a non-exclusive, world-wide license to publish the Supplemental Material files. Please contact the corresponding author directly for reuse.
