# Supplementary material for: Diversity for endoribonuclease nsp15-mediated regulation of alpha-coronavirus propagation and virulence
Source: Microbiol Spectr. 2023 Nov 8;11(6):e02209-23. doi: 10.1128/spectrum.02209-23 (PMC10715224; doi:10.1128/spectrum.02209-23)
Supplement: Supplementary information — Supplementary tables and figures. [file spectrum.02209-23-s0001.docx]

**Supplementary Information for:**

**Diversity for endoribonuclease nsp15-mediated regulation of alpha-coronavirus propagation and virulence**

Yunfei Xie^a,b,^*, Chener Chen^a,b,^*, Ding Zhang^a,b^, Zhe Jiao^a,b^, Yixi Chen^a,b^, Gang Wang^a,b^, Yubei Tan^a,b^, Wanpo Zhang^c^, Shaobo Xiao^a,b^, Guiqing Peng^a,b #^, Yuejun Shi ^a,b #^

a State Key Laboratory of Agricultural Microbiology, College of Veterinary Medicine, Huazhong Agricultural University;

b Key Laboratory of Preventive Veterinary Medicine in Hubei Province, The Cooperative Innovation Center for Sustainable Pig Production;

c Veterinary Pathology Laboratory, College of Veterinary Medicine, Huazhong Agricultural University;

* These authors contributed equally to this work. Author order was determined both alphabetically and in order of increasing seniority.

# To whom correspondence should be addressed:

Guiqing Peng: State Key Laboratory of Agricultural Microbiology, College of Veterinary Medicine, Huazhong Agricultural University, 1 Shi-zi-shan Street, Wuhan, 430070, China; penggq@mail.hzau.edu.cn; Tel. +86 15717136125; Fax. +86 27 87285816;

Yuejun Shi: College of Veterinary Medicine, Huazhong Agricultural University, 1 Shi-zi-shan Street, Wuhan, 430070, China; shiyuejun2017@mail.hzau.edu.cn; Tel. +86 18771048173; Fax. +86 27 87285816.

1. **Supplemental materals and methods**

**Genome sequencing**

To evaluate the stability of EnUmt-TGEV and EnUmt-FIPV, after 5 passages, the RNAs were extracted with Trizol reagent (Invitrogen) and cDNA for PCR was synthesized using HiScript II qRT SuperMix according to the manufacturer’s instructions. The full-length genomic sequences of EnUmt-TGEV and EnUmt-FIPV were amplified by PCR using corresponding primers (**Supplementary Table1**) and were validated by DNA sequencing. The sequence identities were aligned using ClustalW2 (1).

**Immunofluorescence assay**

According to the method previously reported (2), PK-15 cells and CRFK cells were infected with TGEV (WT and EnUmt, MOI=0.01) and FIPV (WT and EnUmt, MOI=0.01), respectively. The cells were fixed with 4% paraformaldehyde at 24 hpi. The permeabilized cells were blocked for 30 minutes with PBS containing 5% bovine serum albumin. Then, the cells were incubated with anti-dsRNA antibody (SCICONS, no. 10010200, 1:1,000) at 37°C for 2 hours. The secondary antibodies were Alexa Fluor 594 goat anti-mouse IgG (H + L) (Invitrogen, no. A-11005, 1:1,000) and Alexa Fluor 594 anti-rabbit IgG (H + L) (Invitrogen, no. A-11012, 1:1,000), and the cells were incubated at 37°C for 1 h. Finally, the cell nuclei were counterstained with 4’,6-diamidino-2-phenylindole (DAPI) (Sigma, no. D9542). Cell samples were observed and imaged with a fluorescence microscope (Thermo Fisher Scientific EVOS FL Auto). The fluorescence images were collected from three independent experiments, and 15 visual fields were collected from each image for statistical analysis.

**Determination of serum indexes in cats**

Nine cats were randomly divided into 3 groups, with 3 cats in the WT and EnUmt-FIPV infection group and 3 cats in the mock group. According to the manufacturer’s instructions, feline blood samples were centrifuged at 4500 rpm for 10 min, 10 μl of supernatant was taken after centrifugation and mixed with diluent, and 100 μl of the diluted serum was added to the reagent strip (no. V4011510BA) for serum amyloid A (SAA) detection. The immunofluorescence detector for pet (type no. YG-101) was used to perform automatic detection. Moreover, serum liver injury indexes were determined using the total bilirubin (TBIL) content via automatic biochemical analyzer (MNCHIP no. Celercare V5).

1. **Supplemental Table1 Genome Sequencing Primers**

| **Species** | **Primers** | **Sequence** |
| --- | --- | --- |
| TGEV | 1F | GGTTCCGTCCCTATTTCG |
|  | 1R | CCATCATATTCAAATGATGAACC |
|  | 2 F | GTGAATATTATTTCATGATGTCTAGTCC |
|  | 2 R | GTTAACTTGCATGCAGTCAC |
|  | 3 F | AGATAGTACTTGCAGCTAAGTG |
|  | 3 R | TAGGAGATGCTGCAACTTTG |
|  | 4 F | TGATGCAGAAAAGGCTTACC |
|  | 4 R | GTAAACAGAGCACAAACCAC |
|  | 5 F | GATCTTCCTTATGAAAGATTCACTG |
|  | 5 R | CTAACACATAACCTACTGATCC |
|  | 6 F | CTTGTACTTAAAGTCAACCAGG |
|  | 6 R | CGCTCAAAATCACTCTTGG |
|  | 7 F | CTTCAGCTTATGCTGCATTG |
|  | 7 R | GCCATTAAGATCTTGGTTGTC |
|  | 8 F | AGATTGGTTTGATCCAGTTG |
|  | 8 R | TGAACAAAATTCATGTGGTCC |
|  | 9 F | GTGCTACAACAAAGATTATGC |
|  | 9 R | GAAGCACTGCTTTGATTCTG |
|  | 10 F | CGCAGGATTACAATGTTG |
|  | 10 R | CAATGTTGCATTTTGTTATGC |
|  | 11 F | TTTCCACACACCAGCTTATG |
|  | 11 R | GCTTTGTCACAGTATGGTCC |
|  | 12 F | AAACTGTCACTTGGTGGATC |
|  | 12 R | CACTCTTATTGACAAGACCAAC |
|  | 13 F | ATCGTACACTGAAGCATTAGTAC |
|  | 13 R | GTTAGGTGGCTTAAAGCTTG |
|  | 14 F | GCTAGTGCTTTCAATCAAGC |
|  | 14 R | CATATGCTATAAGCATAGGTCC |
|  | 15 F | AGTGCTAGAACACAAAACTATTAC |
|  | 15 R | TCTTTAAATTTGGCATCTGC |
|  | 16 F | CGAGAGACTTTGTACCCAAAG |
|  | 16 R | TTTTAGACATCGGGTTGCC |
| FIPV | 1 F | AGCGTTGATTATTTCACTCAG |
|  | 1 R | TTAACAAGGACTGAATCTGG |
|  | 2 F | AGGACTGAGGCTGCTAAC |
|  | 2 R | CCACAGCCACACTTAGC |
|  | 3 F | ATGTCATGCCTTTCGTGAAC |
|  | 3 R | AATTACTAGCGATGGCTGTC |
|  | 4 F | ATTGATGCAACTGGTTATATCTG |
|  | 4 R | TGAACCCACAGCATTAAACG |
|  | 5 F | CAAAGGTTTTAACACAACGTG |
|  | 5 R | CCTGGTTCACTTTAAGCAC |
|  | 6 F | CATGTCATTGCTAGTGACAC |
|  | 6 R | TAGGCAAAGCAGCATAAGC |
|  | 7 F | CAATTTAAGTGATGACCCTGAAG |
|  | 7 R | CCTCATGTATAGCCTCATTTTC |
|  | 8 F | CAATGATGGATCTGTGTTATGC |
|  | 8 R | CTTTGTTGTAACAAACAACACC |
|  | 9 F | CACCTGTAACAACGTTACAG |
|  | 9 R | GTTTACACATCCTCTGAGTCAC |
|  | 10 F | AATGTGTACAAACTATGATCTCAG |
|  | 10 R | AAGAATGGCATAGGTTTAAGC |
|  | 11 F | GACATGTACCCAGAGTTCTC |
|  | 11 R | GTACAAAACACAGTCCAATACTC |
|  | 12 F | ATACGTCAAAAGATGGCTTC |
|  | 12 R | GTGACTGTTACAATACGTCAC |
|  | 13 F | GGAAATTGCTATTAGTAAGTGGG |
|  | 13 R | CAAAAGCCTGTGTAATGTTACC |
|  | 14 F | GGTATAACATTAGGTGCACTTG |
|  | 14 R | CAATGCCATCTATGTAGTAACC |
|  | 15 F | TAGAGTTCAGCATTGCTGTG |
|  | 15 R | AAATTTAGCATCAGCTTGAGG |
|  | 16 F | TATGTCCGAGAGATTTTGTTCC |
|  | 16 R | CAATTCCTCGGAAAGACCAG |

1. **Supplemental Figures**


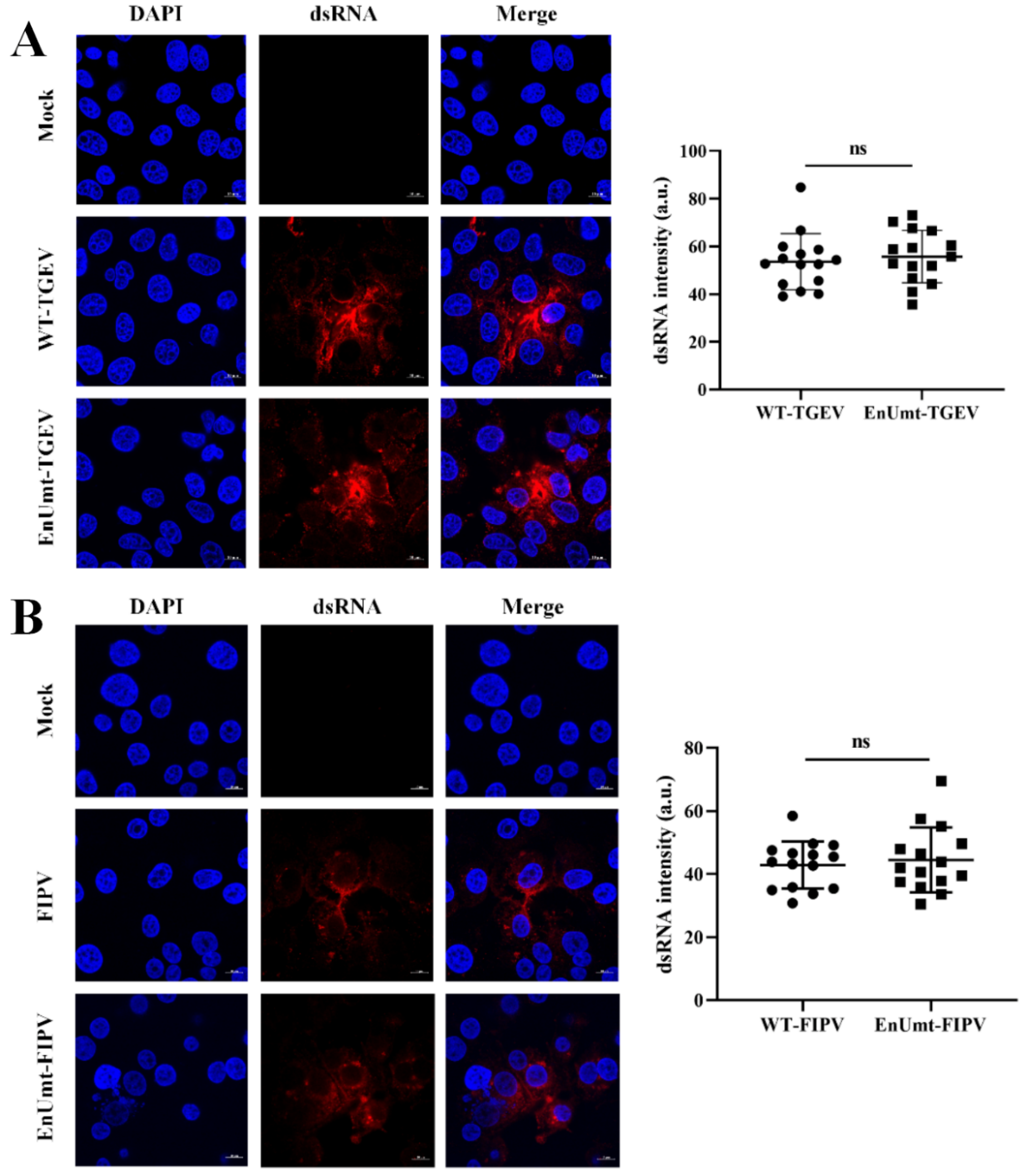


**Supplementary Figure 1 Mutation of nsp15 may not affect the abundance of cytosolic dsRNA in virus-infected pK-15 cells and CRFK cells.**

(A) Confocal microscopy analysis of dsRNA in PK-15 cells infected with WT and EnUmt-TGEV (MOI = 0.01) at 24 hpi. (B) Confocal microscopy analysis of dsRNA in CRFK cells infected with WT and EnUmt-FIPV (MOI = 0.01) at 24 hpi. Scale bar, 10 or 20 µm. Cells were fixed at 24 hpi and stained with anti-dsRNA antibody. Surfaces for puncta were created based on dsRNA fluorescence, and fluorescence was measured within each surface. The foci from 15 images in three independent experiments were counted using the IMARIS software program. The data shown represent the means ± SDs.


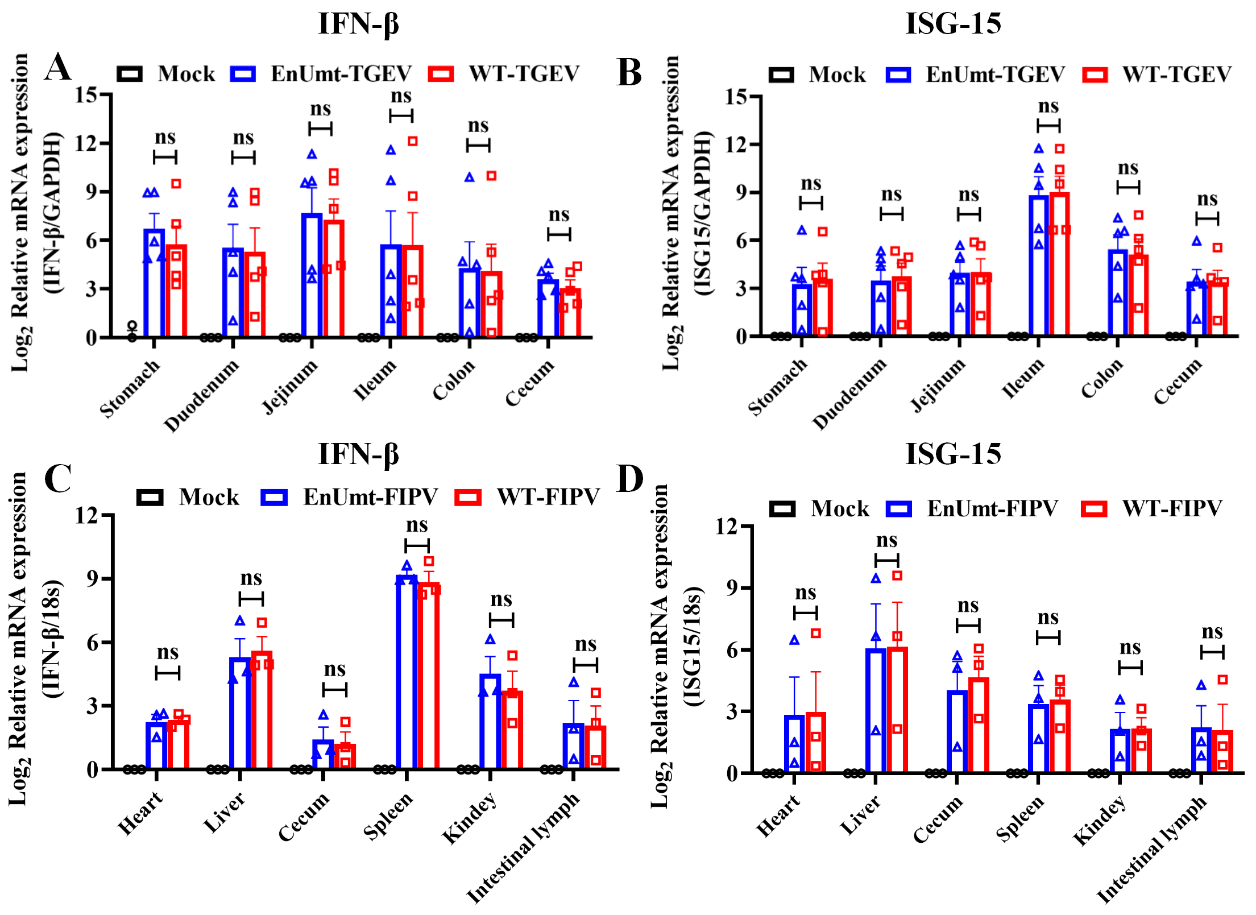


**Supplementary Figure 2 EnUmt-TGEV and EnUmt-FIPV infection induces innate immune response in piglets and cats.**

(A-B) WT- and EnUmt-TGEV infection induces type I IFN response. The tissues (stomach, duodenum, jejunum, ileum, colon, cecum) samples were lysed to collect total RNA for cDNA synthesis, and quantitative PCR was used to measure the relative expression levels of the indicated mRNAs for IFN-β (A) and ISG15 (B). (C-D) WT- and EnUmt-FIPV infection induces type I IFN response. The tissues (heart, liver, cecum, spleen, kidney and intestinal lymph) samples were lysed to collect total RNA for cDNA synthesis, and quantitative PCR was used to measure the relative expression levels of the indicated mRNAs for IFN-β (C) and ISG15 (D). The representative data sets of three independent experiments are shown. The values are presented as the mean ± SD and were analyzed by an unpaired t test. The significant differences are indicated as follows: * p < 0.05, ** p < 0.01.


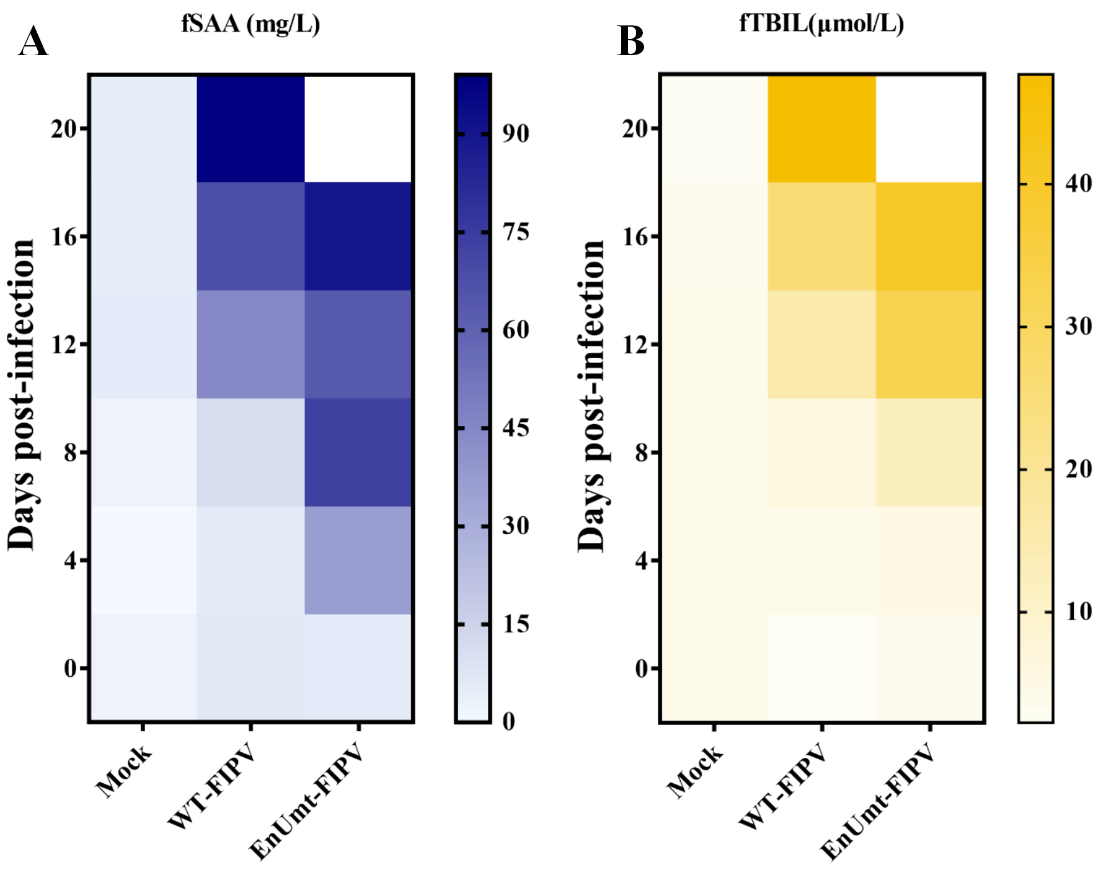


**Supplementary Figure 3** EnUmt-FIPV infection increases virus infection-mediated damage in cats. (A) The serum amyloid A (fSAA) was measured at 0, 4, 8, 12, 16 and 20 days, respectively. The color depth is proportional to the measured value. (B) The total bilirubin (TBIL) was measured at 0, 4, 8, 12, 16 and 20 days, respectively. The color depth is proportional to the measured value.

1. **Supplemental sequence information**

**Genome sequence of TGEV and FIPV:**

**WT-TGEV**

ggttccgtccctatttcgtaagtcgcctagtagtagcgagtgcggttccgcccgtacaacgttgggtagaccgggttccgtcctgtgatctccctcgccggccgccaggagaatgagttccaaacaattcaagatccttgttaatgaggactatcaagtcaacgtgcctagtcttcctattcgtgacgtgttacaggaaattaagtactgctaccgtaatggatttgagggctatgttttcgtaccagaatactgtcgtgacctagttgattgcgatcgtaaggatcactacgtcattggtgttcttggtaacggagtaagtgatcttaaacctgttcttcttaccgaaccctccgtcatgttgcaaggctttattgttagagctaactgcaatggcgttcttgaggactttgaccttaaaattgctcgcactggcagaggtgccatatatgttgatcaatacatgtgtggtgctgatggaaaaccagtcattgaaggcgattttaaggactacttcggtgatgaagacatcattgaatttgaaggagaggagtaccattgcgcttggacaactgtgcgcgatgagaaaccgctgaatcagcaaactctctttaccattcaggaaatccaatacaatctggacattcctcataaattgccaaactgtgctactagacatgtagcaccaccagtcaaaaagaactctaaaatagttctgtctgaagattacaagaagctttatgatatcttcggatcaccctttatgggaaatggtgactgtcttagcaaatgctttgacactcttcattttatcgctgctactcttagatgcccgtgtggttctgaaagtagcggcgttggagattggactggttttaagactgcctgttgtggtctttctggcaaagttaagggtgtcactttgggtgatattaagcctggtgatgctgttgtcactagtatgagcgcaggtaagggagttaagttctttgccaattgtgttcttcaatatgctggtgatgttgaaggtgtctccatctggaaagttattaaaacttttacagttgatgagactgtatgcacccctggttttgaaggcgaattgaacgacttcatcaaacctgagagcaaatcactagttgcatgcagcgttaaaagagcattcattactggtgatattgatgatgctgtacatgattgtatcattacaggaaaattggatcttagtaccaacctttttggtaatgttggtctattattcaagaagactccatggtttgtacaaaagtgtggtgcactttttgtagacgcttggaaagtagtagaggagctttgtggttcactcacacttacatacaagcaaatttatgaagttgtagcatcactttgcacttctgcttttacgattgtaaactacaagccaacatttgtggttccagacaatcgtgttaaagatcttgtagacaagtgtgtgaaagttcttgtaaaagcatttgatgtttttacgcagattatcacaatagctggtattgaggccaaatgctttgtgcttggtgctaaatacctgttgttcaataatgcacttgtcaaacttgtcagtgttaaaatccttggcaagaagcaaaagggtcttgaatgtgcattctttgctactagcttggttggtgcaactgttaatgtgacacctaaaagaacagagactgccactatcagcttgaacaaggttgatgatgttgtagcaccaggagagggttatatcgtcattgttggtgatatggctttctacaagagtggtgaatattatttcatgatgtctagtcctaattttgttcttactaacaatgtttttaaagcagttaaagttccatcttatgacatcgtttatgatgttgataatgataccaaaagcaaaatgattgcaaaacttggttcatcatttgaatatgatggtgatattgatgctgctattgtaaaagtcaatgaactactcattgaatttaggcagcaaagcttgtgcttcagagcttttaaggacgacaaaagcatttttgttgaagcctattttaaaaagtataaaatgccagcatgccttgcaaaacatattggtttgtggaacatcataaagaaagattcatgtaagaggggttttcttaatctcttcaatcacttgaatgaattggaagatatcaaagaaactaatattcaggctattaaaaacattctttgccctgatcctcttcttgatctggattatggtgccatttggtacaattgcatgccaggttgctctgatccttcagttttggggagtgttcaacttttgatcggtaatggtgtgaaagtagtttgtgatggctgcaaaggttttgctaaccaactttcaaaaggttacaacaagctctgtaatgcggctcgcaatgatattgagatcggtggtataccattttccacttttaaaacacctacaaatacttttattgaaatgacagatgctatctattcagttattgaacaaggtaaggcattatcctttagagatgctgatgtgccagttgtagacaatggtaccatttctactgctgattggtctgaacccattctgcttgaacctgctgaatatgtaaaaccaaagaacaatggtaatgtcattgttattgcaggttatacattttataaagatgaggatgaacatttttatccttatggttttggtaaaattgtgcagagaatgtataataaaatgggtggtggtgacaaaactgtctcattttcagaagaagtagatgttcaagaaattgcacctgttacacgtgttaaacttgaattcgaatttgacaatgaaattgtaactggtgttcttgaacgggctattggtactagatacaaatttactggtacaacttgggaagaatttgaagagtctatttctgaagaactcgatgcaatctttgatactctagcaaaccaaggtgtcgaacttgaaggttacttcatttatgacacttgtggtggctttgatataaaaaatccagatggtattatgatctctcagtatgatatcaatattactgctgatgaaaaatcagaagttagtgcatcaagtgaagaagaagaagttgaatctgttgaagaagatcctgagaatgaaattgtagaagcatctgaaggtgctgaagggacttcttctcaagaagaggttgaaacagtagaagttgcagatattacttctacagaagaagatgttgacattgttgaagtatctgctaaagatgacccttgggctgcagctgttgatgtacaagaagctgaacaatttaatccttctctaccacctttcaagacaacgaatctcaacggaaaaattatccttaagcaaggggataataattgttggataaatgcttgttgctatcagcttcaggcctttgattttttcaacaatgaagcttgggagaaatttaagaaaggtgatgtcatggactttgtaaacctttgttatgcagcaacaacactagcaagaggtcattctggtgatgcagagtatcttcttgaacttatgctcaatgattatagcacagccaagatagtacttgcagctaagtgtggttgtggtgaaaaagaaattgttttggaaagagctgtttttaaactcaccccacttaaggagagttttaattatggtgtttgtggtgactgcatgcaagttaacacctgtagatttttaagtgttgaaggctctggtgtttttgttcatgacatattaagcaagcaaacgccagaagctatgtttgttgtcaaacctgttatgcatgcagtttacactggcacaactcaaaatggccattacatggttgatgatattgaacacggttattgtgtagatggtatgggtattaaaccacttaagaaacggtgttatacatccacattgttcattaatgccaatgtaatgactagagctgaaaaaccaaaacaagagtttaaagttgaaaaagtagaacagcaaccgatagtggaggaaaacaaatcctctattgaaaaagaggaaattcaaagtcctaaaaacgatgaccttatacttccattttacaaagctggtaaactttccttttatcagggtgctttggatgttttgatcaatttcttggaacctgatgttattgttaatgctgctaatggtgatcttaaacacatgggtggtgtcgcaagagccatcgatgttttcactggtggcaaattaacagaacgttctaaggattatcttaaaaagaacaaatctattgctcctggtaatgctgttttctttgaaaatgtcattgagcatcttagtgttttgaatgcagttggaccacgtaatggtgacagccgagttgaagccaaactttgtaatgtttacaaagcaattgcaaagtgtgaaggaaaaatattaacaccacttattagtgttggtatctttaatgttagacttgaaacatcattgcagtgcttacttaagactgtgaatgacaggggattgaatgtcttcgtatacactgaccaggagaggcaaactattgagaatttcttctcttgttctatccctgtcaatgttactgaggataatgttaaccatgaacgtgtgtctgtttcttttgacaaaacatacggtgaacagcttaagggcaccgttgtcatcaaagacaaagatgttacaaaccagttgcctagcgcttttgatgttggtcaaaaagttattaaggctattgatatagattggcaagctcattatggtttccgtgatgctgctgcttttagcgctagtagtcatgatgcttataaatttgaagttgttacacatagcaatttcattgtgcataagcagactgacaacaactgttggattaatgcaatttgtcttgcattacagagactcaagccacagtggaaatttcctggtgttagaggtctctggaatgaatttcttgagcgtaaaacacaaggttttgtacatatgttgtatcacatttctggagtaaagaaaggtgagccaggtgatgctgaattaatgctgcataaacttggtgacttgatggacaatgattgtgaaatcattgtcacacacactacagcatgtgacaagtgcgcaaaagtagaaaagtttgttggaccagtggtagcagcacctcttgcaattcatggcactgacgaaacatgtgtgcatggcgttagtgtcaatgtcaaagtcacccaaattaagggcactgttgctattacttctttgattggtcctattattggagaagtactagaagcaactggttatatttgttatagcggttctaacaggaatggtcattacacctattacgataaccgtaatggattagtggttgatgcagaaaaggcttaccattttaatagagacttattacaggtcacaacagctattgcaagtaatttcgttgtcaagaaaccacaagcagaggaaagacctaagaattgtgcttttaacaaagttgcagcatctcctaagattgtacaagaacaaaaattgttggctattgaaagtggtgctaactatgctcttactgaatttggaagatatgctgacatgttctttatggctggagataaaattcttaggttgctgcttgaagtctttaaatatttgctggttttatttatgtgtcttagaagtactaagatgcctaaagttaaagtcaaaccacctcttgcatttaaagattttggtgctaaggtcagaacgctcaattacatgagacaattgaacaaaccctctgtctggcgttacgcaaaactagttttattgttgatagcaatatataatttcttttatttgtttgtcagtataccagtagtgcataaattaacatgtaacggtgctgtacaggcatataaaaattctagttttataaagtctgcagtctgtggcaactctattttatgcaaagcctgtttggcttcttatgatgagttggctgattttcaacatctccaagttacttgggatttcaaatctgacccactatggaacagactggtacaattgtcttactttgcattcttggctgtttttggtaataactatgttaggtgttttcttatgtattttgtatctcagtacctcaacctttggctttcttattttggttatgtagagtacagttggtttttgcatgttgtcaactttgaatccatctcagctgagtttgtgatcgtagttatagtggttaaggcagttctcgcccttaaacatattgttttcgcatgctcaaacccgtcttgcaaaacgtgctctaggactgcaaggcagacacgtattcctattcaagttgttgttaatggttcaatgaagactgtttatgttcatgctaatggtactggtaaattctgcaagaaacacaatttttattgtaagaactgtgattcttatggctttgaaaacacattcatctgtgacgaaattgtacgtgatctcagtaatagtgttaaacaaactgtttacgccactgatagatctcatcaagaagtcacaaaagttgaatgttcagatggcttttacagattttatgttggtgatgaattcacttcatatgactatgatgtaaaacacaagaaatacagtagtcaagaggttctcaagagcatgctcttgcttgatgacttcattgtgtacagtccatctggttctgctcttgcaaatgttagaaatgcctgtgtttacttttcacaacttattggtaagcctattaagattgttaacagtgatttgcttgaagacctctctgtagattttaaaggggcactttttaatgctaaaaagaatgtaattaagaattctttcaatgttgatgtctcagaatgcaaaaatcttgacgaatgttacagggcttgcaatcttaatgtttcattttctacatttgaaatggctgtcaacaatgctcataggtttggtattctgattactgatcgttcttttaacaatttctggccatcaaaagttaagcctggttcatctggtgtgtcggccatggacattggtaagtgtatgacttctgatgctaagattgttaatgctaaagttttaactcaacgtggtaaaagtgttgtttggcttagccaggattttgctgcacttagctcaactgctcagaaagttttggttaaaacttttgtagaagaaggtgtcaacttttcactcacatttaatgctgtaggttcagatgatgatcttccttatgaaagattcactgaatctgtgtctccaaaaagtggttcaggctttttcgatgtaattacacagcttaaacaaattgtgattttggtttttgtttttatctttatttgtggtttgtgctctgtttacagtgttgctacacagtcctacattgaatctgctgaaggctatgactacatggttattaagaatggaattgttcaaccttttgacgataccatttcatgtgttcataacacttataaaggattcggtgactggtttaaagctaagtatggttttatccctacttttggtaaatcatgtccaattgttgtaggaactgtttttgatcttgaaaatatgagaccaattcctgacgtgcctgcatatgtttctattgtgggtagatctcttgttttcgctattaatgctgcttttggtgttactaatatgtgctatgatcatactggcaatgcagttagtaaggactcttactttgatacttgtgtgtttaatactgcgtgcaccactcttacaggtcttggtggtacaattgtatattgtgcaaagcaaggtttagttgaaggtgctaagctctatagtgatcttatgccagactattattatgagcatgctagtggtaacatggttaaattgccagcaattattagaggacttggtctacgttttgtgaaaacacaggctacaacttattgtagagtgggagagtgcattgatagtaaagctggtttttgctttggtggcgataactggtttgtctacgacaatgagtttggcaatggatacatctgtggtaattctgtgctaggattctttaagaatgtcttcaaactctttaactctaacatgtctgtggtagctacatctggtgcgatgcttgttaacattattattgcatgcttagctattgcaatgtgttatggtgttcttaagtttaagaagatttttggtgattgtactttcctcattgttatgatcattgtcacccttgttgtgaacaatgtgtcttattttgtcactcaaaacacgttctttatgatcatctacgccattgtttactattttataacaagaaaacttgcatacccaggcattcttgatgctgggtttattattgcttatattaatatggctccatggtacgtgattaccgcatatatcctagttttcctctatgactcactcccttcactgtttaaacttaaagtttcaacaaatctttttgaaggtgataaatttgtgggtaactttgaatctgctgctatgggtacttttgttattgacatgcgttcatatgaaactattgttaattctacttctattgctagaattaaatcatatgctaacagcttcaataaatataagtactacacaggttcaatgggagaagctgactacagaatggcttgctatgctcatcttggtaaagctcttatggactattctgttaatagaacagacatgctttacacacctcctactgttagtgttaattctacacttcagtcaggtttgcggaaaatggcacagcctagtggtcttgtagagccttgcattgtaagagtttcctatggtaacaatgtgcttaatggtttatggttaggagatgaagtcatttgccctagacatgttattgctagtgataccacacgtgttatcaactatgaaaatgaaatgtctagtgtgagacttcacaacttttcagtttctaagaataatgtgtttttgggtgttgtgtctgccagatataagggtgtgaatcttgtacttaaagtcaaccaggttaatcctaacacaccagaacataaatttaagtctattaaagctggtgaaagttttaacattcttgcttgttatgaaggatgtcctggcagtgtttatggtgtcaacatgagaagtcaaggtaccattaaaggatcttttatagctggtacttgtggatcagtaggttatgtgttagaaaatggaattctctattttgtatacatgcatcacttagaacttggaaatggctcgcatgttggttccaattttgaaggagaaatgtacggtggttatgaagatcaacctagcatgcaattggaaggtactaatgtcatgtcatcagataatgtggttgcattcctatatgctgcacttatcaatggtgaaaggtggtttgttacaaacacatcgatgtcattagaatcatacaatacatgggccaaaactaacagtttcacagaactttcttcaactgatgcttttagcatgttggctgcaaaaactggtcaaagtgttgagaaattactagatagcatcgtaagactcaacaagggttttggaggtcgtactatactttcttatggctcattgtgtgacgagttcactccaactgaagtcataaggcaaatgtatggtgtaaatcttcaggctggtaaagtaaaatctttcttctaccctattatgactgcaatgacaattctctttgccttttggcttgaattctttatgtacacacccttcacttggattaatccaacttttgttagcattgtattggctgttacaactttgatctcgacggtttttgtctctggcatcaaacataagatgttgttctttatgtcttttgtccttcctagtgttatccttgtgacagcacacaatttgttctgggacttttcttactatgaaagtcttcagtcaattgttgagaatactaacactatgtttttgcctgttgacatgcaaggtgtcatgctcacagtgttttgctttattgtctttgttacatatagtgttagattcttcacttgcaaacaatcatggttctcacttgctgtgacaactattcttgtgatctttaacatggttaaaatctttggaacatctgatgaaccatggactgaaaaccaaattgctttctgctttgtgaacatgcttactatgattgtcagtcttactacaaaggattggatggttgtcattgcatcatacagaattgcatattatattgttgtatgtgtaatgccatctgcttttgtatctgactttgggtttatgaagtgtattagcattgtttacatggcgtgcggttatttgttttgttgctattatggcattctttattgggttaacagatttacatgcatgacttgtggtgtttatcaattcactgtgtctgcagctgaacttaaatacatgaccgctaacaacctttctgcacctaagaacgcatatgacgctatgattcttagtgctaaattgattggtgttggaggtaagagaaacatcaaaatttcaactgtacagtcaaaacttacagagatgaaatgtaccaatgttgtcttgcttggtcttttatctaaaatgcatgtcgagtctaactcaaaagagtggaactattgtgttggactacacaatgagataaacctttgtgacgatcctgaaatcgttcttgagaaactgttagctcttattgcattcttcttgtccaaacataacacttgtgaccttagcgaacttattgaatcatactttgagaacaccaccatactccagagtgtggcttcagcttatgctgcattgcctagctggattgcacttgaaaaagctcgcgctgatcttgaagaggctaagaaaaatgatgttagccctcaaattttgaagcagcttactaaagcatttaacattgccaagagtgattttgagcgcgaagcatcagtgcaaaagaaactcgacaaaatggctgagcaggctgcagctagtatgtataaagaagcacgagctgtggacagaaagtcaaagattgtttctgctatgcatagcctactttttggtatgcttaagaaacttgatatgtccagtgtcaacactattattgaccaggctcgtaatggtgttctacctttaagtatcattccagctgcatcagctacaagacttgttgttattacacctagccttgaagtgttttccaagattaggcaagaaaacaatgttcattatgctggtgctatttggactattgttgaagttaaagatgctaatggttcacatgtacatcttaaggaagtcaccgctgctaatgaattaaaccttacttggccattgagcattacttgtgagagaaccacaaagcttcagaacaatgaaattatgccaggtaaacttaaagaaagagctgtcagagcgtcagcaactcttgatggtgaagctttcggcagtggaaaggctcttatggcatctgaaagtggaaaaagctttatgtatgcatttatagcctcagacaacaatcttaagtatgttaagtgggagagcaataatgatattatacctattgaacttgaagctccattgcgtttctatgttgacggcgctaatggtcctgaagtcaagtatttgtattttgtcaagaatttaaacactcttagacgtggtgccgttcttggttatatcggtgcaacagttcgtctgcaagctggtaaacccactgaacatccatctaacagtagtttattgacattgtgtgctttttcacctgatcctgctaaagcatatgttgatgctgttaagagaggcatgcaaccagttaataactgtgtaaaaatgctctcaaatggtgctggtaatggtatggctgttacaaacggtgtcgaagctaacacacaacaggactcttatggtggtgcttcagtttgtatttattgcagatgccatgttgaacatcctgctattgatggattatgccgctacaaaggtaagttcgtgcaaataccaactggcacacaagatccaattcggttctgtattgaaaatgaagtttgtgttgtctgtggttgttggcttaacaatggttgcatgtgcgatcgtacttctatgcagagttttactgttgatcaaagttatttaaacgagtgcggggttctagtgcagctcgactagaaccctgcaatggtactgatccagaccatgttagtagagcttttgacatctacaacaaagatgttgcgtgtattggtaaattccttaagacgaattgttcaagatttaggaatttggacaaacatgatgcctactacattgtcaaacgttgtacaaagaccgttatggaccatgagcaagtctgttataacgatcttaaagattctggtgctgttgctgagcatgacttcttcacatataaagagggtagatgtgagttcggtaatgttgcacgtaggaatcttacaaagtacacaatgatggatctttgttacgctatcagaaattttgatgaaaagaactgtgaagttctcaaagaaatactcgtgacagtaggtgcttgcactgaagaattctttgaaaataaagattggtttgatccagttgaaaatgaagccatacatgaagtttatgcaaaacttggacccattgtagccaatgctatgcttaaatgtgttgctttttgcgatgcgatagtggaaaaaggctatataggtgttataacacttgacaaccaagatcttaatggcaatttctacgatttcggcgatttcgtgaagactgctccgggttttggttgcgcttgtgttacatcatattattcttatatgatgcctttaatggggatgacttcatgcttagagtctgaaaactttgtgaaaagtgacatctatggttctgattataagcagtatgatttactagcttatgattttaccgaacataaggagtaccttttccaaaaatactttaagtactgggatcgcacatatcacccaaattgttctgattgtactagtgacgagtgtattattcattgtgctaattttaacacattgttttctatgacaataccaatgacagcttttggaccacttgtccgtaaagttcatattgatggtgtaccagtagttgttactgcaggttaccatttcaaacaacttggtatagtatggaatcttgatgtaaaattagacacaatgaagttgagcatgactgatcttcttagatttgtcacagatccaacacttcttgtagcatcaagccctgcacttttagaccagcgtactgtctgtttctccattgcagctttgagtactggtattacatatcagacagtaaaaccaggtcactttaacaaggatttctacgatttcataacagagcgtggattctttgaagagggatctgagttaacattaaaacattttttctttgcacagggtggtgaagctgctatgacagacttcaattattatcgctacaatagagtcacagtacttgatatttgccaagctcaatttgtttacaaaatagttggcaagtattttgaatgttatgacggtgggtgcattaatgctcgtgaagttgttgttacaaactatgacaagagtgctggctatcctttgaacaaatttggtaaagctagactttactacgaaactctttcatatgaagagcaggatgcactttttgctttaacaaagagaaatgttttacccacaatgactcaaatgaatttgaaatacgctatttctggtaaggcaagagctcgtacagtaggaggagtttcacttctttctaccatgactacgagacaatatcatcagaagcatttgaagtcaattgctgcaacacgcaatgctactgtggtcattggttcaaccaagttttatggtggttgggacaatatgcttaaaaatttaatgcgtgatgttgataatggttgtttgatgggatgggactatcctaagtgtgaccgtgctttacctaatatgattagaatggcttctgccatgatattaggttctaagcatgttggttgttgtacacataatgataggttctaccgcctctccaatgagttagctcaagtactcacagaagttgtgcattgcacaggtggtttttattttaaacctggtggtacaactagcggtgatggtactacagcatatgctaactctgcttttaacatctttcaagctgtttctgctaatgttaataagcttttgggggttgattcaaacgcttgtaacaacgttacagtaaaatccatacaacgtaaaatttacgataattgttatcgtagtagcagcattgatgaagaatttgttgttgagtactttagttatttgagaaaacacttttctatgatgattttatctgatgatggagttgtgtgctacaacaaagattatgcggatttaggttatgtagctgacattaatgcttttaaagcaacactttattaccagaataacgtctttatgtccacttctaagtgttgggtagaaccagatcttagtgttggaccacatgaattttgttcacagcatacattgcagattgttgggcctgatggagactactatcttccctatccagacccgtccagaattttgtcagctggtgtgtttgttgatgacatagttaaaacagacaatgttattatgttagaacgttacgtgtcattggctattgacgcatacccgctcacaaaacaccctaagcctgcttatcaaaaagtgttttacactctactagattgggttaaacatctacagaaaaatttgaatgcaggtgttcttgattcgttttcagtgacaatgttagaggaaggtcaagataagttctggagtgaagagttttacgctagcctctatgaaaagtccactgtcttgcaagctgcaggcatgtgtgtagtatgtggttcgcaaactgtacttcgttgtggagactgtcttaggagaccacttttatgcacgaaatgtgcttacgaccatgttatgggaacaaagcataaattcattatgtctatcacaccatatgtgtgtagttttaatggttgtaatgtcaatgatgttacaaagttgtttttaggtggtcttagttattattgtatgaaccacaaaccacagttgtcattcccactctgtgctaatggcaacgtttttggtctatataaaagtagtgcagtcggctcagaggctgttgaagatttcaacaaacttgcagtttctgactggactaatgtagaagactacaaacttgctaacaatgtcaaggaatctctgaaaattttcgctgctgaaactgtgaaagctaaggaggagtctgttaaatctgaatatgcttatgctgtattaaaggaggttatcggccctaaggaaattgtactccaatgggaagcttctaagactaagcctccacttaacagaaattcagttttcacgtgttttcagataagtaaggatactaaaattcaattaggtgaatttgtgtttgagcaatctgagtacggtagtgattctgtttattacaagagcacgagtacttacaaattgacaccaggtatgatttttgtgttgacttctcataatgtgagtcctcttaaagctccaattttagtcaaccaagaaaagtacaataccatatctaagctctatcctgtctttaatatagcggaggcctataatacactggttccttactaccaaatgataggtaagcaaaaatttacaactatccaaggtcctcctggtagcggtaaatctcattgtgttataggtttgggtttgtattaccctcaggcgagaatagtctacactgcatgttctcatgcggctgtagacgctttatgtgaaaaagcagccaaaaacttcaatgttgatagatgttcaaggataatacctcaaagaatcagagttgattgttacacaggctttaagcctaataacaccaatgcgcagtacttgttttgtactgttaatgctctaccagaagcaagttgtgacattgttgtagttgatgaggtctctatgtgtactaattatgatcttagtgtcataaatagccgactgagttacaaacatattgtttatgttggagacccacagcagctaccagctcctagaactttgattaataagggtgtacttcaaccgcaggattacaatgttgtaaccaaaagaatgtgcacactaggacctgatgtctttttgcataaatgttacaggtgcccagctgaaattgttaagacagtctctgcacttgtttatgaaaataaatttgtacctgtcaacccagaatcaaagcagtgcttcaaaatgtttgtaaaaggtcaggttcagattgagtctaactcttctataaacaacaagcaactagaggttgtcaaggcctttttagcacataatccaaaatggcgtaaagctgttttcatctcaccctataatagtcaaaattatgttgctcggcgtcttcttggtttgcaaacgcaaactgtggattccgctcagggtagtgagtatgattacgtcatctacacacagacctccgatacacagcatgctactaatgttaacagatttaatgttgccattacgagagcaaaggttggtatactttgtatcatgtgtgatagaactatgtatgagaatcttgatttctatgaactcaaagattcaaagattggtttacaagcaaaacctgaaacttgtggtttatttaaagattgttcgaagagcgaacaatacataccacctgcttatgcaacgacatatatgagcttatctgataattttaagacaagtgatggtttagctgttaacatcggtacaaaagatgttaaatatgctaatgtcatctcatatatgggattcaggtttgaagccaacataccaggctatcacacactattctgcacgcgagattttgctatgcgtaatgttagagcatggcttgggtttgacgttgaaggtgcacatgtctgtggtgataatgttggaactaatgtaccattacagctgggtttctcaaacggtgtggattttgtagtgcaaactgaaggatgtgttattactgaaaaaggtaatagcattgaggttgtaaaagcacgagcaccaccaggtgagcaatttgcacacttgattccgcttatgagaaagggtcaaccttggcacattgttagacgccgtatagtgcagatggtctgtgactattttgatggcttatcagacattctgatctttgtgctttgggctggtggtcttgaacttacaactatgagatactttgttaaaattggaagaccacaaaaatgtgaatgcggcaaaagtgcaacttgttatagtagctctcaatctgtttatgcttgcttcaagcatgcattaggatgtgattatttatataacccttactgcattgacatacagcaatggggttacacaggatctttgagcatgaatcatcatgaagtttgcaacattcatagaaatgagcatgtagctagtggtgatgctatcatgactagatgtctcgctatacatgactgttttgtcaaacgtgttgattggtcaattgtgtacccttttattgacaatgaagaaaagatcaataaagctggtcgcatagtgcagtcacatgtcatgaaagctgctctgaagatttttaatcctgctgcaattcacgatgtgggtaatccaaaaggcatccgttgtgctacaacaccaataccatggttttgttatgatcgtgatcctattaataacaatgttagatgtctggattatgactatatggtacatggtcaaatgaatggtcttatgttattttggaactgtaatgtagacatgtacccagagttttcaattgtttgtagatttgatactcgcactcgctctaaattgtctttagaaggttgtaatggtggtgcattgtatgttaataaccatgctttccacacaccagcttatgatagaagagcttttgctaagcttaaacctatgccattcttttactatgatgatagtaattgtgaacttgttgatgggcaacctaattatgtaccacttaagtcaaatgtttgcataacaaaatgcaacattggtggtgctgtctgcaagaagcatgctgctctttacagagcgtatgttgaggattacaacatttttatgcaggctggttttacaatatggtgtcctcaaaactttgacacctatatgctttggcatggttttgttaatagcaaagcacttcagagtctagaaaatgtggcttttaatgtcgttaagaaaggtgccttcaccggtttaaaaggtgacttaccaactgctgttattgctgacaaaataatggtaagagatggacctactgacaaatgtatttttacaaataagactagtttacctacaaatgtagcttttgagttatatgcaaaacgcaaacttggactcacacctccattaacaatacttaggaatttaggtgttgtcgcaacatataagtttgtgttgtgggattatgaagctgaacgtcctttctcaaatttcactaagcaagtgtgttcctacactgatcttgatagtgaagttgtaacatgttttgataatagtattgctggttcttttgagcgttttactactacaagagatgcagtgcttatttctaataacgctgtgaaagggcttagtgccattaaattacaatatggccttttgaatgatctacctgtaagtactgttggaaataaacctgtcacatggtatatctatgtgcgcaagaatggtgagtacgtcgaacaaatcgatagttactatacacagggacgtacttttgaaaccttcaaacctcgtagtacaatggaagaagattttcttagtatggatactacactcttcatccaaaagtatggtcttgaggattatggttttgaacacgttgtatttggagatgtctctaaaactaccattggtggtatgcatcttcttatatcgcaagtgcgccttgcaaaaatgggtttgttttccgttcaagaatttatgaataattctgacagtacactgaaaagttgttgtattacatatgctgatgatccatcttctaagaatgtgtgcacttatatggacatactcttggacgattttgtgactatcattaagagcttagatcttaatgttgtgtccaaagttgtggatgtcattgtagattgtaaggcatggagatggatgttgtggtgtgagaattcacatattaaaaccttctatccacaactccaatctgctgaatggaatcccggctatagcatgcctacactgtacaaaatccagcgtatgtgtctcgaacggtgtaatctctacaattatggtgcacaagtgaaattacctgatggcattactactaatgtcgttaagtatactcagttgtgtcaataccttaacactactacattgtgtgtaccacacaaaatgcgtgtattgcatttaggagctgctggtgcatctggtgttgctcctggtagtactgtattaagaagatggttaccagatgatgccatattggttgataatgatttgagagattacgtttccgacgcagacttcagtgttacaggtgattgtactagtctttacatcgaagacaagtttgatttgctcgtctctgatttatatgatggctccacaaaatcaattgacggtgaaaacacgtcgaaagatggtttctttacttatattaatggtttcattaaagagaaactgtcacttggtggatctgttgccattaaaatcacggaatttagttggaataaagatttatatgaattgattcaaagatttgagtattggactgtgttttgtacaagtgttaacacgtcatcatcagaaggctttctgattggtattaactacttaggaccatactgtgacaaagcaatagtagatggaaatataatgcatgccaattatatattttggagaaactctacaattatggctctatcacataactcagtcctagacactcctaaattcaagtgtcgttgtaacaacgcacttattgttaatttaaaagaaaaagaattgaatgaaatggtcattggattactaaggaagggtaagttgctcattagaaataatggtaagttactaaactttggtaaccacttcgttaacacaccatgaaaaaactatttgtggttttggtcgtaatgccattgatttatggagacaattttccttgttctaaattgactaatagaactataggcaaccagtggaatctcattgaaaccttccttctaaactatagtagtaggttaccacctaattcagatgtggtgttaggtgattattttcctactgtacaaccttggtttaattgcattcgcaatgatagtaatgacctttatgttacactggaaaatcttaaagcattgtattgggattatgctacagaaaatatcacttggaatcacagacaacggttaaacgtagtcgttaatggatacccatactccatcacagttacaacaacccgcaattttaattctgctgaaggtgctattatatgcatttgtaagggctcaccacctactaccaccacagaatctagtttgacttgcaattggggtagtgagtgcaggttaaaccataagttccctatatgtccttctaattcagaggcaaattgtggtaatatgctgtatggcctacaatggtttgcagatgaggttgttgcttatttacatggtgctagttaccgtattagttttgaaaatcaatggtctggcactgtcacatttggtgatatgcgtgcgacaacattagaagtcgctggcacgcttgtagacctttggtggtttaatcctgtttatgatgtcagttattatagggttaataataaaaatggtactaccgtagtttccaattgcactgatcaatgtgctagttatgtggctaatgtttttactacacagccaggaggttttataccatcagattttagttttaataattggttccttctaactaatagctccacgttggttagtggtaaattagttaccaaacagccgttattagttaattgcttatggccagtccctagctttgaagaagcagcttctacattttgttttgagggtgctggctttgatcaatgtaatggtgctgttttaaataatactgtagacgtcattaggttcaaccttaattttactacaaatgtacaatcaggtaagggtgccacagtgttttcattgaacacaacgggtggtgtcactcttgaaatttcatgttatacagtgagtgactcgagctttttcagttacggtgaaattccgttcggcgtaactgatggaccacggtactgttacgtacactataatggcacagctcttaagtatttaggaacattaccacctagtgtcaaggagattgctattagtaagtggggccatttttatattaatggttacaatttctttagcacatttcctattgattgtatatcttttaatttgaccactggtgatagtgacgttttctggacaatagcttacacatcgtacactgaagcattagtacaagttgaaaacacagctattacaaaggtgacgtattgtaatagtcacgttaataacattaaatgctctcaaattactgctaatttgaataatggattttatcctgtttcttcaagtgaagttggtcttgtcaataagagtgttgtgttactacctagcttttacacacataccattgttaacataactattggtcttggtatgaagcgtagtggttatggtcaacccatagcctcaacattaagtaacatcacactaccaatgcaggatcacaacaccgatgtgtactgtattcgttctgaccaattttcagtttatgttcattctacttgcaaaagtgctttatgggacaatatttttaagcgaaactgcacggacgttttagatgccacagctgttataaaaactggtacttgtcctttctcatttgataaattgaacaattacttaacttttaacaagttctgtttgtcgttgagtcctgttggtgctaattgtaagtttgatgtagctgcccgtacaagaaccaatgagcaggttgttagaagtttgtatgtaatatatgaagaaggagacaacatagtgggtgtaccgtctgataatagtggtgtgcacgatttgtcagtgctacacctagattcctgcacagattacaatatatatggtagaactggtgttggtattattagacaaactaacaggacgctacttagtggcttatattacacatcactatcaggtgatttgttaggttttaaaaatgttagtgatggtgtcatctactctgtaacgccatgtgatgtaagcgcacaagcagctgttattgatggtaccatagttggggctatcacttccattaacagtgaactgttaggtctaacacattggacaacaacacctaatttttattactactctatatataattacacaaatgataggactcgtggcactgcaattgacagtaatgatgttgattgtgaacctgtcataacctattctaacataggtgtttgtaaaaatggtgcttttgtttttattaacgtcacacattctgatggagacgtgcaaccaattagcactggtaatgtcacgatacctacaaactttaccatatccgtgcaagtcgaatatattcaggtttacactacaccagtgtcaatagactgttcaagatatgtttgtaatggtaaccctaggtgtaacaaattgttaacacaatacgtttctgcatgtcaaactattgagcaagcacttgcaatgggtgccagacttgaaaacatggaggttgattccatgttgtttgtttctgaaaatgcccttaaattggcatctgttgaagcattcaatagttcagaaactttagaccctatttacaaagaatggcctaatataggtggttcttggctagaaggtctaaaatacatacttccgtcccataatagcaaacgtaagtatcgttcagctatagaggacttgctttttgataaggttgtaacatctggtttaggtacagttgatgaagattataaacgttgtacaggtggttatgacatagctgacttagtatgtgctcaatactataatggcatcatggtgctacctggtgtggctaatgctgacaaaatgactatgtacacagcatcccttgcaggtggtataacattaggtgcacttggtggaggcgccgtggctataccttttgcagtagcagttcaggctagacttaattatgttgctctacaaactgatgtattgaacaaaaaccagcagattctggctagtgctttcaatcaagctattggtaacattacacagtcatttggtaaggttaatgatgctatacatcaaacatcacgaggtcttgctactgttgctaaagcattggcaaaagtgcaagatgttgtcaacatacaagggcaagctttaagccacctaacagtacaattgcaaaataatttccaagccattagtagttctattagtgacatttataataggcttgacgaattgagtgctgatgcacaagttgacaggctgatcacaggaagacttacagcacttaatgcatttgtgtctcagactctaaccagacaagcggaggttagggctagtagacaacttgccaaagacaaggttaatgaatgcgttaggtctcagtctcagagattcggattctgtggtaatggtacacatttgttttcactcgcaaatgcagcaccaaatggcatgattttctttcacacagtgctattaccaacggcttatgaaactgtgactgcttggccaggtatttgtgcttcagatggtgatcgcacttttggacttgtcgttaaagatgtccagttgactttgtttcgtaatctagatgacaagttctatttgacccccagaactatgtatcagcctagagttgcaactagttctgactttgttcaaattgaagggtgcgatgtgctgtttgttaatgcaactgtaagtgatttgcctagtattatacctgattatattgatattaatcagactgttcaagacatattagaaaattttagaccaaattggactgtacctgagttgacatttgacatttttaacgcaacctatttaaacctgactggtgaaattgatgacttagaatttaggtcagaaaagctacataacaccactgtagaacttgccattctcattgacaacattaacaatacattagtcaatcttgaatggctcaatagaattgaaacctatgtaaaatggccttggtatgtgtggctactaataggcttagtagtaatattttgcataccattactgctattttgctgttgtagtacaggttgctgtggatgcataggttgtttaggaagttgttgtcactctatatgtagtagaagacaatttgaaaattacgaaccaattgaaaaagtgcacgtccattaaatttaaaatgttaattctatcatctgctataatagcagttgtttctgctagagaattttgttaaggatgatgaataaagtctttaagaactaaacttacgagtcattacaggtcctgtatggacattgtcaaatccatttacacatccgtagatgctgtacttgacgaacttgattgtgcatactttgctgtaactcttaaagtagaatttaagactggtaaattacttgtgtgtataggttttggtgacacacttcttgctgctaaggataaagcatatgctaagcttggtctctccattattgaagaagtcaatagtcatatagttgtttaatatcattaaacacacaaaacccaaagcattaagtgttacaaaacaattaaagagagattatagaaaaactgtcattctaaattccatgcgaaaatgattggtggactttttcttagtactctgagttttgtaattgttagtaaccattctattgttaataacacagcaaatgtgcatcatatacaacaagaacgtgttatagtacaacagcatcaggttgttagtgctagaacacaaaactattacccagagttcagcatcgctgtactctttgtatcttttctagctttgtaccgtagtacaaactttaagacgtgtgtcggcatcttaatgtttaagattttatcaatgacacttttaggacctatgcttatagcatatggttactacattgatggcattgttacaacaactgtcttatctttaagatttgtctacttagcatacttttggtatgttaatagtaggtttgaatttattttatacaatacaacgacactcatgtttgtacatggcagagctgcaccgtttatgagaagttctcacagctctatttatgtcacattgtatggtggcataaattatatgtttgtgaatgacctcacgttgcattttgtagaccctatgcttgtaagcatagcaatacgtggcttagctcatgctgatctaactgtagttagagcagttgaacttctcaatggtgattttatttatgtattttcacaggagcccgtagtcggtgtttacaatgcagccttttctcaggcggttctaaacgaaattgacttaaaagaagaagaagaagaccatacctatgacgtttcctagggcattgactgtcatagatgacaatggaatggtcattaacatcattttctggttcctgttgataattatattgatattactttcaatagcattgctaaatataattaagctatgcatggtgtgttgcaatttaggaaggacagttattattgttccagcgcaacatgcttacgatgcctataagaattttatgcgaattaaagcatacaaccccgatggagcactccttgcttgaactaaacaaaatgaagattttgttaatattagcgtgtgtgattgcatgcgcatgtggagaacgctattgtgctatgaaatccgatacagatttgtcatgtcgcaatagtacagcgtctgattgtgagtcatgcttcaacggaggcgatcttatttggcatcttgcaaactggaacttcagctggtctataatattgatcgtttttataactgtgctacaatatggaagacctcaattcagctggttcgtgtatggcattaaaatgcttataatgtggctattatggcccgttgttttggctcttacgatttttaatgcatactcggaataccaagtgtccagatatgtaatgttcggctttagtattgcaggtgcaattgttacatttgtactctggattatgtattttgtaagatccattcagttgtacagaaggactaagtcttggtggtctttcaaccctgaaactaaagcaattctttgcgttagtgcattaggaagaagctatgtgcttcctctcgaaggtgtgccaactggtgtcactctaactttgctttcagggaatttgtacgctgaagggttcaaaattgcaggtggtatgaacatcgacaatttaccaaaatacgtaatggttgcattacctagcaggactattgtctacacacttgttggcaagaagttgaaagcaagtagtgcgactggatgggcttactatgtaaaatctaaagctggtgattactcaacagaggcaagaactgataatttgagtgagcaagaaaaattattacatatggtataactaaacttctaaatggccaaccagggacaacgtgtcagttggggagatgaatctaccaaaacacgtggtcgttccaattcccgtggtcggaagaataataacatacctctttcattcttcaaccccataaccctccaacaaggttcaaaattttggaacttatgtccgagagactttgtacccaaaggaataggtaacagggatcaacagattggttattggaatagacaaactcgctatcgcatggtgaagggccaacgtaaagagcttcctgaaaggtggttcttctactacttaggtactggacctcatgcagatgccaaatttaaagataaattagatggagttgtctgggttgccaaggatggtgccatgaacaaaccaaccacgcttggtagtcgtggtgctaataatgaatccaaagctttgaaattcgatggtaaagtgccaggcgaatttcaacttgaagttaatcaatcaagagacaattcaaggtcacgctctcaatctagatctcggtctagaaatagatctcaatctagaggcaggcaacaattcaataacaagaaggatgacagtgtagaacaagctgttcttgccgcacttaaaaagttaggtgttgacacagaaaaacaacagcaacgctctcgttctaaatctaaagaacgtagtaactctaagacaagagatactacacctaagaatgaaaacaaacacacctggaagagaactgcaggtaaaggtgatgtgacaagattttatggagctagaagcagttcagccaattttggtgacactgacctcgttgccaatgggagcagtgccaagcattacccacaactggctgaatgtgttccatctgtgtctagcattctgtttggaagctattggacttcaaaggaagatggcgaccagatagaagtcacgttcacacacaaataccacttgccaaaggatgatcctaagactggacaattccttcagcagattaatgcctatgctcgtccatcagaagtggcaaaagaacagagaaaaagaaaatctcgttctaaatctgcagaaaggtcagagcaagatgtggtacctgatgcattaatagaaaattatacagatgtgtttgatgacacacaggttgagataattgatgaggtaacgaactaaacgagatgctcgtcttcctccatgctgtatttattacagttttaatcttactactaattggtagactccaattattagaaagactattacttaatcactctttcaatcttaaaactgtcaatgactttaatatcttatataggagtttagcagaaaccagattactaaaagtggtgcttcgagtaatctttctagtcttactaggattttgctgctacagattgttagtcacattaatgtaaggcaacccgatgtctaaaa

**EnUmt-TGEV**

ggttccgtccctatttcgtaagtcgcctagtagtagcgagtgcggttccgcccgtacaacgttgggtagaccgggttccgtcctgtgatctccctcgccggccgccaggagaatgagttccaaacaattcaagatccttgttaatgaggactatcaagtcaacgtgcctagtcttcctattcgtgacgtgttacaggaaattaagtactgctaccgtaatggatttgagggctatgttttcgtaccagaatactgtcgtgacctagttgattgcgatcgtaaggatcactacgtcattggtgttcttggtaacggagtaagtgatcttaaacctgttcttcttaccgaaccctccgtcatgttgcaaggctttattgttagagctaactgcaatggcgttcttgaggactttgaccttaaaattgctcgcactggcagaggtgccatatatgttgatcaatacatgtgtggtgctgatggaaaaccagtcattgaaggcgattttaaggactacttcggtgatgaagacatcattgaatttgaaggagaggagtaccattgcgcttggacaactgtgcgcgatgagaaaccgctgaatcagcaaactctctttaccattcaggaaatccaatacaatctggacattcctcataaattgccaaactgtgctactagacatgtagcaccaccagtcaaaaagaactctaaaatagttctgtctgaagattacaagaagctttatgatatcttcggatcaccctttatgggaaatggtgactgtcttagcaaatgctttgacactcttcattttatcgctgctactcttagatgcccgtgtggttctgaaagtagcggcgttggagattggactggttttaagactgcctgttgtggtctttctggcaaagttaagggtgtcactttgggtgatattaagcctggtgatgctgttgtcactagtatgagcgcaggtaagggagttaagttctttgccaattgtgttcttcaatatgctggtgatgttgaaggtgtctccatctggaaagttattaaaacttttacagttgatgagactgtatgcacccctggttttgaaggcgaattgaacgacttcatcaaacctgagagcaaatcactagttgcatgcagcgttaaaagagcattcattactggtgatattgatgatgctgtacatgattgtatcattacaggaaaattggatcttagtaccaacctttttggtaatgttggtctattattcaagaagactccatggtttgtacaaaagtgtggtgcactttttgtagacgcttggaaagtagtagaggagctttgtggttcactcacacttacatacaagcaaatttatgaagttgtagcatcactttgcacttctgcttttacgattgtaaactacaagccaacatttgtggttccagacaatcgtgttaaagatcttgtagacaagtgtgtgaaagttcttgtaaaagcatttgatgtttttacgcagattatcacaatagctggtattgaggccaaatgctttgtgcttggtgctaaatacctgttgttcaataatgcacttgtcaaacttgtcagtgttaaaatccttggcaagaagcaaaagggtcttgaatgtgcattctttgctactagcttggttggtgcaactgttaatgtgacacctaaaagaacagagactgccactatcagcttgaacaaggttgatgatgttgtagcaccaggagagggttatatcgtcattgttggtgatatggctttctacaagagtggtgaatattatttcatgatgtctagtcctaattttgttcttactaacaatgtttttaaagcagttaaagttccatcttatgacatcgtttatgatgttgataatgataccaaaagcaaaatgattgcaaaacttggttcatcatttgaatatgatggtgatattgatgctgctattgtaaaagtcaatgaactactcattgaatttaggcagcaaagcttgtgcttcagagcttttaaggacgacaaaagcatttttgttgaagcctattttaaaaagtataaaatgccagcatgccttgcaaaacatattggtttgtggaacatcataaagaaagattcatgtaagaggggttttcttaatctcttcaatcacttgaatgaattggaagatatcaaagaaactaatattcaggctattaaaaacattctttgccctgatcctcttcttgatctggattatggtgccatttggtacaattgcatgccaggttgctctgatccttcagttttggggagtgttcaacttttgatcggtaatggtgtgaaagtagtttgtgatggctgcaaaggttttgctaaccaactttcaaaaggttacaacaagctctgtaatgcggctcgcaatgatattgagatcggtggtataccattttccacttttaaaacacctacaaatacttttattgaaatgacagatgctatctattcagttattgaacaaggtaaggcattatcctttagagatgctgatgtgccagttgtagacaatggtaccatttctactgctgattggtctgaacccattctgcttgaacctgctgaatatgtaaaaccaaagaacaatggtaatgtcattgttattgcaggttatacattttataaagatgaggatgaacatttttatccttatggttttggtaaaattgtgcagagaatgtataataaaatgggtggtggtgacaaaactgtctcattttcagaagaagtagatgttcaagaaattgcacctgttacacgtgttaaacttgaattcgaatttgacaatgaaattgtaactggtgttcttgaacgggctattggtactagatacaaatttactggtacaacttgggaagaatttgaagagtctatttctgaagaactcgatgcaatctttgatactctagcaaaccaaggtgtcgaacttgaaggttacttcatttatgacacttgtggtggctttgatataaaaaatccagatggtattatgatctctcagtatgatatcaatattactgctgatgaaaaatcagaagttagtgcatcaagtgaagaagaagaagttgaatctgttgaagaagatcctgagaatgaaattgtagaagcatctgaaggtgctgaagggacttcttctcaagaagaggttgaaacagtagaagttgcagatattacttctacagaagaagatgttgacattgttgaagtatctgctaaagatgacccttgggctgcagctgttgatgtacaagaagctgaacaatttaatccttctctaccacctttcaagacaacgaatctcaacggaaaaattatccttaagcaaggggataataattgttggataaatgcttgttgctatcagcttcaggcctttgattttttcaacaatgaagcttgggagaaatttaagaaaggtgatgtcatggactttgtaaacctttgttatgcagcaacaacactagcaagaggtcattctggtgatgcagagtatcttcttgaacttatgctcaatgattatagcacagccaagatagtacttgcagctaagtgtggttgtggtgaaaaagaaattgttttggaaagagctgtttttaaactcaccccacttaaggagagttttaattatggtgtttgtggtgactgcatgcaagttaacacctgtagatttttaagtgttgaaggctctggtgtttttgttcatgacatattaagcaagcaaacgccagaagctatgtttgttgtcaaacctgttatgcatgcagtttacactggcacaactcaaaatggccattacatggttgatgatattgaacacggttattgtgtagatggtatgggtattaaaccacttaagaaacggtgttatacatccacattgttcattaatgccaatgtaatgactagagctgaaaaaccaaaacaagagtttaaagttgaaaaagtagaacagcaaccgatagtggaggaaaacaaatcctctattgaaaaagaggaaattcaaagtcctaaaaacgatgaccttatacttccattttacaaagctggtaaactttccttttatcagggtgctttggatgttttgatcaatttcttggaacctgatgttattgttaatgctgctaatggtgatcttaaacacatgggtggtgtcgcaagagccatcgatgttttcactggtggcaaattaacagaacgttctaaggattatcttaaaaagaacaaatctattgctcctggtaatgctgttttctttgaaaatgtcattgagcatcttagtgttttgaatgcagttggaccacgtaatggtgacagccgagttgaagccaaactttgtaatgtttacaaagcaattgcaaagtgtgaaggaaaaatattaacaccacttattagtgttggtatctttaatgttagacttgaaacatcattgcagtgcttacttaagactgtgaatgacaggggattgaatgtcttcgtatacactgaccaggagaggcaaactattgagaatttcttctcttgttctatccctgtcaatgttactgaggataatgttaaccatgaacgtgtgtctgtttcttttgacaaaacatacggtgaacagcttaagggcaccgttgtcatcaaagacaaagatgttacaaaccagttgcctagcgcttttgatgttggtcaaaaagttattaaggctattgatatagattggcaagctcattatggtttccgtgatgctgctgcttttagcgctagtagtcatgatgcttataaatttgaagttgttacacatagcaatttcattgtgcataagcagactgacaacaactgttggattaatgcaatttgtcttgcattacagagactcaagccacagtggaaatttcctggtgttagaggtctctggaatgaatttcttgagcgtaaaacacaaggttttgtacatatgttgtatcacatttctggagtaaagaaaggtgagccaggtgatgctgaattaatgctgcataaacttggtgacttgatggacaatgattgtgaaatcattgtcacacacactacagcatgtgacaagtgcgcaaaagtagaaaagtttgttggaccagtggtagcagcacctcttgcaattcatggcactgacgaaacatgtgtgcatggcgttagtgtcaatgtcaaagtcacccaaattaagggcactgttgctattacttctttgattggtcctattattggagaagtactagaagcaactggttatatttgttatagcggttctaacaggaatggtcattacacctattacgataaccgtaatggattagtggttgatgcagaaaaggcttaccattttaatagagacttattacaggtcacaacagctattgcaagtaatttcgttgtcaagaaaccacaagcagaggaaagacctaagaattgtgcttttaacaaagttgcagcatctcctaagattgtacaagaacaaaaattgttggctattgaaagtggtgctaactatgctcttactgaatttggaagatatgctgacatgttctttatggctggagataaaattcttaggttgctgcttgaagtctttaaatatttgctggttttatttatgtgtcttagaagtactaagatgcctaaagttaaagtcaaaccacctcttgcatttaaagattttggtgctaaggtcagaacgctcaattacatgagacaattgaacaaaccctctgtctggcgttacgcaaaactagttttattgttgatagcaatatataatttcttttatttgtttgtcagtataccagtagtgcataaattaacatgtaacggtgctgtacaggcatataaaaattctagttttataaagtctgcagtctgtggcaactctattttatgcaaagcctgtttggcttcttatgatgagttggctgattttcaacatctccaagttacttgggatttcaaatctgacccactatggaacagactggtacaattgtcttactttgcattcttggctgtttttggtaataactatgttaggtgttttcttatgtattttgtatctcagtacctcaacctttggctttcttattttggttatgtagagtacagttggtttttgcatgttgtcaactttgaatccatctcagctgagtttgtgatcgtagttatagtggttaaggcagttctcgcccttaaacatattgttttcgcatgctcaaacccgtcttgcaaaacgtgctctaggactgcaaggcagacacgtattcctattcaagttgttgttaatggttcaatgaagactgtttatgttcatgctaatggtactggtaaattctgcaagaaacacaatttttattgtaagaactgtgattcttatggctttgaaaacacattcatctgtgacgaaattgtacgtgatctcagtaatagtgttaaacaaactgtttacgccactgatagatctcatcaagaagtcacaaaagttgaatgttcagatggcttttacagattttatgttggtgatgaattcacttcatatgactatgatgtaaaacacaagaaatacagtagtcaagaggttctcaagagcatgctcttgcttgatgacttcattgtgtacagtccatctggttctgctcttgcaaatgttagaaatgcctgtgtttacttttcacaacttattggtaagcctattaagattgttaacagtgatttgcttgaagacctctctgtagattttaaaggggcactttttaatgctaaaaagaatgtaattaagaattctttcaatgttgatgtctcagaatgcaaaaatcttgacgaatgttacagggcttgcaatcttaatgtttcattttctacatttgaaatggctgtcaacaatgctcataggtttggtattctgattactgatcgttcttttaacaatttctggccatcaaaagttaagcctggttcatctggtgtgtcggccatggacattggtaagtgtatgacttctgatgctaagattgttaatgctaaagttttaactcaacgtggtaaaagtgttgtttggcttagccaggattttgctgcacttagctcaactgctcagaaagttttggttaaaacttttgtagaagaaggtgtcaacttttcactcacatttaatgctgtaggttcagatgatgatcttccttatgaaagattcactgaatctgtgtctccaaaaagtggttcaggctttttcgatgtaattacacagcttaaacaaattgtgattttggtttttgtttttatctttatttgtggtttgtgctctgtttacagtgttgctacacagtcctacattgaatctgctgaaggctatgactacatggttattaagaatggaattgttcaaccttttgacgataccatttcatgtgttcataacacttataaaggattcggtgactggtttaaagctaagtatggttttatccctacttttggtaaatcatgtccaattgttgtaggaactgtttttgatcttgaaaatatgagaccaattcctgacgtgcctgcatatgtttctattgtgggtagatctcttgttttcgctattaatgctgcttttggtgttactaatatgtgctatgatcatactggcaatgcagttagtaaggactcttactttgatacttgtgtgtttaatactgcgtgcaccactcttacaggtcttggtggtacaattgtatattgtgcaaagcaaggtttagttgaaggtgctaagctctatagtgatcttatgccagactattattatgagcatgctagtggtaacatggttaaattgccagcaattattagaggacttggtctacgttttgtgaaaacacaggctacaacttattgtagagtgggagagtgcattgatagtaaagctggtttttgctttggtggcgataactggtttgtctacgacaatgagtttggcaatggatacatctgtggtaattctgtgctaggattctttaagaatgtcttcaaactctttaactctaacatgtctgtggtagctacatctggtgcgatgcttgttaacattattattgcatgcttagctattgcaatgtgttatggtgttcttaagtttaagaagatttttggtgattgtactttcctcattgttatgatcattgtcacccttgttgtgaacaatgtgtcttattttgtcactcaaaacacgttctttatgatcatctacgccattgtttactattttataacaagaaaacttgcatacccaggcattcttgatgctgggtttattattgcttatattaatatggctccatggtacgtgattaccgcatatatcctagttttcctctatgactcactcccttcactgtttaaacttaaagtttcaacaaatctttttgaaggtgataaatttgtgggtaactttgaatctgctgctatgggtacttttgttattgacatgcgttcatatgaaactattgttaattctacttctattgctagaattaaatcatatgctaacagcttcaataaatataagtactacacaggttcaatgggagaagctgactacagaatggcttgctatgctcatcttggtaaagctcttatggactattctgttaatagaacagacatgctttacacacctcctactgttagtgttaattctacacttcagtcaggtttgcggaaaatggcacagcctagtggtcttgtagagccttgcattgtaagagtttcctatggtaacaatgtgcttaatggtttatggttaggagatgaagtcatttgccctagacatgttattgctagtgataccacacgtgttatcaactatgaaaatgaaatgtctagtgtgagacttcacaacttttcagtttctaagaataatgtgtttttgggtgttgtgtctgccagatataagggtgtgaatcttgtacttaaagtcaaccaggttaatcctaacacaccagaacataaatttaagtctattaaagctggtgaaagttttaacattcttgcttgttatgaaggatgtcctggcagtgtttatggtgtcaacatgagaagtcaaggtaccattaaaggatcttttatagctggtacttgtggatcagtaggttatgtgttagaaaatggaattctctattttgtatacatgcatcacttagaacttggaaatggctcgcatgttggttccaattttgaaggagaaatgtacggtggttatgaagatcaacctagcatgcaattggaaggtactaatgtcatgtcatcagataatgtggttgcattcctatatgctgcacttatcaatggtgaaaggtggtttgttacaaacacatcgatgtcattagaatcatacaatacatgggccaaaactaacagtttcacagaactttcttcaactgatgcttttagcatgttggctgcaaaaactggtcaaagtgttgagaaattactagatagcatcgtaagactcaacaagggttttggaggtcgtactatactttcttatggctcattgtgtgacgagttcactccaactgaagtcataaggcaaatgtatggtgtaaatcttcaggctggtaaagtaaaatctttcttctaccctattatgactgcaatgacaattctctttgccttttggcttgaattctttatgtacacacccttcacttggattaatccaacttttgttagcattgtattggctgttacaactttgatctcgacggtttttgtctctggcatcaaacataagatgttgttctttatgtcttttgtccttcctagtgttatccttgtgacagcacacaatttgttctgggacttttcttactatgaaagtcttcagtcaattgttgagaatactaacactatgtttttgcctgttgacatgcaaggtgtcatgctcacagtgttttgctttattgtctttgttacatatagtgttagattcttcacttgcaaacaatcatggttctcacttgctgtgacaactattcttgtgatctttaacatggttaaaatctttggaacatctgatgaaccatggactgaaaaccaaattgctttctgctttgtgaacatgcttactatgattgtcagtcttactacaaaggattggatggttgtcattgcatcatacagaattgcatattatattgttgtatgtgtaatgccatctgcttttgtatctgactttgggtttatgaagtgtattagcattgtttacatggcgtgcggttatttgttttgttgctattatggcattctttattgggttaacagatttacatgcatgacttgtggtgtttatcaattcactgtgtctgcagctgaacttaaatacatgaccgctaacaacctttctgcacctaagaacgcatatgacgctatgattcttagtgctaaattgattggtgttggaggtaagagaaacatcaaaatttcaactgtacagtcaaaacttacagagatgaaatgtaccaatgttgtcttgcttggtcttttatctaaaatgcatgtcgagtctaactcaaaagagtggaactattgtgttggactacacaatgagataaacctttgtgacgatcctgaaatcgttcttgagaaactgttagctcttattgcattcttcttgtccaaacataacacttgtgaccttagcgaacttattgaatcatactttgagaacaccaccatactccagagtgtggcttcagcttatgctgcattgcctagctggattgcacttgaaaaagctcgcgctgatcttgaagaggctaagaaaaatgatgttagccctcaaattttgaagcagcttactaaagcatttaacattgccaagagtgattttgagcgcgaagcatcagtgcaaaagaaactcgacaaaatggctgagcaggctgcagctagtatgtataaagaagcacgagctgtggacagaaagtcaaagattgtttctgctatgcatagcctactttttggtatgcttaagaaacttgatatgtccagtgtcaacactattattgaccaggctcgtaatggtgttctacctttaagtatcattccagctgcatcagctacaagacttgttgttattacacctagccttgaagtgttttccaagattaggcaagaaaacaatgttcattatgctggtgctatttggactattgttgaagttaaagatgctaatggttcacatgtacatcttaaggaagtcaccgctgctaatgaattaaaccttacttggccattgagcattacttgtgagagaaccacaaagcttcagaacaatgaaattatgccaggtaaacttaaagaaagagctgtcagagcgtcagcaactcttgatggtgaagctttcggcagtggaaaggctcttatggcatctgaaagtggaaaaagctttatgtatgcatttatagcctcagacaacaatcttaagtatgttaagtgggagagcaataatgatattatacctattgaacttgaagctccattgcgtttctatgttgacggcgctaatggtcctgaagtcaagtatttgtattttgtcaagaatttaaacactcttagacgtggtgccgttcttggttatatcggtgcaacagttcgtctgcaagctggtaaacccactgaacatccatctaacagtagtttattgacattgtgtgctttttcacctgatcctgctaaagcatatgttgatgctgttaagagaggcatgcaaccagttaataactgtgtaaaaatgctctcaaatggtgctggtaatggtatggctgttacaaacggtgtcgaagctaacacacaacaggactcttatggtggtgcttcagtttgtatttattgcagatgccatgttgaacatcctgctattgatggattatgccgctacaaaggtaagttcgtgcaaataccaactggcacacaagatccaattcggttctgtattgaaaatgaagtttgtgttgtctgtggttgttggcttaacaatggttgcatgtgcgatcgtacttctatgcagagttttactgttgatcaaagttatttaaacgagtgcggggttctagtgcagctcgactagaaccctgcaatggtactgatccagaccatgttagtagagcttttgacatctacaacaaagatgttgcgtgtattggtaaattccttaagacgaattgttcaagatttaggaatttggacaaacatgatgcctactacattgtcaaacgttgtacaaagaccgttatggaccatgagcaagtctgttataacgatcttaaagattctggtgctgttgctgagcatgacttcttcacatataaagagggtagatgtgagttcggtaatgttgcacgtaggaatcttacaaagtacacaatgatggatctttgttacgctatcagaaattttgatgaaaagaactgtgaagttctcaaagaaatactcgtgacagtaggtgcttgcactgaagaattctttgaaaataaagattggtttgatccagttgaaaatgaagccatacatgaagtttatgcaaaacttggacccattgtagccaatgctatgcttaaatgtgttgctttttgcgatgcgatagtggaaaaaggctatataggtgttataacacttgacaaccaagatcttaatggcaatttctacgatttcggcgatttcgtgaagactgctccgggttttggttgcgcttgtgttacatcatattattcttatatgatgcctttaatggggatgacttcatgcttagagtctgaaaactttgtgaaaagtgacatctatggttctgattataagcagtatgatttactagcttatgattttaccgaacataaggagtaccttttccaaaaatactttaagtactgggatcgcacatatcacccaaattgttctgattgtactagtgacgagtgtattattcattgtgctaattttaacacattgttttctatgacaataccaatgacagcttttggaccacttgtccgtaaagttcatattgatggtgtaccagtagttgttactgcaggttaccatttcaaacaacttggtatagtatggaatcttgatgtaaaattagacacaatgaagttgagcatgactgatcttcttagatttgtcacagatccaacacttcttgtagcatcaagccctgcacttttagaccagcgtactgtctgtttctccattgcagctttgagtactggtattacatatcagacagtaaaaccaggtcactttaacaaggatttctacgatttcataacagagcgtggattctttgaagagggatctgagttaacattaaaacattttttctttgcacagggtggtgaagctgctatgacagacttcaattattatcgctacaatagagtcacagtacttgatatttgccaagctcaatttgtttacaaaatagttggcaagtattttgaatgttatgacggtgggtgcattaatgctcgtgaagttgttgttacaaactatgacaagagtgctggctatcctttgaacaaatttggtaaagctagactttactacgaaactctttcatatgaagagcaggatgcactttttgctttaacaaagagaaatgttttacccacaatgactcaaatgaatttgaaatacgctatttctggtaaggcaagagctcgtacagtaggaggagtttcacttctttctaccatgactacgagacaatatcatcagaagcatttgaagtcaattgctgcaacacgcaatgctactgtggtcattggttcaaccaagttttatggtggttgggacaatatgcttaaaaatttaatgcgtgatgttgataatggttgtttgatgggatgggactatcctaagtgtgaccgtgctttacctaatatgattagaatggcttctgccatgatattaggttctaagcatgttggttgttgtacacataatgataggttctaccgcctctccaatgagttagctcaagtactcacagaagttgtgcattgcacaggtggtttttattttaaacctggtggtacaactagcggtgatggtactacagcatatgctaactctgcttttaacatctttcaagctgtttctgctaatgttaataagcttttgggggttgattcaaacgcttgtaacaacgttacagtaaaatccatacaacgtaaaatttacgataattgttatcgtagtagcagcattgatgaagaatttgttgttgagtactttagttatttgagaaaacacttttctatgatgattttatctgatgatggagttgtgtgctacaacaaagattatgcggatttaggttatgtagctgacattaatgcttttaaagcaacactttattaccagaataacgtctttatgtccacttctaagtgttgggtagaaccagatcttagtgttggaccacatgaattttgttcacagcatacattgcagattgttgggcctgatggagactactatcttccctatccagacccgtccagaattttgtcagctggtgtgtttgttgatgacatagttaaaacagacaatgttattatgttagaacgttacgtgtcattggctattgacgcatacccgctcacaaaacaccctaagcctgcttatcaaaaagtgttttacactctactagattgggttaaacatctacagaaaaatttgaatgcaggtgttcttgattcgttttcagtgacaatgttagaggaaggtcaagataagttctggagtgaagagttttacgctagcctctatgaaaagtccactgtcttgcaagctgcaggcatgtgtgtagtatgtggttcgcaaactgtacttcgttgtggagactgtcttaggagaccacttttatgcacgaaatgtgcttacgaccatgttatgggaacaaagcataaattcattatgtctatcacaccatatgtgtgtagttttaatggttgtaatgtcaatgatgttacaaagttgtttttaggtggtcttagttattattgtatgaaccacaaaccacagttgtcattcccactctgtgctaatggcaacgtttttggtctatataaaagtagtgcagtcggctcagaggctgttgaagatttcaacaaacttgcagtttctgactggactaatgtagaagactacaaacttgctaacaatgtcaaggaatctctgaaaattttcgctgctgaaactgtgaaagctaaggaggagtctgttaaatctgaatatgcttatgctgtattaaaggaggttatcggccctaaggaaattgtactccaatgggaagcttctaagactaagcctccacttaacagaaattcagttttcacgtgttttcagataagtaaggatactaaaattcaattaggtgaatttgtgtttgagcaatctgagtacggtagtgattctgtttattacaagagcacgagtacttacaaattgacaccaggtatgatttttgtgttgacttctcataatgtgagtcctcttaaagctccaattttagtcaaccaagaaaagtacaataccatatctaagctctatcctgtctttaatatagcggaggcctataatacactggttccttactaccaaatgataggtaagcaaaaatttacaactatccaaggtcctcctggtagcggtaaatctcattgtgttataggtttgggtttgtattaccctcaggcgagaatagtctacactgcatgttctcatgcggctgtagacgctttatgtgaaaaagcagccaaaaacttcaatgttgatagatgttcaaggataatacctcaaagaatcagagttgattgttacacaggctttaagcctaataacaccaatgcgcagtacttgttttgtactgttaatgctctaccagaagcaagttgtgacattgttgtagttgatgaggtctctatgtgtactaattatgatcttagtgtcataaatagccgactgagttacaaacatattgtttatgttggagacccacagcagctaccagctcctagaactttgattaataagggtgtacttcaaccgcaggattacaatgttgtaaccaaaagaatgtgcacactaggacctgatgtctttttgcataaatgttacaggtgcccagctgaaattgttaagacagtctctgcacttgtttatgaaaataaatttgtacctgtcaacccagaatcaaagcagtgcttcaaaatgtttgtaaaaggtcaggttcagattgagtctaactcttctataaacaacaagcaactagaggttgtcaaggcctttttagcacataatccaaaatggcgtaaagctgttttcatctcaccctataatagtcaaaattatgttgctcggcgtcttcttggtttgcaaacgcaaactgtggattccgctcagggtagtgagtatgattacgtcatctacacacagacctccgatacacagcatgctactaatgttaacagatttaatgttgccattacgagagcaaaggttggtatactttgtatcatgtgtgatagaactatgtatgagaatcttgatttctatgaactcaaagattcaaagattggtttacaagcaaaacctgaaacttgtggtttatttaaagattgttcgaagagcgaacaatacataccacctgcttatgcaacgacatatatgagcttatctgataattttaagacaagtgatggtttagctgttaacatcggtacaaaagatgttaaatatgctaatgtcatctcatatatgggattcaggtttgaagccaacataccaggctatcacacactattctgcacgcgagattttgctatgcgtaatgttagagcatggcttgggtttgacgttgaaggtgcacatgtctgtggtgataatgttggaactaatgtaccattacagctgggtttctcaaacggtgtggattttgtagtgcaaactgaaggatgtgttattactgaaaaaggtaatagcattgaggttgtaaaagcacgagcaccaccaggtgagcaatttgcacacttgattccgcttatgagaaagggtcaaccttggcacattgttagacgccgtatagtgcagatggtctgtgactattttgatggcttatcagacattctgatctttgtgctttgggctggtggtcttgaacttacaactatgagatactttgttaaaattggaagaccacaaaaatgtgaatgcggcaaaagtgcaacttgttatagtagctctcaatctgtttatgcttgcttcaagcatgcattaggatgtgattatttatataacccttactgcattgacatacagcaatggggttacacaggatctttgagcatgaatcatcatgaagtttgcaacattcatagaaatgagcatgtagctagtggtgatgctatcatgactagatgtctcgctatacatgactgttttgtcaaacgtgttgattggtcaattgtgtacccttttattgacaatgaagaaaagatcaataaagctggtcgcatagtgcagtcacatgtcatgaaagctgctctgaagatttttaatcctgctgcaattcacgatgtgggtaatccaaaaggcatccgttgtgctacaacaccaataccatggttttgttatgatcgtgatcctattaataacaatgttagatgtctggattatgactatatggtacatggtcaaatgaatggtcttatgttattttggaactgtaatgtagacatgtacccagagttttcaattgtttgtagatttgatactcgcactcgctctaaattgtctttagaaggttgtaatggtggtgcattgtatgttaataaccatgctttccacacaccagcttatgatagaagagcttttgctaagcttaaacctatgccattcttttactatgatgatagtaattgtgaacttgttgatgggcaacctaattatgtaccacttaagtcaaatgtttgcataacaaaatgcaacattggtggtgctgtctgcaagaagcatgctgctctttacagagcgtatgttgaggattacaacatttttatgcaggctggttttacaatatggtgtcctcaaaactttgacacctatatgctttggcatggttttgttaatagcaaagcacttcagagtctagaaaatgtggcttttaatgtcgttaagaaaggtgccttcaccggtttaaaaggtgacttaccaactgctgttattgctgacaaaataatggtaagagatggacctactgacaaatgtatttttacaaataagactagtttacctacaaatgtagcttttgagttatatgcaaaacgcaaacttggactcacacctccattaacaatacttaggaatttaggtgttgtcgcaacatataagtttgtgttgtgggattatgaagctgaacgtcctttctcaaatttcactaagcaagtgtgttcctacactgatcttgatagtgaagttgtaacatgttttgataatagtattgctggttcttttgagcgttttactactacaagagatgcagtgcttatttctaataacgctgtgaaagggcttagtgccattaaattacaatatggccttttgaatgatctacctgtaagtactgttggaaataaacctgtcacatggtatatctatgtgcgcaagaatggtgagtacgtcgaacaaatcgatagttactatacacagggacgtacttttgaaaccttcaaacctcgtagtacaatggaagaagattttcttagtatggatactacactcttcatccaaaagtatggtcttgaggattatggttttgaaGCAgttgtatttggagatgtctctaaaactaccattggtggtatgGCActtcttatatcgcaagtgcgccttgcaaaaatgggtttgttttccgttcaagaatttatgaataattctgacagtacactgaaaagttgttgtattacatatgctgatgatccatcttctaagaatgtgtgcacttatatggacatactcttggacgattttgtgactatcattaagagcttagatcttaatgttgtgtccaaagttgtggatgtcattgtagattgtaaggcatggagatggatgttgtggtgtgagaattcacatattaaaaccttctatccacaactccaatctgctgaatggaatcccggctatagcatgcctacactgtacaaaatccagcgtatgtgtctcgaacggtgtaatctctacaattatggtgcacaagtgaaattacctgatggcattactactaatgtcgttaagtatactcagttgtgtcaataccttaacactactacattgtgtgtaccacacaaaatgcgtgtattgcatttaggagctgctggtgcatctggtgttgctcctggtagtactgtattaagaagatggttaccagatgatgccatattggttgataatgatttgagagattacgtttccgacgcagacttcagtgttacaggtgattgtactagtctttacatcgaagacaagtttgatttgctcgtctctgatttatatgatggctccacaaaatcaattgacggtgaaaacacgtcgaaagatggtttctttacttatattaatggtttcattaaagagaaactgtcacttggtggatctgttgccattaaaatcacggaatttagttggaataaagatttatatgaattgattcaaagatttgagtattggactgtgttttgtacaagtgttaacacgtcatcatcagaaggctttctgattggtattaactacttaggaccatactgtgacaaagcaatagtagatggaaatataatgcatgccaattatatattttggagaaactctacaattatggctctatcacataactcagtcctagacactcctaaattcaagtgtcgttgtaacaacgcacttattgttaatttaaaagaaaaagaattgaatgaaatggtcattggattactaaggaagggtaagttgctcattagaaataatggtaagttactaaactttggtaaccacttcgttaacacaccatgaaaaaactatttgtggttttggtcgtaatgccattgatttatggagacaattttccttgttctaaattgactaatagaactataggcaaccagtggaatctcattgaaaccttccttctaaactatagtagtaggttaccacctaattcagatgtggtgttaggtgattattttcctactgtacaaccttggtttaattgcattcgcaatgatagtaatgacctttatgttacactggaaaatcttaaagcattgtattgggattatgctacagaaaatatcacttggaatcacagacaacggttaaacgtagtcgttaatggatacccatactccatcacagttacaacaacccgcaattttaattctgctgaaggtgctattatatgcatttgtaagggctcaccacctactaccaccacagaatctagtttgacttgcaattggggtagtgagtgcaggttaaaccataagttccctatatgtccttctaattcagaggcaaattgtggtaatatgctgtatggcctacaatggtttgcagatgaggttgttgcttatttacatggtgctagttaccgtattagttttgaaaatcaatggtctggcactgtcacatttggtgatatgcgtgcgacaacattagaagtcgctggcacgcttgtagacctttggtggtttaatcctgtttatgatgtcagttattatagggttaataataaaaatggtactaccgtagtttccaattgcactgatcaatgtgctagttatgtggctaatgtttttactacacagccaggaggttttataccatcagattttagttttaataattggttccttctaactaatagctccacgttggttagtggtaaattagttaccaaacagccgttattagttaattgcttatggccagtccctagctttgaagaagcagcttctacattttgttttgagggtgctggctttgatcaatgtaatggtgctgttttaaataatactgtagacgtcattaggttcaaccttaattttactacaaatgtacaatcaggtaagggtgccacagtgttttcattgaacacaacgggtggtgtcactcttgaaatttcatgttatacagtgagtgactcgagctttttcagttacggtgaaattccgttcggcgtaactgatggaccacggtactgttacgtacactataatggcacagctcttaagtatttaggaacattaccacctagtgtcaaggagattgctattagtaagtggggccatttttatattaatggttacaatttctttagcacatttcctattgattgtatatcttttaatttgaccactggtgatagtgacgttttctggacaatagcttacacatcgtacactgaagcattagtacaagttgaaaacacagctattacaaaggtgacgtattgtaatagtcacgttaataacattaaatgctctcaaattactgctaatttgaataatggattttatcctgtttcttcaagtgaagttggtcttgtcaataagagtgttgtgttactacctagcttttacacacataccattgttaacataactattggtcttggtatgaagcgtagtggttatggtcaacccatagcctcaacattaagtaacatcacactaccaatgcaggatcacaacaccgatgtgtactgtattcgttctgaccaattttcagtttatgttcattctacttgcaaaagtgctttatgggacaatatttttaagcgaaactgcacggacgttttagatgccacagctgttataaaaactggtacttgtcctttctcatttgataaattgaacaattacttaacttttaacaagttctgtttgtcgttgagtcctgttggtgctaattgtaagtttgatgtagctgcccgtacaagaaccaatgagcaggttgttagaagtttgtatgtaatatatgaagaaggagacaacatagtgggtgtaccgtctgataatagtggtgtgcacgatttgtcagtgctacacctagattcctgcacagattacaatatatatggtagaactggtgttggtattattagacaaactaacaggacgctacttagtggcttatattacacatcactatcaggtgatttgttaggttttaaaaatgttagtgatggtgtcatctactctgtaacgccatgtgatgtaagcgcacaagcagctgttattgatggtaccatagttggggctatcacttccattaacagtgaactgttaggtctaacacattggacaacaacacctaatttttattactactctatatataattacacaaatgataggactcgtggcactgcaattgacagtaatgatgttgattgtgaacctgtcataacctattctaacataggtgtttgtaaaaatggtgcttttgtttttattaacgtcacacattctgatggagacgtgcaaccaattagcactggtaatgtcacgatacctacaaactttaccatatccgtgcaagtcgaatatattcaggtttacactacaccagtgtcaatagactgttcaagatatgtttgtaatggtaaccctaggtgtaacaaattgttaacacaatacgtttctgcatgtcaaactattgagcaagcacttgcaatgggtgccagacttgaaaacatggaggttgattccatgttgtttgtttctgaaaatgcccttaaattggcatctgttgaagcattcaatagttcagaaactttagaccctatttacaaagaatggcctaatataggtggttcttggctagaaggtctaaaatacatacttccgtcccataatagcaaacgtaagtatcgttcagctatagaggacttgctttttgataaggttgtaacatctggtttaggtacagttgatgaagattataaacgttgtacaggtggttatgacatagctgacttagtatgtgctcaatactataatggcatcatggtgctacctggtgtggctaatgctgacaaaatgactatgtacacagcatcccttgcaggtggtataacattaggtgcacttggtggaggcgccgtggctataccttttgcagtagcagttcaggctagacttaattatgttgctctacaaactgatgtattgaacaaaaaccagcagattctggctagtgctttcaatcaagctattggtaacattacacagtcatttggtaaggttaatgatgctatacatcaaacatcacgaggtcttgctactgttgctaaagcattggcaaaagtgcaagatgttgtcaacatacaagggcaagctttaagccacctaacagtacaattgcaaaataatttccaagccattagtagttctattagtgacatttataataggcttgacgaattgagtgctgatgcacaagttgacaggctgatcacaggaagacttacagcacttaatgcatttgtgtctcagactctaaccagacaagcggaggttagggctagtagacaacttgccaaagacaaggttaatgaatgcgttaggtctcagtctcagagattcggattctgtggtaatggtacacatttgttttcactcgcaaatgcagcaccaaatggcatgattttctttcacacagtgctattaccaacggcttatgaaactgtgactgcttggccaggtatttgtgcttcagatggtgatcgcacttttggacttgtcgttaaagatgtccagttgactttgtttcgtaatctagatgacaagttctatttgacccccagaactatgtatcagcctagagttgcaactagttctgactttgttcaaattgaagggtgcgatgtgctgtttgttaatgcaactgtaagtgatttgcctagtattatacctgattatattgatattaatcagactgttcaagacatattagaaaattttagaccaaattggactgtacctgagttgacatttgacatttttaacgcaacctatttaaacctgactggtgaaattgatgacttagaatttaggtcagaaaagctacataacaccactgtagaacttgccattctcattgacaacattaacaatacattagtcaatcttgaatggctcaatagaattgaaacctatgtaaaatggccttggtatgtgtggctactaataggcttagtagtaatattttgcataccattactgctattttgctgttgtagtacaggttgctgtggatgcataggttgtttaggaagttgttgtcactctatatgtagtagaagacaatttgaaaattacgaaccaattgaaaaagtgcacgtccattaaatttaaaatgttaattctatcatctgctataatagcagttgtttctgctagagaattttgttaaggatgatgaataaagtctttaagaactaaacttacgagtcattacaggtcctgtatggacattgtcaaatccatttacacatccgtagatgctgtacttgacgaacttgattgtgcatactttgctgtaactcttaaagtagaatttaagactggtaaattacttgtgtgtataggttttggtgacacacttcttgctgctaaggataaagcatatgctaagcttggtctctccattattgaagaagtcaatagtcatatagttgtttaatatcattaaacacacaaaacccaaagcattaagtgttacaaaacaattaaagagagattatagaaaaactgtcattctaaattccatgcgaaaatgattggtggactttttcttagtactctgagttttgtaattgttagtaaccattctattgttaataacacagcaaatgtgcatcatatacaacaagaacgtgttatagtacaacagcatcaggttgttagtgctagaacacaaaactattacccagagttcagcatcgctgtactctttgtatcttttctagctttgtaccgtagtacaaactttaagacgtgtgtcggcatcttaatgtttaagattttatcaatgacacttttaggacctatgcttatagcatatggttactacattgatggcattgttacaacaactgtcttatctttaagatttgtctacttagcatacttttggtatgttaatagtaggtttgaatttattttatacaatacaacgacactcatgtttgtacatggcagagctgcaccgtttatgagaagttctcacagctctatttatgtcacattgtatggtggcataaattatatgtttgtgaatgacctcacgttgcattttgtagaccctatgcttgtaagcatagcaatacgtggcttagctcatgctgatctaactgtagttagagcagttgaacttctcaatggtgattttatttatgtattttcacaggagcccgtagtcggtgtttacaatgcagccttttctcaggcggttctaaacgaaattgacttaaaagaagaagaagaagaccatacctatgacgtttcctagggcattgactgtcatagatgacaatggaatggtcattaacatcattttctggttcctgttgataattatattgatattactttcaatagcattgctaaatataattaagctatgcatggtgtgttgcaatttaggaaggacagttattattgttccagcgcaacatgcttacgatgcctataagaattttatgcgaattaaagcatacaaccccgatggagcactccttgcttgaactaaacaaaatgaagattttgttaatattagcgtgtgtgattgcatgcgcatgtggagaacgctattgtgctatgaaatccgatacagatttgtcatgtcgcaatagtacagcgtctgattgtgagtcatgcttcaacggaggcgatcttatttggcatcttgcaaactggaacttcagctggtctataatattgatcgtttttataactgtgctacaatatggaagacctcaattcagctggttcgtgtatggcattaaaatgcttataatgtggctattatggcccgttgttttggctcttacgatttttaatgcatactcggaataccaagtgtccagatatgtaatgttcggctttagtattgcaggtgcaattgttacatttgtactctggattatgtattttgtaagatccattcagttgtacagaaggactaagtcttggtggtctttcaaccctgaaactaaagcaattctttgcgttagtgcattaggaagaagctatgtgcttcctctcgaaggtgtgccaactggtgtcactctaactttgctttcagggaatttgtacgctgaagggttcaaaattgcaggtggtatgaacatcgacaatttaccaaaatacgtaatggttgcattacctagcaggactattgtctacacacttgttggcaagaagttgaaagcaagtagtgcgactggatgggcttactatgtaaaatctaaagctggtgattactcaacagaggcaagaactgataatttgagtgagcaagaaaaattattacatatggtataactaaacttctaaatggccaaccagggacaacgtgtcagttggggagatgaatctaccaaaacacgtggtcgttccaattcccgtggtcggaagaataataacatacctctttcattcttcaaccccataaccctccaacaaggttcaaaattttggaacttatgtccgagagactttgtacccaaaggaataggtaacagggatcaacagattggttattggaatagacaaactcgctatcgcatggtgaagggccaacgtaaagagcttcctgaaaggtggttcttctactacttaggtactggacctcatgcagatgccaaatttaaagataaattagatggagttgtctgggttgccaaggatggtgccatgaacaaaccaaccacgcttggtagtcgtggtgctaataatgaatccaaagctttgaaattcgatggtaaagtgccaggcgaatttcaacttgaagttaatcaatcaagagacaattcaaggtcacgctctcaatctagatctcggtctagaaatagatctcaatctagaggcaggcaacaattcaataacaagaaggatgacagtgtagaacaagctgttcttgccgcacttaaaaagttaggtgttgacacagaaaaacaacagcaacgctctcgttctaaatctaaagaacgtagtaactctaagacaagagatactacacctaagaatgaaaacaaacacacctggaagagaactgcaggtaaaggtgatgtgacaagattttatggagctagaagcagttcagccaattttggtgacactgacctcgttgccaatgggagcagtgccaagcattacccacaactggctgaatgtgttccatctgtgtctagcattctgtttggaagctattggacttcaaaggaagatggcgaccagatagaagtcacgttcacacacaaataccacttgccaaaggatgatcctaagactggacaattccttcagcagattaatgcctatgctcgtccatcagaagtggcaaaagaacagagaaaaagaaaatctcgttctaaatctgcagaaaggtcagagcaagatgtggtacctgatgcattaatagaaaattatacagatgtgtttgatgacacacaggttgagataattgatgaggtaacgaactaaacgagatgctcgtcttcctccatgctgtatttattacagttttaatcttactactaattggtagactccaattattagaaagactattacttaatcactctttcaatcttaaaactgtcaatgactttaatatcttatataggagtttagcagaaaccagattactaaaagtggtgcttcgagtaatctttctagtcttactaggattttgctgctacagattgttagtcacattaatgtaaggcaacccgatgtctaaaa

**WT-FIPV**

AGCGTTGATTATTTCACTCAGTTTGGCAATCACTCCTAGGAACGGGGTTGAGAGAACGGCGCACCAGGGTTCCGTCCCTGTTTGGTAAGTCGTCTAGTATTAGCTGCGGCGGTTCCGCCCGTCGTAGTTGGGTAGACCGGGTTCCGTCCTGTGATCTCCCTCGCCGGCCGCCAGGAGAATGAGTTCCAAACAATTTAAGATCCTCGTTAATGAGGACTACCAAGTCAACGTGCCTAGCCTTCCTTTCCGTGACGTGCTGCAGGACATTAAGTACTGCTACCGGAACGGTTTTGATGGCTATGTCTTCGTGCCTGAATACCGTCGTGACCTAGTTGATTGCAATCGTAAGGATCACTACGTCATTGGTGTTTTGGGTAACGGAATAAGTGATCTTAAACCTGTCCTCCTTACCGAACCTTCCGTCATGTTGCAGGGTTTTATTGTTAGAGCTGACTGCAATGGCGTTCTTGAGGACTTTGACCTTAAAATTGCCCGTACTGGAAATGGTGCCATATATGTAGACCAATACATGTGTGGTGCCGATGGAAAGCCAGTCATTGAAGGTGAGTTTAAGGACTATTTCGGTGATGAGGACGTTATTATCTATGAAGGAGAGGAGTATCATTGTGCCTGGTTAACAGTGCGCGATGAAAAACCTTTGTGTCAACAAACTCTCCTTACAATTAGGGAAATCCAATACAATCTGGACGTCCCACATAAGTTGCCAAATTGTGCTATCAGAGAGGTAGCACCACCAGTCAAGAAGAACTCCAAGGTTGTTCTTTCTGAAGAGTATAAAAAGCTCTATGACATCTTCGGTTCACCATTTATGGGCAATGGGGACAGTCTTAACAAGTGTTTCGACAGCCTCCACTTCATCGCTGCTACTCTTAAGTGTCCTTGTGGCTCTGAAAGTAGTGGTGTTGGTGACTGGACTGGTTTTAAGACTGCTTGTTGTGGTCTTCATGGCAAAGTCAGAGGTGTCACACTAGGTGCCGTTAAACCTGGTGATGCTGTTATCACTAGCATGAGCGCTGGCAAGGGTGTAAAATTCTTTGCTAATAGTGTGCTCCAATATGCTGGTGATGTTGAAAATGTCTCTGTTTGGAAAGTGATTAAAACTTTCACTGTTAACGAGACTGTTTGCACCGCCGATTTTGAAGGCGAATTGAACGACTTCATCAAACCAGAGAGTAAGTCACTGGTTTCGTGCTCTATTAAGAGGGCTTTCATCACTGGTGAAGTTGATGATGCAGTCCATGATTGTATTATCACTGGAAAGTTGGACCTTAGTACTAATCTTTTTGGTAGTGCTAGCCTACTTTTTGAGAAAACACCCTGGTTTGTGCAAAAGTGTGGTGCACTTTTTGCTGATGCTTGGAAAGTTATTGAAGAACTGCTTTGTTCTCTTAAACTTACGTACAAGCAAATCTATGATGTAGTGGCATCACTTTGCACTTCTGCCTTCACTATTATGGACTACAAACCTGTGTTTGTAGTATCGGCTAATAGTGTTAAGGACCTCGTTGACAAGTGTGTCAAAATTCTTGTGAAAGCCTTTGATGTTTTCACACAGACAATTACAATTGCGGGTGTAGAGGCTAAGTGCTTTGTGCTTGGTTCTAAATACTTGCTTTTTAACAACGCACTTGTCAAGCTTGTCAGTGTTAAAATACTTGGCAAGAGACAGAAAGGTCTTGATAGTGCATTCTTTGCTACTAACTTAATTGGTGCGACTGTTAATGTGACACCGCAGAGGACTGAGGCTGCTAACATTAGCCTGAATAAGGTTGATGATGTTGCTACACCAGGTGAAGGTCACATTGTCATCATTGGCGACATGGCTTTTTACAAGAGTGAAGAATACTATTTTATGATGGCAAGTCCAGATTCAGTCCTTGTTAATAATGTTTTTAAAGCAGCTCGAGTGCCGTCTTATAACATCACTTATGATGTGGATGATGATACCAAGAGTAAGATGTTAGTTAAAATTGGAACATCATTTGATTTTGATGGTGATCTTGACGCTGCTATTGCTAAAGTTAATGACTTACTTATAGAGTTTAGACAAGACAAGTTGTGTTTCAGAGCACTTAAGGATTGTGAAAACATCTTAGTTGAGGCATACCTTAAGAAATACAAGATGCCAGTTTGTCTCAAAAACCATGTTGGTTTGTGGGACATCATAAGGCGAGATTCTGACAAGAAGGGTTTTCTTGACACTTTCAATCATCTTAATGAATTGGAGGATGTCAAGGACACAAAGATTCAGTCTATCAAAGATATTCTTTGTCCTGACCTTCTGCTTGAATTGGACTTTGGCGCAATCTGGTATAGATGTATGCCTGTCTGCTCTGATGTGTCTATCCTAGGTAATGTTAAAATTATGCTAGGCAATGGTGTTAAAGTGCTTTGCGATGGCTGTAACAGTTTTGCCAAACGTTTAACAGTTAGTTACAGCAAACTGTGTGATACAGCTCGTAAAGATGTTGAAATTGGTGGCTTACCGTTTTCAACTTTTAAAACACCAGCTAGCAGTTTTATCGACATGAAAGATGCCATCTATTCTGTAGTTGAATATGGTGAAGCATTTTCTTTCAAGACTGCAATTGTACCTGTAATTAATAGCGGCACCGTCACAACCGACGATTGGTCAGACCCCATATTGTTAGAACCTGCAGACTATGTTGAACCTAAAGATAACGGCGATATAATTGTCATTGCTGGATACACCTTCTACAAGGATGAAGATGATCATTTTTATCCATATGGTTCTGGCATGGTTGTGCAGAAGATGTACAACAAGATGGGTGGTGGTGACAAATCAGTTTCATTCTCAGACGATGTTAATGTTAAAGAGATCGAACCTATTACACGTGTCAGGCTAGAATTTGAGTTTGACAATGAGGTTGTAACACAGGTTCTTGAAAAAGTCATTGGTACCAAATATAAGTTTATAGGTACCACCTGGGAAGAGTTTGAAGATTCTATCTCTGAGAAACTTGACAATATCTTTGACAAGCTTGCTGAACAGGGTGTAGAACTTGAAGGTTACTTCATTTATGACACATGTGGTGGATTTGACATTAACAATCCAGATGGTGTTATGATCTCACAATATGATCTTAACACAGATGTTGAAGACAAAATTGACTCTGATGCGAGCGCGGAAGACACCGCTTCAATTTCTGACAACGAAGATGTTGAACAAATTGAAGAAGAGAATGTCTCAACTGCTGATGTAGAAGATGTTTCAGCTGTAGAAGAAGAAACTGTAGCAGTGGTAAACGTTGAGAACCCTGCTGAACAAGTCACCCTTGTAGAGGCTAACCCTGCTGTGCAACTTTCTGCAGTAGAGGAAAAAGCTGAAGTCACAGCTAAAAATGATCCGTGGGCTGCTGCTGTTGATGAACAGGAAGCTGAACAACCTAAACCTTCCTTTATACCATTCAAGACGACAAATCTTAATGGTAAAATCATACTTAAACAACAAGATAATAATTGTTGGATAAATGCTTGCTGTTACCAACTGCAAGCCTTTGATTTCTTTAATCATGATTTGTGGGAGGCTTTTAAAAAGGATGATGTCATGCCTTTCGTGAACTTTTGTTATGCAGCTCTAACTTTGAAGCAGGGTGATTCAGGGGATGCAGAATACCTTTTAGAGACGATGCTCAATGATTATAGTACAGCTAGAGTTACACTTAGTGCTAAGTGTGGCTGTGGTATTAAGGAAATAGTTTTAGAACGAACTGTGTTTAAGCTTACACCTCTTAGGAATGAGTTTAAATATGGTGTCTGTGGTGACTGCAAACAAATTAACATGTGTAAGTTTGTTAGTGTGGAAGGTTCAGGTGTCTTTGTTCATGATAGAATTGAAAAACAAACACCTGTGTCACAATTCATTGTGCCACcTACTATGCATGCTGTTTACACAGGTACAACACAGAGTGGCCATTACATGATTGAGGATTGCATTCACGACTACTGTGTGGATGGCATGGGACTTAAACCTCGTAAACATAAGTTTTACACATCCACTTTGTTTCTCAATGCAAATGTAATGACAGCTGAAGCTAAAACTAAGATCGAACCACCTGTGCCCGCTAGTGATAAGTGCGTGGAAGAAAGATGTCAAAGTCCTAAAGATCTTATGACACCCTTTTATAAAGCGGGAAAAGTTTCATTTTACCAAGGAGATTTGGATGTTTTGATTAACTTTCTTGAACCTGACGTCATTGTTAACGCTGCTAATGGTGATCTTAAGCACATGGGTGGCGTTGCCCATGCTATTGATGTGTTCACAGGTGGCAAGTTGACAAAACGCTCTAAAGAGTATCTTAAATCCAACAAGGCAATAGCTCCTGGTAATGCTGTGCTTTTTGAAAATGTACTTGAGCATCTTAGTGTTTTAAATGCTGTAGGACCACGTAATGGTGACAGTAGAGTTGAAGGTAAACTCTGTAATGTTTACAAAGCAATTGCCAAGTGCGACGGTAGGATACTAACACCACTTATTAGTGTTGGTATCTTCAAAGTTAAACTGGAGATCTCATTGCAGTGTCTACTTAAAACTGTCACAGATAGAGAGTTAAATGTCTTTGTTTACACTGACCAAGAGAGAATTACCATAGAAAATTTTTTCAATGGTACTATCCCTGTCAAGGTAACGGAAGATACTGTGAATCAAAAGCGTGTGTCCGTAGCACTTGATAAAACTTATGGTGAGCAACTTAAAGGAACTGTTGTTATTAAAGATAAAGATGTTACTAACCAGTTGCCTAGTGTATCAGATGCAGGTGAGAAGGTTGTTAAAGCGCTTGATGTTGATTGGAGTGCACATTATGGCTTTCCAAATGCAGCGGCTTTTAGTGCCAGCTCCCACGATGCTTATAAATTTGAAGTTGTAATACATAACAATTTCATTGTTCATAAGCAGACTGATAACAATTGTTGGGTTAATGCAATTTGTTTAGCATTACAGAGACTTAAACCTACATGGAAGTTTCCTGGTGTTAAAAGTTTGTGGGATGATTTTATCACGCGTAAAACTGCTGGATTTGTCCATATGCTATATCACATTTCGGGCTTAAATAAAGGGCAACCTGGTGATGCCGAGTTGACTTTACATAGGCTCGGTGAGTTGATGTCAAGTGACAGCGCAGTCACCGTCACACATACAACTGCATGTGATAAGTGTGCAAAAGTTGAAACTTTCACTGGACCCGTGGTAGCAGCACCTCTTATGATTTGCGGCACTGATGAAACTTGTGTTCATGGTGTGAGTGTCAATGTTAAAGTGACAAGTGTCAGAGGTACGGTGGCTATCACTTCTTTAATAGGTCCTGTAGTGGGAGATGTTATTGATGCAACTGGTTATATCTGTTATACTGGTTTGAATTCACGCGGCCATTACACATACTATGATAATCGTAACTGTTTAATGGTCGATGCTGAGAAGGCCTACCATTTTGAGAGAAACCTCTTACAGGTTACGACAGCCATCGCTAGTAATTTCGTTGTTAAAGCATCTAAGATGGAGACTTTGCCTAAGAGCCAAGTTAAAGAACCAAGCAACACACGTGTTTTTAGTGAGGTTGAAGAAACTCCTAAAAATATAGTGCGTGAGTCAAAATTGTTGGCTATTGAGAGTGGTGCAGATTATACAATCTCAACACTCGGTAAATATGCTGACGTGTTCTTTATGACTGGTGACAAAATTCTTAGACTTCTCCTTGAAATCTTTAAATATTTATTGGTTGTTTTTATGTGTCTTAGAAAATCTAAGATGCCTAAAGTTAAAGTAAAACCACCGCATGTTTTTAAGGACATAGGTGCTAAAGTTAGAACGTTGAATTACGTGAGACAACTGAATAAACCAGCCTTGTGGCGTTACGCAAAACTAGTGCTACTTTTAATAGCATTGTACCACTTTTTCTACTTGTTTGTCAGCATACCATTAGTGCATAAAATGGCATGTAGTAGTAGTGTTCAAGCATATAGTAATTCTAGTTTTGTAAAATCAGAAGTCTGTGGTAATTCTATTCTGTGTAAGGCATGTCTGGCATCTTATGATGAATTGGCAGATTTTGATCATCTCCAAGTATCATGGGATTACAAGTCAGACCCGCTGTGGAATAGAGTTTTGCAATTGTCTTACTTTGCATTCTTGGCTGTTTTTGGTAACAATTATGTTAGATGTGTTCTTATGTATTTTGTTTCTCAGTACCTCAATCTGTGGTTGTCATATTTTGGTTATGTTAAGTATAGCTGGTTTCTGCATGTCATCAACTTTGAATCAATTTCTGTTGAATTTGTGATCATAGTTGTAGTTTTTAAGGCAGTTCTCGCACTTAAACATATTTTCTTTCCATGCAGCAACCCTTCATGTAAAACATGTTCTAAAATTGCTAGGCAGACGCGCATTCCTATACAGGTTGTGGTTAATGGTTCAATGAAGACTGTGTATGTCCATGCTAATGGTACTGGTAAACTCTGCAAAAAGCATAATTTTTATTGTAAGAATTGTGACTCTTATGGGTTTGATCACACATTTATTTGTGACGAGATTGTGCGTGACTTGAGTAATAGCGTTAAGCAAACAGTCTACGCTACTGACAGATCATACCAGGAAGTCACGAAAGTTGAATGTTCAGATGGTTTTTACAGATTCTATGTTGGTGAAGAATTCACTGCATATGACTATGATGTAAAACATAAGAAGTATAGTAGTCAGGAGGTGCTTAAGACTATGTTCTTGCTTGATGACTTCATTGTGTACAGCCCATCAGGTTCTTCTCTTGCTAGTGTTAGAAATGTCTGTGTTTACTTTTCACAACTAATTGGTAGGCCTATTAAGATTGTTAATAGTGATTTGCTTGAAGATTTGTCTGTAGACTTTAAGGGAGCACTTTTTAATGCCAAAAAGAATGTTATTAAGAATTCTTTTAACATTGATGTTTCAGAATGTAAAAACCTGGAAGAGTGTTATAAGGCGTGTAACCTTGATGTGACATTCTCCACTTTTGAAATGGCAGTTAATAATGCTCATAGGTTTGGTATATTAATTACAGACCGCTCTTTTAACAACTTCTGGCCATCCAAGATCAAGCCTGGTTCTTCTGGTGTCTCTGCTATGGACATTGGTAAGTGCATGACTTTTGATGCTAAGATTGTTAATGCAAAGGTTTTAACACAACGTGGTAAAAGTGTCGTATGGCTAAGTCAAGACTTTTCTGCACTTAGTTCTACGGCACAAAAGGTTTTAGTTAAAACATTTGTAGAGGAGGGTGTAAACTTTTCACTTACGTTTAATGCTGTGGGTTCAGATGAGGATTTGCCGTATGAGCGTTTCACTGAATCAGTTTCGGCAAAGAGCGGTTCAGGTTTCTTTGACGTACTCAAACAGCTTAAACAAATCTTTTGGTGTTTAGTTTTGTTCATCATTGTCTATGGTTTATGCTCAGTCTACAGTGTTGCAACGCAGTCTTATATTGATTCTGCTGAAGGTTATGAGTACATGGTTATTAAGAATGGTATTGTTCAACCATTTGATGATTCTATTAACTGTGTGCATAATACGTACAAAGGTTTTGCAGTGTGGTTTAAAGCTAAATATGGCTTTGTACCCACATTTGACAAGTCCTGCCCTATTGTTTTAGGAACTGTTTTTGATTTAGGTAACATGAGACCAATCCCGGATGTGCCAGCATATGTAGCACTTGTTGGTAGATCCTTAGTGTTTGCAATTAATGCTGCATTTGGTGTTACTAATGTGTGTTATGATCATATAGGTGCTGCTGTGAGTAAGAATTCTTACTTTGACACTTGTGTTTTCAATGCAGCATGCACTACCCTAGCTGGTCTTGGTGGTACAGTTGTCTATTGCGCTAAGCAAGGCCTTGTTGAGGGTGCTAAGCTTTACAGTGATTTGATGCCTGACTATTATTACGAGCACGCTAGTGGTAACATGGTAAAAATACCAGCTATTATTAGGGGTTTTGGTCTACGGTTTGTAAAAACACAGGCTACAACTTATTGCAGAGTGGGAGAGTGTACTGAAAGTCAAGCTGGTTTTTGCTTTGGTGGTGATAACTGGTTTGTCTATGATAAAGAGTTTGGAGACGGTTACATTTGTGGAAGTTCCATACTGGGATTCTTCAAAAATGTGTTCGCACTCTTTAATTCTAATATTTCAGTAGTGGCTACATCAGGTGTAATGTTGGTTAACATTGTCATTGCATGCTTGGCTATTGCAGCCTGTTATGGTGTTCTTAAGTTTAAAAAGATCTTTGGTGATTGCACTCTCCTAGTAGTTATGATTATTGTTACATTGATTGTTAATAATGTTTCTTACTTTGTAACCCAAAACACATTCTTTATGATTGTTTATGCTATTATCTATTATTTCACAACAAGAAAATTAGCATACCCAGGTATTTTAGATGCAGGGTTTATTATTGCATACTTGAATATGGCTCCGTGGTATGTTCTTGTTTTGTACATAATGGTATTCCTATATGATTCCCTACCATCACTATTCAAACTTAAGGTAACAACAAACCTTTTTGAAGGTGATAAATTTGTGGGTAGTTTTGAATCAGCAGCTATGGGTACTTTTGTTATAGACATGCGTTCATATGAAACACTTGTTAATTCTACATCTTTAGATAGAATTAAGTCATATGCTAACAGCTTTAACAAGTACAAGTACTATACTGGTTCTATGGGAGAGGCTGATTACCGCATGGCTTGTTATGCTCATCTGGGTAAGGCATTAATGGATTATTCTGTTTCAAGAAATGACATGCTTTACACGCCACCAACCGTCAGTGTTAATTCAACACTGCAGTCTGGATTGCGAAAAATGGCACAGCCTAGCGGTATCGTGGAACCCTGTGTTGTGAGGGTAGCCTATGGTAATAACGTCCTAAATGGTCTGTGGCTTGGGGATGAAGTTATCTGTCCTAGACATGTCATTGCTAGTGACACATCGCGTGTGATCAACTATGAGAATGAGTTGTCTAGTGTGCGTTTACATAACTTTTCTATAGCCAAAAATAATGTGTTTTTGGGTGTTGTGTCTGCAAAATATAAGGGTGTAAACCTTGTGCTTAAAGTGAACCAGGTAAACCCTAACACACCAGAACACAAATTTAAGTCTGTGAAATCAGGTGAGAGTTTTAACATTCTTGCTTGTTATGAGGGCTGTCCTGGTAGTGTCTATGGTGTGAACATGAGGAGTCAGGGTACTATCAAGGGTTCATTCATTGCTGGTACTTGCGGGTCAGTAGGTTATGTGTTAGAAAATGGAACTCTCTATTTTGTATACATGCACCACTTAGAGTTAGGTAATGGCTCTCATGTTGGTTCCAACCTTGAAGGTGAAATGTATGGCGGTTATGAAGATCAACCTAGCATGCAATTGGAAGGTACTAACGTCATGTCATCAGATAATGTGGTTGCATTTTTGTATGCTGCACTTATCAATGGTGAGAGATGGTTTGTCACAAATGCATCAATGTCGTTAGAATCCTACAATGCATGGGCCAAAACCAACAGTTTTACGGAAATTGTGTCAACTGATGCTTTTAATATGTTGGCCGCAAAAACTGGTTACAGTGTTGAAAAGTTGCTTGAGTGTATTGTTAGACTCAACAAAGGTTTTGGAGGACGCACTATATTGTCTTATGGCTCACTATGTGATGAATTCACACCCACAGAAGTTATAAGGCAAATGTATGGTGTTAATCTTCAGAGTGGTAAAGTTAAATCTTTGTTTTACCCTGTGATGACTGCTATGGCCATCCTTTTTGCATTTTGGTTGGAGTTCTTCATGTACACACCCTTTACCTGGATTAATCCAACATTTGTTAGTGTTATTCTAGCTATTACAACACTCGTATCTGTGATACTAGTTGCTGGAATCAAGCACAAAATGTTGTTCTTTATGTCGTTTGTAATGCCTAGTGTTATTTTAGCTACTGCATATAATGTGGTCTGGGATTTGACCTATTATGAAAGTTTACAGGTTCTTGTAGAAAATGTTAATACCACCTTTTTACCAGTTGACATGCAGGGTGTTATGCTTGCACTGTTTTGTGTTGTTGTGTTTGTTACATATACTATAAGATTTTTCACATGTAAGCAATCATGGTTTTCATTGTTTGTAACAACTGTTTTTGTGGTGTTTAACATTGTTAAATTGCTAGGTATGGTAGGTGAACCATGGACTGAAGACCACATTTTGCTGTGTCTTGTAAACATGCTTACCATGTTGATAAGTCTTACTACAAAGGACTGGTTTGTTGTTTTTGCATCATACAAAGTTTCTTATTATATAGTTGTATATGTAATGCAGCCAGCTTTTGTTCAAGATTTTGGTTTCGTCAAATGTATAAGCATAATTTACATGGCCTGTGGTTACCTTTTCTGTTGTTATTATGGTATCCTTTATTGGGTTAACAGATTTACATGCATGACATGTGGCGTTTACCAGTTCACAGTTTCATCAGCAGAGCTTAAATACATGACTGCCAATAACTTATCTGCACCTAAGACAGCGTACGATGCTATGATTCTTAGTGTTAAATTGATGGGCATTGGTGGAGAGCGAAACATAAAAATCTCCACTGTGCAGTCTAAACTCATAGAGATGAAGTGTACGAATGTAGTTTTGCTCGGCCTGTTGTCTAAGATGCATGTTGAGTCTAATTCAAAGGAGTGGAATTATTGTGTTAGCTTGCATAATGAGATCAATTTAAGTGATGACCCCGAAGCCGTCCTTGAAAAATTATTAGCACTTATTGCATTCTTTTTGTCTAAGCACAACACATGTGATCTTAGTGATCTTATTGAGTCTTATTTTGAGAATACCACTATCTTACAGAGTGTGGCATCTGCTTATGCTGCTTTGCCTAGCTGGATTGCCTATGAAAAAGCTCGTGCCGACTTAGAAGAGGCTAAGAAGAATGACGTTAGCCCTCAACTTCTGAAACAGCTTACAAAGGCTTGTAATATCGCTAAGAGCGAATTTGAGCGAGAAGCGTCTGTGCAAAAGAAGCTTGATAAAATGGCAGAGCAGGCTGCAGCTAGCATGTACAAAGAGGCACGTGCCGTGGACAGGAAGTCAAAAATAGTTTCAGCTATGCATAGTTTACTTTTTGGTATGCTTAAGAAACTTGACATGTCCAGTGTTAATACTATCATTGAACAGGCGCGTAATGGCGTGTTACCATTGAGTATTATACCAGCTGCCTCAGCTACAAGACTTATTGTTGTAACGCCTAACCTTGAAGTGCTTTCCAAGGTTAGACAAGAAAACAATGTACACTATGCAGGTGCTATCTGGTCAATTGTTGAAGTCAAGGATGCTAATGGCGCTCAAGTCCACTTGAAGGAAGTTACAGCCGCCAACGAGTTGAATTTAGCCTGGCCTTTGAGTATTACATGTGAGAGAACCACAAAGTTGCAGAACAATGAAATTTTGCCAGGTAGACTCAAGGAGAGAGCTGTTAAAGCATCTGCTACAATTGATGGTGATGCTTATGGTAGTGGTAAAGCGCTTATGGCTTCTGAAAATGGTAAAAGTTTCATCTATGCATTTATAGCATCAGACAGCAACCTTAAGTATGTTAAATGGGAGAGCAACAATGATGTGATACCTATTGAACTTGAGGCTCCATTGCGCTTTTATGTTGATGGTGTTAATGGTCCTGAGGTTAAATACTTGTATTTTGTGAAAAACTTAAACACTCTTAGACGTGGTGCTGTTCTTGGTTACATCGGTGCAACAGTTCGTTTGCAAGCCGGTAAACCCACTGAACACCCATCTAATAGTGGTTTGTTGACGTTATGTGCTTTTGCACCTGACCCTGCTAAAGCATATGTTGATGCTGTTAAAAGAGGTATGCAGCCAGTTACTAACTGTGTAAAGATGCTCTCAAATGGTGCCGGTAATGGTATGGCTATTACGAATGGTGTCGAATCTAACACACAACAGGATTCTTATGGTGGTGCATCTGTGTGTATTTATTGTAGATGCCATGTAGAACATCCCGCAATTGATGGGTTGTGCCGCTTTAAAGGTAAATTCGTCCAAGTACCAACCGGCACTCAGGATCCTATTAGATTCTGTATTGAAAATGAAGTCTGTGTCGTTTGCGGTTGCTGGCTTAATAATGGTTGTATGTGTGATCGCACTTCTATGCAAGGCACCACTATCGATCAGAGTTATTTAAACGAGTGCGGGGTTCTAGTGCAGCTCGACTAGAACCCTGTAATGGTACTGATCCAGACCATGTTAGTAGAGCTTTTGACATCTACAATAAAGATGTTGCGTGTATTGGTAAATTCCTTAAGACTAATTGTTCCCGTTTTAGGAATTTAGACAAACATGATGCCTACTACGTAGTCAAGCGGTGTACTAAGAGCGTTATGGACCATGAGCAAGTCTGTTATAACGATCTTAAAGATTCAGGCGCAGTTGCTGAACATGACTTCTTTTTGTACAAGGAAGGTCGGTGTGAATTCGGTAACGTTGCACGTAAGGATCTTACAAAGTACACAATGATGGATCTGTGTTATGCTATTAGAAACTTTGATGAAAAGAACTGTGAAGTCCTTAAAGAGATACTTGTGACATTAGGTGCCTGCAATGAATCTTTCTTTGAAAACAAAGATTGGTTTGACCCAGTAGAAAATGAGGCTATACATGAGGTATACGCAAGACTTGGACCTATAGTAGCCAACGCTATGCTTAAATGTGTAGCGTTTTGTGATGCTATTGTGGAAAAAGGTTACATAGGTATTATAACTCTTGATAACCAAGATCTTAATGGCAATTTTTACGATTTCGGTGATTTTGTAAAAACAGCGCCAGGTTTTGGATGTGCTTGCGTTACATCATATTATTCTTATATGATGCCCTTAATGGGAATGACTTCATGTTTAGAGTCTGAAAATTTTGTGAAAAGTGACATCTATGGTTCAGATTATAAGCAGTATGACTTATTAGCTTATGATTTCACGGATCATAAGGAGAAACTTTTCCAAAAATACTTTAAGCACTGGGATCGAACATACCACCCTAATTGCTCTGATTGTACAAGTGATGATTGCATTATTCATTGTGCTAACTTTAACACGTTGTTTTCAATGACCATACCTAATACTGCATTTGGACCTCTTGTACGTAAAGTTCATATAGATGGTGTACCAGTTGTTGTTACAGCTGGTTATCATTTTAAACAGTTAGGTATTGTGTGGAATCTTGATGTTAAATTAGACACTATGAAGCTGACTATGACAGATCTTCTTAGGTTTGTCACTGACCCTACGTTACTTGTAGCCTCAAGCCCCGCATTGTTAGATCAGCGCACTGTCTGTTTCTCCATTGCAGCTTTGAGTACTGGTGTTACATATCAGACAGTGAAACCAGGTCACTTTAACAAGGATTTCTACGATTTTATAACAGAGCGTGGATTCTTTGAGGAGGGATCTGAGTTGACATTAAAACACTTTTTCTTTGCACAGGGTGGTGAAGCTGCTATGACAGACTTTAATTATTACCGTTACAACAGAGTTACTGTTTTAGACATCTGTCAAGCGCAATTTGTTTACAAAATTGTGTGTAAGTATTTTGAGTGTTATGATGGAGGTTGCATCAATGCTCGAGAGGTTGTTGTTACCAACTATGATAAGAGTGCTGGTTACCCACTTAATAAATTTGGTAAGGCAAGACTTTACTATGAGACTCTATCATATGAGGAGCAGGATGCGCTTTTTGCTTTAACAAAGAGAAATGTGCTACCCACAATGACTCAAATGAATTTGAAATATGCTATTTCTGGAAAGGCTAGAGCTCGTACTGTGGGAGGAGTGTCGCTTCTTTCGACAATGACAACGAGACAGTACCACCAGAAACATTTGAAATCTATTGCCGCCACACGTAATGCTACCGTAGTTATTGGGACAACTAAATTCTATGGTGGCTGGGACAACATGCTAAAGAATTTGATGCGTGACGTAGATAATGGCTGTTTGATGGGATGGGACTATCCAAAGTGCGACCGTGCTTTGCCTAATATGATACGAATGGCTTCTGCTATGGTATTGGGTTCTAAGCACGTTGGGTGTTGTACACATAGTGATAGATTCTATCGACTCTCTAATGAGTTAGCTCAAGTACTTACCGAGGTTGTACATTGTACAGGTGGTTTTTATATAAAACCAGGTGGTACAACTAGTGGTGATGGTACTACAGCATATGCCAACTCAGCTTTTAACATCTTTCAAGCTGTTTCTGCTAATGTTAATAAGCTCTTAGGAGTGGATTCAAACACCTGTAACAACGTTACAGTTAAGTCTATACAACGCAAGATATATGACAATTGTTATCGTAGTAGCAGTGTTGATGACGACTTTGTTGTCGAATACTTTAGTTACTTAAGAAAGCACTTTTCTATGATGATTTTGTCTGATGATGGTGTTGTTTGTTACAACAAAGATTATGCTGATCTTGGTTATGTAGCAGACATTGGTGCATTTAAGGCTACCTTATATTATCAGAACAATGTATTTATGTCTACAGCTAAGTGTTGGGTAGAACCAGATCTTAATGTTGGACCACATGAATTTTGTTCGCAACACACTCTACAGATTGTAGGACCTGATGGTGATTACTACCTACCTTATCCAGACCCTTCTAGAATTTTGTCAGCAGGTGTTTTTGTTGATGATATTGTTAAGACAGACAACGTCATTATGCTTGAACGTTATGTGTCTTTAGCAATTGATGCATATCCACTCACAAAACACCCTAAACCTGCATATCAAAGAGTATTCTATGCTCTTCTTGATTGGGTTAAGCACTTACAGAAGACTCTAAATGCTGGTATACTTGACTCATTCTCTGTCACTATGTTAGGGGATGGTCAAGATAAATTCTGGAGTGAAGAATTTTATGCCAGTCTTTATGAAAAGTCTACTGTTTTGCAAGCTGCTGGTATGTGTGTTGTTTGTGGTTCACAAACTGTGTTACGTTGCGGAGACTGTTTAAGGAGACCTCTCTTGTGTACCAAGTGTGCCTACGACCATGTCATGGGTACAAaGCATAAaTTCATTATGTCTATCACACCATATGTGTGTAGTTATAATGGTTGCACTGTCAATGATGTTACAAAATTGTTTTTGGGAGGTCTTAATTATTACTGTACTGAACACAAACCACAATTATCATTCCCGCTCTGTGCTAATGGTAATGTGTTTGGATTGTACAAGAGTAGTGCAATTGGTTCTGAAGATGTTGATGATTTCAACAAACTTGCTGTTTCAGACTGGACCAATGTAGAGGATTATAAACTCGCTAACAATGTTAAAGAAACTTTGAAAATCTTCGCTGCTGAAACTGTGAAAGCAAAGGAGGAGTCTGTTAAAGCTGAATATGCTTATGCCATATTAAAGGAGGTAGTCGGCCCTAAGGAAGTTGTACTCCAATGGGAATCCTCTAAGATTAAACCTCCACTTAACAGAAATTCTGTTTTCACATGTTTTCAGATAAACAAGGATACTAAAATTCAGTTAGGTGAATTTGTGTTTGAGCAGTCAGAATATGGTAGTGACTCTGTTTACTATAAAAGTACAAGCACTACTAAGCTGGCACCGGGTATGGTTTTTGTGTTGACGTCTCATAATGTGAGTCCACTTAAAGCTAGCATTTTAGTCAACCAAGAGAAGTACAATACCATATCCAAGCTCTATCCTACGTTCAACATAGCGGAGGCCTATACCACATTGGTGCCTTACTATCAAATGATTGGTAAGCAAAAATTTACGACTATCCAAGGTCCTCCTGGTAGTGGTAAATCACATTGTGTTATAGGTTTGGGTCTGTATTATCCTCAAGCTAGAATTGTCTACACAGCATGTTCACATGCAGCAGTTGATGCTTTATGTGAAAAAGCTTCCAAGAACTTTAATGTTGATAAATGTTCAAGGATAATACCTCAAAGAATCAGAGTTGATTGCTATATGGGTTTTAAACCTAATAATACCAATGCACAATATTTGTTTTGCACAGTTAATGCTTTGCCTGAATCTAACTGTGATATTGTGGTTGTGGATGAAGTGTCAATGTGTACAAACTATGATCTCAGTGTTATAAATAGTAGACTGAGCTATAAACATATCGTTTACGTAGGTGATCCACAACAACTTCCAGCACCTAGAACCCTGATTAACAAAGGTACACTCCAGCCTGAGGATTACAACGTTGTGACTCAGAGGATGTGTAAACTAGGACCTGATGTATTCTTGCACAAATGTTATAGATGCCCAGCTGAAATTGTTAAAACAGTCTCTGCGCTCGTTTATGAGAATAAGTTCTTACCTGTCAACCCTGAGTCAAAGCAGTGCTTTAAGATGTTTGTAAAAGGTCAAGTTCAGATTGAATCTAACTCTTCTATAAACAACAAGCAACTAGAGGTTGTCAAGGCATTTTTAGTACATAACCCAAAATGGCGTAAAGCTGTTTTTATCTCACCCTATAACAGTCAAAATTATGTGGCACGACGTCTACTAGGTCTGCAAACTCAAACCGTAGACTCTGCGCAAGGTAGTGAGTATGATTATGTCATCTACACACAGACATCCGACACACAACATGCTATTAATGTCAACAGATTTAATGTTGCCATTACTAGAGCAAAAATTGGCATTCTCTGTGTCATGTGTGATAGAAGGATGTATGATAATCTTGATTTCTATGAACTCAAAGATTCAAAGATTGGCTTGCAGGCAAAAccTGAAACTTGTGGTTTGTTCAAAGATTGCTCAAAGAATGACCAGTATATACCACCAGCATATGCTACGACATATATGAGTTTGTCTGATAATTTTAAGACAAGTGACGGCTTAGCTGTTAACATCGGCACAAAGGATGTTAAATATGCTAACGTTATCTCATATATGGGGTTCAGGTTCGAGGCCAATGTACCAGGTTATCACACATTGTTTTGCACAAGAGACTTTGCTATGCGTAATGTGAGAGCATGGCTTGGTTTTGATGTCGAAGGTGCACATGTCTGTGGTGACAATATTGGAACTAATGTACCACTACAGCTGGGCTTTTCAAATGGTGTTGATTTCGTAGTACAAACTGAAGGATGTGTTGTTACTGAAAAAGGTAATAGCATTGAAGTTGTAAAAGCAAGAGCGCCACCGGGTGAGCAATTTGCACATTTGATACCACTCATGAGGAGAGGTCAATCCTGGCACATTGTTAGACGTCGTATAGTGCAGATGGTTTGTGACTATTTCGATGGCTTGTCAGACATCTTAATTTTTGTGCTATGGGCTGGTGGTCTTGAGCTTACAACTATGCGATACTTTGTTAAGATTGGAAAACCACAAAGATGTGAGTGCGGCAAAATGGCAACTTGCTATAGTAGCTCCCAATGTGTCTATGCTTGTTTTAAACATGCATTAGGATGTGACTATTTGTATAATCCTTATTGCATTGACATTCAACAATGGGGCTACACAGGTTCTCTGAGCATGAACCATCATGAAGTTTGTAACATTCATAGAAATGAGCATGTCGCTAGTGGTGATGCTATCATGACTAGATGCCTTGCTATATATGATTGTTTTGTTAAACGTGTAGATTGGTCCATTGTGTACCCTTTTATTGAAAACGAAGAGAAGATCAATAAAGCTGGTCGCATTGTACAATCACATGTCATGAGAGCTGCTCTTAAAGTTTTCAACCCTGCTGCAATTCACGATGTTGGTAATCCAAAAGGTATTCGTTGTGCTACGACACCCATACCATGGTTTTGTTATGATCGTGACCCTATTAACAATAATGTTAGATGTCTGGAGTATGATTACATGGTACATGGACAAATGAATGGTTTAATGTTGTTTTGGAATTGTAACGTGGACATGTACCCAGAGTTCTCAATTGTTTGTAGATTTGACACTCGAACGCGCTCAAAGCTGTCATTAGAAGGTTGTAATGGTGGTGCATTGTATGTTAACAATCATGCCTTTCACACACCAGCTTATGATAGAAGAGCATTTGCCAAGCTTAAACCTATGCCATTCTTTTATTACGATGAAAGTGACTGTGAGTTTATTGATGGACAACCTAATTACGTACCACTTAAGTCCAATGTTTGCATAACTAAATGTAACATTGGTGGTGCAGTCTGCAAGAAACATGCCGCACTCTATAGAGCATATGTTGAGGACTACAATGTGTTTATACAAGCAGGCTTTACAATTTGGTGTCCTCAAAATTTTGACACTTATATGTTGTGGCAAGGTTTTGTTAATGGCAAAGCACTCCAGAGTTTAGAAAATGTAGCTTTTAATGTCGTCAAGAAAGGTGCCTTCGCTGATTTAAAAGGCGACTTACCAACAGCTGTTGTAGCTGATAAGATCATGGTGAGGGATGGACCTACTGACAAGTGTATTTTCACAAATAAAACTAGTTTGCCTACAAATGTGGCTTTTGAGCTCTATGCAAAGCGCAAACTTGGACTCACACCTCCATTAACAATACTTAGGAATCTAGGTGTTGTCGCAACACATAAATTTGTGTTGTGGGATTACGAAGCTGAATGTCCTTTCTCAAACTTCACTAAGCAAGTGTGTGCTTACACTGATCTTGACGGTGAAGTTGTAACATGTTTTGATAATAGTATTAGTGGTTCTTTCGAACGCTTTACTACTACGAAAGATGCAGTGCTTATTTCTAATAACGCTGTGAAAGGACTTAGTGCCATTAAATTACAATATGGCTTTTTGAATGATTTACCTGTAAGTACTGTGGGAAACAAACCTGTCACATGGTATATCTATGTGCGCAAGAATGGCGAGTACGTCGAACAGATTGACAGTTATTACACACAAGGACGTACTTTTGAAACCTTCAAACCTCGTAGTACAATGGAAGAAGACTTTCTTAGTATGGATACTACACTCTTCATCCAAAAGTATGGTCTTGAGGATTATGGTTTCGAACACGTTGTATTTGGAGATGTTTCTAAAACTACCATCGGTGGTATGCATCTTCTTATATCACAAGTGCGCCTTGCAAAAATGGGTTTGTTTTCTGTCCAAGAATTTATGAATAATTCTGACAGTACACTGAAAAGTTGTTGTATAACATATGCTGATGATCCAGCTTCTAAGAATGTGTGCACTTATATGGACATACTCTTGGACGACTTTGTGACCATTGTTAAGAGCTTAGATCTTAACGTTGTGTCGAAAGTTGTGGATGTTATTGTAGATTGTAAGGCATGGAGATGGATGTTGTGGTGTGAGAATTCACAAATTAAAACCTTCTATCCACAACTCCAATCTTCCGAGTGGAATCCGGGTTATAGCATGCCTACACTTTACAAGATACAGCGTATGTGTCTCGAACGGTGTAATCTCTACAATTATGGTGCACAAGTGAAGTTACCTGACGGCATTACTACTAATGTCGTTAAGTATACCCAGTTGTGTCAATACCTCAATACCACTACACTGTGTGTCCCACATAAAATGCGCGTTCTGCACTTAGGGGCAGCAGGTGCTAATGGTGTTGCTCCTGGTACCACAGTATTAAAAAGATGGTTGCCAGATGATGCCATATTGGTTGATAACGATATGAGAGATTACGTTTCCGACGCAGACTTCAGTGTTACAGGTGATTGTACTAACCTCTATATTGAGGATAAATTTGATTTACTTATATCTGATTTATATGATGGCTCAACCAAGTCTATAGACGGTGAAAATACGTCAAAAGATGGCTTCTTCACATACATTAATGGTTTTATTAATGAGAAGCTAGCACTTGGAGGTTCTGTTGCCATCAAAATCACTGAATTTAGTTGGAATAAAAGTTTATATGAATTAATTCAAAGATTTGAGTATTGGACTGTGTTTTGTACAAGTGTTAACACCTCGTCATCAGAAGGTTTTCTGGTAGGTATTAATTACTTAGGACCATACAGTGACAAGGCCATAGTGGATGGGAATATAATGCATGCCAATTATATATTTTGGAGAAACTCTACAATTATGGCATTGTCACATAACTCAGTTCTAGATACTCCTAAATTTAAGTGTCGCTGTAATAACGCACTTGTTGTTAATTTAAAAGAAAGAGAATTAAATGATATGGTTGTTGGATTGCTAAGGAAGGGTAAGTTACTCATTAGAAATAATGGCAAGCTACTAAACTTTGGTAATCATTTAGTTAATGTGCCATGATTGTGCTCGTAACTTGCCTCTTGTTGTTATGTTCATACCACACAGTTTTGAGTACAACAAATAATGAATGCATACAAGTTAACGTAACACAATTGGCTGGCAATGAAAACCTTATCAGAGATTTTCTGTTTAGTAACTTTAAAGAAGAAGGAAGTGTAGTTGTTGGTGGTTATTACCCTACAGAGGTGTGGTACAACTGCTCTAGAACAGCTCGAACTACTGCCTTTCAGTATTTTAATAATATACATGCCTTTTATTTTGTTATGGAAGCCATGGAAAATAGCACTGGTAATGCACGTGGTAAACCATTATTATTTCATGTGCATGGTGAGCCTGTTAGTGTTATTATATCGGCTTATAGGGATGATGTGCAACAAAGGCCCCTTTTAAAACATGGGTTAGTGTGCATAACTAAAAATCGCCATATTAACTATGAACAATTCACCTCCAACCAGTGGAATTCCACATGTACGGGTGCTGACAGAAAAATTCCTTTCTCTGTCATACCCACGGACAATGGAACAAAAATCTATGGTCTTGAGTGGAATGATGACTTTGTTACAGCTTATaTTAGTGGTCGTTCTTATCACTTGAACATCAATACTAATTGGTTTAACAATGTCACACTTTTGTATTCACGCTCAAGCACTGCTACCTGGGAATACAGTGCTGCATATGCTTACCAAGGTGTTTCTAACTTCACTTATTACAAGTTAAATAACACCAATGGTCTAAAAACCTATGAATTATGTGAAGATTATGAACATTGCACTGGCTATGCTACCAATGTATTTGCTCCGACATCAGGTGGTTACATACCTGATGGATTTAGTTTTAACAATTGGTTCTTGCTTACAAATAGTTCCACTTTTGTTAGTGGCAGGTTTGTAACAAATCAACCATTATTGATTAATTGCTTGTGGCCAGTGCCCAGTTTTGGTGTAGCAGCACAAGAATTTTGTTTTGAAGGTGCACAGTTTAGCCAATGTAATGGTGTGTCTTTAAATAACACAGTGGATGTTATTAGATTCAACCTTAATTTCACTGCAGATGTACAATCTGGTATGGGTGCTACAGTATTTTCACTGAATACAACAGGTGGTGTCATTCTTGAAATTTCATGTTATAGTGACACAGTGAGTGAGTCTAGTTCTTACAGTTATGGTGAAATCCCGTTCGGCATAACTGACGGACCACGATACTGTTATGTACTTTACAATGGCACAGCTCTTAAATATTTAGGAACATTACCACCCAGTGTAAAGGAAATTGCTATTAGTAAGTGGGGCCATTTTTATATTAATGGTTACAATTTCTTTAGCACATTTCCTATTGGTTGTATATCTTTTAATTTAACCACTGGTGTTAGTGGAGCTTTTTGGACAATTGCTTACACATCGTATACTGAAGCATTAGTACAAGTTGAAAACACAGCTATTAAAAATGTGACGTATTGTAACAGTCACATTAATAACATTAAATGTTCTCAACTTACTGCTAATTTGAATAATGGATTTTATCCTGTTGCTTCAAGTGAAGTAGGTTTCGTTAATAAGAGTGTTGTGTTATTACCTAGCTTTTTCACATACACCGCTGTCAATATAACCATTGATCTTGGTATGAAGCTTAGTGGTTATGGTCAACCCATAGCCTCGACACTAAGTAACATCACACTACCAATGCAGGATAACAATACTGATGTGTACTGTATTCGTTCTAACCAATTCTCAGTTTATGTTCATTCCACTTGCAAAAGTTCTTTATGGGACAATATTTTTAATCAAGACTGCACGGATGTTTTAGAGGCTACAGCTGTTATAAAAACTGGTACTTGTCCTTTCTCATTTGATAAATTGAACAATTACTTGACTTTTAACAAGTTCTGTTTGTCGTTGAGTCCTGTTGGTGCTAATTGCAAGTTTGATGTTGCTGCACGTACAAGAACCAATGAGCAGGTTGTTAGAAGTCTATATGTAATATATGAAGAAGGAGACAACATAGTGGGTGTACCGTCTGATAATAGCGGTCTGCACGATTTGTCTGTGCTACACCTAGACTCCTGTACAGATTACAATATATATGGTAGAACTGGTGTTGGTATTATTAGACGAACTAACAGTACGCTACTTAGTGGCTTATATTACACATCACTATCAGGTGATTTGTTAGGCTTTaAAAATGTTAGTGATGGTGTCATTTATTCTGTGACGCCATGTGATGTAAGCGCACAAGcGGCTGTTATTGATGGTGCCATAGTTGGAGCTATGACTTCCATTAACAGTGAACTGTTAGGTCTAACACATTGGACAACGACACCTAATTTTTATTACTACTCTATATATAATTACACAAGTGAGAGGACTCGTGACACTGCAATTGACAGTAACGATGTTGATTGTGAACCTGTCATAACCTATTCTAATATAGGTGTTTGTAAAAATGGTGCTTTGGTTTTTATTAACGTCACACATTCTGACGGAGACGTGCAACCAATTAGCACTGGTAATGTCACGATACCTACAAATTTTACCATATCTGTGCAAGTTGAATACATGCAGGTTTACACTACACCAGTATCAATAGATTGTGCAAGATACGTTTGTAATGGTAACCCTAGATGTAACAAATTGTTAACACAATATGTGTCTGCATGTCAAACTATTGAACAAGCACTTGCAATGGGTGcCAGACTTGAAAACATGGAGGTTGATTCCATGTTGTTTGTCTCGGAAAATGCCCTTAAATTGGCATCTGTTGAGGCGTTCAATAGTACAGAAAATTTAGATTCTATTTACAAAGAATGGCCTAGCATAGGTGGTTCTTGgCTAGGAGGTCTAAAAGATATACTACCGTCCCATAATAGCAAACGtAAGTATGGTTCTGCTATAGAAGATTTGCTTTTTGATAAAGTTGTAACATCTGGTTTAGGTACAGTTGATGAAGATTATAAACGTTGTACTGGTGGTTACGACATAGCAGACTTGGTGTGTGCTCAATATTACAATGGCATCATGGTTCTACCAGGTGTAGCTAATGCTGACAAGATGACTATGTACACAGCATCACTTGCAGGTGGTATAACATTAGGTGCACTTGGTGGTGGCGCCGTGGCTATACCTTTTGCAGTAGCAGTACAGGCTAGACTTAATTATGTTGCTCTACAAACTGATGTATTGAATAAAAACCAACAGATCCTGGCTAATGCCTTCAATCAAGCTATTGGTAACATTACACAGGCTTTTGGTAAGGTTAATGATGCTATACATCAAACATCACAAGGTCTTGCCACTGTTGCTAAAGCGTTGGCAAAAGTGCAAGATGTTGTCAACACACAAGGGCAAGCTTTAAGTCACCTTACAGTACAATTGCAAAATAATTTTCAAGCCATTAGTAGTTCTATTAGTGATATTTATAACAGGCTTGACGAACTGAGTGCTGATGCACAAGTTGATAGGCTGATTACAGGTAGACTTACAGCACTTAATGCATTTGTGTCTCAGACTCTAACCAGACAAGCAGAGGTTAGGGCTAGTAGACAACTTGCCAAAGACAAGGTTAATGAATGTGTTAGGTCTCAGTCTCAGAGATTCGGATTCTGTGGTAATGGTACACATTTGTTTTCACTAGCAAATGCAGCACCAAATGGCATGATTTTCTTTCATACAGTACTATTACCAACAGCTTATGAAACTGTAACAGCTTGGTCAGGTATTTGTGCTTCAGATGGCGATCGCACTTTCGGACTTGTCGTTAAAGATGTGCAGTTGACGTTGTTTCGTAATCTAGATGACAAGTTCTATTTGACCCCCAGAACTATGTATCAGCCTAGAGTTGCAACTAGTTCTGATTTTGTTCAAATTGAAGGGTGTGATGTGTTGTTTGTCAACGCGACTGTAATTGATTTGCCTAGTATTATACCTGACTATATTGACATTAATCAAACTGTTCAAGACATATTAGAAAATTACAGACCAAACTGGACTGTACCTGAATTTACACTTGATATTTTCAACGCAACCTATTTaAATCTGACTGGTGAAATTGATGACTTAGAGTTTAGGTCAGAAAAGCTACATAACACTACAGTAGAACTTGCCATTCTCATTGATAACATTAATAATACATTAGTCAATCTTGAATGGCTCAATAGAATTGAAACTTATGTAAAATGGCCGTGGTATGTGTGGCTACTAATCGGTTTAGTAGTGGTCTTCTGCATACCATTGTTACTATTTTGCTGTCTGAGTACTGGATGTTGTGGGTGCTTTGGTTGCCTTGTAAGTTGTTGCAATTCTCTTTGTAGTAGAAGACAATTTGAAAGCTACGAACCTATCGAAAAGGTTCACATCCATTAACTAAACGATTTATGGACGCCGTCAAGTCTATTGGCATCTCTGTTGACGCTGTACTTGACGAATTAGATTCCATTGCTTTTGCTGTAACACTTAAAGTTTTATTTAATTCTGGTAAATTACTTGTGTGTATAGGTTTTGGTGATACTTTTGAAGAGGCTGAACAAAAAGCTTATGCCAAATCAAAACTGGTATAAGTTACCTTTTGTAGTCAGATTACGTATCATAAATAATACAAAACCTAGAACAGCAAGCACTATAAAACGCAGACGAAGGGCTGTTATTGACTACATAAAAATCGCTATTCTCAACGCGACGCGAAAATGATTGGCGGACTGTTTCTTAACGCTCTAAGTTTTATAGTTACTAATCAACATGTGATTGCTAATAATACATCACATGTTAATACTATAGTACAACAACACCATGTTGTTAGTGCTAATATACCTATTAAAAGTTTTCATTTAGAGTTCAGCATTGCTGTGCTCTTCGTTTTATTTTTAGCTTTGTACCGTAGTACAAACTTTAAAGTGTGTGTCGGTGTCTTAATGTTTAAGATAGTATCAATGACACTTATAGGACCTATGCTCATAGCATTTGGTTACTACATAGATGGCATTGTGACAATAACTGTCTTAGCTTTAAGATTTATTTACTTAGCATACTTTTGGTATGTTAATAGTAGATTTGAATTCGTTTTATACAATACGACGACACTAATGTTTGTACACGACAGAGCTGCACCGTTTATGAGAAGTTCTCACGGCTCTATTTGTGTCACACTATACGGAGGCATAAATTACATGTTCGTGAATGATCTTACGTTGCATTTTGTAGATCCTATGCTTGGCATATCTATACGTGGCCTAGTTCATGCTGACCTAACAGTTGTTAGAGCAGTTGAACTTCTCAATGGTGATTTTATCTATATATTTTCACAGGAGGCCGTCGTAGGAGTTTACAATGCAGCTTTTTCTCAGGCGGTTATAAACGAAATTGATTTGAAAGACGAAGAAGAGCGTGTCTATGATGTTTCCTAGGGCATTTACTATCATAGACGACCATGGTATGGTTGTTAGCGTTTTCTTCTGGCTCCTGTTGATAATTATATTGATATTGTTTTCAATAGCATTGCTAAATGTTATTAAATTGTGTATGGTGTGCTGCAATTTGGGTAAGACTATTGTAGTACTACCTGCTCGCCATGCATATGATGCCTATAAGACCTTTATGCAGATTAAGGCATACAATCCCGACGAAGCATTTTTGGTTTGAACTAAACAAAATGAAGTACATTTTGTTAATACTCGCGTGCATTATTGCATGCGTTTATGGAGAACGCTACTGTGCCATGCAAAATACAGGCTCgCAGTGCATTAATGGCACAGATTCATCATGTAGCACCTGTTTTGAACGTGGTGgTCTTATTTGGCATCTGGCTAACTGGAACTTCAGCTGGTCTGTAATATTGATTGTTTTTATAACAGTGTTAaAATATGGAAGACCGCAATTCAGCTGGCTCGTTTATGGCATTAAAATGCTGATCATGTGGCTATTATGGCCTATTGTTCTAGCGCTTACGATTTTTAATGCATACTCTGAGTACCAAGTTTCCAGATATGTAATGTTCGGCTTTAGTGTTGCAGGTGCAGTTGTAACGTTTGCACTATGGATGATGTATTTTGTGAGATCTATTCAGCTGTATAGACGGACCAAATCATGGTGGTCTTTTAATCCTGAAACCAATGCGATTCTTTGTGTCAATGCATTGGGTAGAAGCTATGTACTCCCTCTTGATGGCACTCCTACAGGTGTTACTCTTACCCTACTTTCAGGAAATCTATACGCTGAAGGTTTTAAAATGGCTGGTGGTCTTACCATCGAGCATTTGCCTAAATATGTCATGATTGCTACGCCTAGTAGAACCATCGTTTACACATTAGTTGGAAAACAACTAAAGGCAACTACTGCCACTGGATGGGCTTACTATGTAAAATCTAAAGCTGGTGATTACTCAACAGAAGCACGTACTGATAATTTGAGTGAACATGAAAAATTATTACATATGGTGTAACTAAACTTCTAAATGGCCACACAGGGACAACGCGTCAACTGGGGAGATGAACCTTCCAAAAGACGTGGTCGTTCTAACTCTCGTGGTCGGAAGAATAACACTATACCTCTTTCATTCTTCAATCCCATCCAACTCGAACCAGGATCAAAATTTTGGAGCGTATGTCCGAGAGATTTTGTTCCCAAGGGAATAGGTAACAAGGATCAACAAATTGGTTATTGGAATAGACAAGAGCGTTACCGCATTGTCAAAGGTCAGCGTAAGGAACTTCCTGAGAGGTGGTTTTTCTACTTCTTAGGCACAGGACCTCAAGCTGATGCTAAATTTAAAGACAAGATTGATGGAGTCTTCTGGGTTGCAAAGGATGGTGCCATGAATAAACCAACAACACTTGGCACTCGTGGTACCAACAATGAATCCAAACCACTGAAATTTGATGGTAAGATACCACCGCAATTTCAGCTTGAAGTGAACCGATCTAGGAACAACTCAAGAAGTGGTTCTCAGTCTAGATCTGTCTCTAGAAACAGGTCTCAATCCAGAGGAAGACAACAATCCAATAATCAGAATAATGTTGAGGATACAATTGTAGCTGTGCTTCAGAAATTAGGTGTTACTGAAAAGCAAAGGTCACGTTCTAAATCTAGAGATCGTGGTGACTCTAAACCTAGAGACACAACACCTAATAATGCCAACAAACACACCTGGAAGAAGACTGCAGGTAAAGGTGATGTGACAAATTTCTATGGTGCTAGAAGTGCTTCAGCTAACTTTGGTGATAGTGATCTCGTTGCCAACGGTAACGCTGCCAAATCCTACCCTCAGATAGCTGAATGCGTTCCATCAGTGTCTAGCATGCTCTTCGGTAGTCAATGGTCTGCTGAAGATGATGGTGATCAAGTGAAAGTCACGCTCACTCATACCTATTACCTGCCAAAAGATGATGCCAAAACCAGCCAATTCCTAGAACAGATTGACGCTTACAAGCGGCCATCTCAAGTGGCTAAAGATCAGAGGCAAAGAAAATCTCGTTCTAAGTCTGCTGAGAAGAAGCCTGAGGAATTGTCTGTAACTCTTGTAGAGGCATATACGGATGTGTTTGATGACACACAGGTTGAGATGATTGATGAGGTTACGAACTAAACGCATGCTCGTTTTCCTCCATGCTGTACTTGTTACAGTTTTAATCTTACCACTAATTGGTAGAATCCAATTACTGGAAAGGTTATTACTCAGTCATCTGCTTAATCTTACAACAGTCAGTAATGTTTTAGGTGTGCCTGACAGTAGTCTGCGTGTAAATTGCTTACAGCTTTTGAAACCAGACTGTCTTGATTTTAACATCTTACACAAAGTTTTAGCAGAAACCAGATTACTAGTAGTAGTACTGCGAGTGATCTTTCTGGTTCTTCTAGGGTTTTCCTGCTATACATTGCTAGGTGCATTATTTTAACATCATGATTGTTGTACTCCTTGTGTGTGTTTTCTTGGCTAATGGACTTAAAGCTACTATTGTGCAATCTGACCCTCATGAACATCCAGTTCTTACATGGGAACTTTTGCAACATTTCATAGGAAGCACTCTCTACATTACAACACATCAGATTTTAGCATTACCGCTTGGATCGCGTGTTGAGTGTGAGAGTGTTGAAGGTTTCAATTGCACATGGCCTGGTTTTCAAAAGCCTGCACATGATCACATTGATTTCTATTTTGATCTTTCTAATCCTTTCTATTCCTTTGTAGATAATTTTTATATTGTAGGTGATGGAAATCAAAGAATTAATCTTAGATTAGTTGGTGCTGTGCCAAAACAAAAGAGATTAAATGTTGGTTGTTATATATCATTTGCTGTTGACCTTCCATTTGGAACTCAGATATACCATGACAGAGATTTTCAACACCCTGTTAGTGGTAGACATCTAGAGTGTACTCACAGAGTCTACTTTGTGAAGTATTGTCCACACAACCTGTATGGTTATTGCTTTAATGAGAAGCTGAAAGTTTATAACTTGACGCAACTCAGAAGCAAGAAGGTTTTCGACAAAATCAACCAACATCATAAAACTGAGTTATAAGGCAACCCGATGTCTAAAACTGGTCTTTCCGAGGAATTG**EnUmt-FIPV**

AGCGTTGATTATTTCACTCAGTTTGGCAATCACTCCTAGGAACGGGGTTGAGAGAACGGCGCACCAGGGTTCCGTCCCTGTTTGGTAAGTCGTCTAGTATTAGCTGCGGCGGTTCCGCCCGTCGTAGTTGGGTAGACCGGGTTCCGTCCTGTGATCTCCCTCGCCGGCCGCCAGGAGAATGAGTTCCAAACAATTTAAGATCCTCGTTAATGAGGACTACCAAGTCAACGTGCCTAGCCTTCCTTTCCGTGACGTGCTGCAGGACATTAAGTACTGCTACCGGAACGGTTTTGATGGCTATGTCTTCGTGCCTGAATACCGTCGTGACCTAGTTGATTGCAATCGTAAGGATCACTACGTCATTGGTGTTTTGGGTAACGGAATAAGTGATCTTAAACCTGTCCTCCTTACCGAACCTTCCGTCATGTTGCAGGGTTTTATTGTTAGAGCTGACTGCAATGGCGTTCTTGAGGACTTTGACCTTAAAATTGCCCGTACTGGAAATGGTGCCATATATGTAGACCAATACATGTGTGGTGCCGATGGAAAGCCAGTCATTGAAGGTGAGTTTAAGGACTATTTCGGTGATGAGGACGTTATTATCTATGAAGGAGAGGAGTATCATTGTGCCTGGTTAACAGTGCGCGATGAAAAACCTTTGTGTCAACAAACTCTCCTTACAATTAGGGAAATCCAATACAATCTGGACGTCCCACATAAGTTGCCAAATTGTGCTATCAGAGAGGTAGCACCACCAGTCAAGAAGAACTCCAAGGTTGTTCTTTCTGAAGAGTATAAAAAGCTCTATGACATCTTCGGTTCACCATTTATGGGCAATGGGGACAGTCTTAACAAGTGTTTCGACAGCCTCCACTTCATCGCTGCTACTCTTAAGTGTCCTTGTGGCTCTGAAAGTAGTGGTGTTGGTGACTGGACTGGTTTTAAGACTGCTTGTTGTGGTCTTCATGGCAAAGTCAGAGGTGTCACACTAGGTGCCGTTAAACCTGGTGATGCTGTTATCACTAGCATGAGCGCTGGCAAGGGTGTAAAATTCTTTGCTAATAGTGTGCTCCAATATGCTGGTGATGTTGAAAATGTCTCTGTTTGGAAAGTGATTAAAACTTTCACTGTTAACGAGACTGTTTGCACCGCCGATTTTGAAGGCGAATTGAACGACTTCATCAAACCAGAGAGTAAGTCACTGGTTTCGTGCTCTATTAAGAGGGCTTTCATCACTGGTGAAGTTGATGATGCAGTCCATGATTGTATTATCACTGGAAAGTTGGACCTTAGTACTAATCTTTTTGGTAGTGCTAGCCTACTTTTTGAGAAAACACCCTGGTTTGTGCAAAAGTGTGGTGCACTTTTTGCTGATGCTTGGAAAGTTATTGAAGAACTGCTTTGTTCTCTTAAACTTACGTACAAGCAAATCTATGATGTAGTGGCATCACTTTGCACTTCTGCCTTCACTATTATGGACTACAAACCTGTGTTTGTAGTATCGGCTAATAGTGTTAAGGACCTCGTTGACAAGTGTGTCAAAATTCTTGTGAAAGCCTTTGATGTTTTCACACAGACAATTACAATTGCGGGTGTAGAGGCTAAGTGCTTTGTGCTTGGTTCTAAATACTTGCTTTTTAACAACGCACTTGTCAAGCTTGTCAGTGTTAAAATACTTGGCAAGAGACAGAAAGGTCTTGATAGTGCATTCTTTGCTACTAACTTAATTGGTGCGACTGTTAATGTGACACCGCAGAGGACTGAGGCTGCTAACATTAGCCTGAATAAGGTTGATGATGTTGCTACACCAGGTGAAGGTCACATTGTCATCATTGGCGACATGGCTTTTTACAAGAGTGAAGAATACTATTTTATGATGGCAAGTCCAGATTCAGTCCTTGTTAATAATGTTTTTAAAGCAGCTCGAGTGCCGTCTTATAACATCACTTATGATGTGGATGATGATACCAAGAGTAAGATGTTAGTTAAAATTGGAACATCATTTGATTTTGATGGTGATCTTGACGCTGCTATTGCTAAAGTTAATGACTTACTTATAGAGTTTAGACAAGACAAGTTGTGTTTCAGAGCACTTAAGGATTGTGAAAACATCTTAGTTGAGGCATACCTTAAGAAATACAAGATGCCAGTTTGTCTCAAAAACCATGTTGGTTTGTGGGACATCATAAGGCGAGATTCTGACAAGAAGGGTTTTCTTGACACTTTCAATCATCTTAATGAATTGGAGGATGTCAAGGACACAAAGATTCAGTCTATCAAAGATATTCTTTGTCCTGACCTTCTGCTTGAATTGGACTTTGGCGCAATCTGGTATAGATGTATGCCTGTCTGCTCTGATGTGTCTATCCTAGGTAATGTTAAAATTATGCTAGGCAATGGTGTTAAAGTGCTTTGCGATGGCTGTAACAGTTTTGCCAAACGTTTAACAGTTAGTTACAGCAAACTGTGTGATACAGCTCGTAAAGATGTTGAAATTGGTGGCTTACCGTTTTCAACTTTTAAAACACCAGCTAGCAGTTTTATCGACATGAAAGATGCCATCTATTCTGTAGTTGAATATGGTGAAGCATTTTCTTTCAAGACTGCAATTGTACCTGTAATTAATAGCGGCACCGTCACAACCGACGATTGGTCAGACCCCATATTGTTAGAACCTGCAGACTATGTTGAACCTAAAGATAACGGCGATATAATTGTCATTGCTGGATACACCTTCTACAAGGATGAAGATGATCATTTTTATCCATATGGTTCTGGCATGGTTGTGCAGAAGATGTACAACAAGATGGGTGGTGGTGACAAATCAGTTTCATTCTCAGACGATGTTAATGTTAAAGAGATCGAACCTATTACACGTGTCAGGCTAGAATTTGAGTTTGACAATGAGGTTGTAACACAGGTTCTTGAAAAAGTCATTGGTACCAAATATAAGTTTATAGGTACCACCTGGGAAGAGTTTGAAGATTCTATCTCTGAGAAACTTGACAATATCTTTGACAAGCTTGCTGAACAGGGTGTAGAACTTGAAGGTTACTTCATTTATGACACATGTGGTGGATTTGACATTAACAATCCAGATGGTGTTATGATCTCACAATATGATCTTAACACAGATGTTGAAGACAAAATTGACTCTGATGCGAGCGCGGAAGACACCGCTTCAATTTCTGACAACGAAGATGTTGAACAAATTGAAGAAGAGAATGTCTCAACTGCTGATGTAGAAGATGTTTCAGCTGTAGAAGAAGAAACTGTAGCAGTGGTAAACGTTGAGAACCCTGCTGAACAAGTCACCCTTGTAGAGGCTAACCCTGCTGTGCAACTTTCTGCAGTAGAGGAAAAAGCTGAAGTCACAGCTAAAAATGATCCGTGGGCTGCTGCTGTTGATGAACAGGAAGCTGAACAACCTAAACCTTCCTTTATACCATTCAAGACGACAAATCTTAATGGTAAAATCATACTTAAACAACAAGATAATAATTGTTGGATAAATGCTTGCTGTTACCAACTGCAAGCCTTTGATTTCTTTAATCATGATTTGTGGGAGGCTTTTAAAAAGGATGATGTCATGCCTTTCGTGAACTTTTGTTATGCAGCTCTAACTTTGAAGCAGGGTGATTCAGGGGATGCAGAATACCTTTTAGAGACGATGCTCAATGATTATAGTACAGCTAGAGTTACACTTAGTGCTAAGTGTGGCTGTGGTATTAAGGAAATAGTTTTAGAACGAACTGTGTTTAAGCTTACACCTCTTAGGAATGAGTTTAAATATGGTGTCTGTGGTGACTGCAAACAAATTAACATGTGTAAGTTTGTTAGTGTGGAAGGTTCAGGTGTCTTTGTTCATGATAGAATTGAAAAACAAACACCTGTGTCACAATTCATTGTGCCACcTACTATGCATGCTGTTTACACAGGTACAACACAGAGTGGCCATTACATGATTGAGGATTGCATTCACGACTACTGTGTGGATGGCATGGGACTTAAACCTCGTAAACATAAGTTTTACACATCCACTTTGTTTCTCAATGCAAATGTAATGACAGCTGAAGCTAAAACTAAGATCGAACCACCTGTGCCCGCTAGTGATAAGTGCGTGGAAGAAAGATGTCAAAGTCCTAAAGATCTTATGACACCCTTTTATAAAGCGGGAAAAGTTTCATTTTACCAAGGAGATTTGGATGTTTTGATTAACTTTCTTGAACCTGACGTCATTGTTAACGCTGCTAATGGTGATCTTAAGCACATGGGTGGCGTTGCCCATGCTATTGATGTGTTCACAGGTGGCAAGTTGACAAAACGCTCTAAAGAGTATCTTAAATCCAACAAGGCAATAGCTCCTGGTAATGCTGTGCTTTTTGAAAATGTACTTGAGCATCTTAGTGTTTTAAATGCTGTAGGACCACGTAATGGTGACAGTAGAGTTGAAGGTAAACTCTGTAATGTTTACAAAGCAATTGCCAAGTGCGACGGTAGGATACTAACACCACTTATTAGTGTTGGTATCTTCAAAGTTAAACTGGAGATCTCATTGCAGTGTCTACTTAAAACTGTCACAGATAGAGAGTTAAATGTCTTTGTTTACACTGACCAAGAGAGAATTACCATAGAAAATTTTTTCAATGGTACTATCCCTGTCAAGGTAACGGAAGATACTGTGAATCAAAAGCGTGTGTCCGTAGCACTTGATAAAACTTATGGTGAGCAACTTAAAGGAACTGTTGTTATTAAAGATAAAGATGTTACTAACCAGTTGCCTAGTGTATCAGATGCAGGTGAGAAGGTTGTTAAAGCGCTTGATGTTGATTGGAGTGCACATTATGGCTTTCCAAATGCAGCGGCTTTTAGTGCCAGCTCCCACGATGCTTATAAATTTGAAGTTGTAATACATAACAATTTCATTGTTCATAAGCAGACTGATAACAATTGTTGGGTTAATGCAATTTGTTTAGCATTACAGAGACTTAAACCTACATGGAAGTTTCCTGGTGTTAAAAGTTTGTGGGATGATTTTATCACGCGTAAAACTGCTGGATTTGTCCATATGCTATATCACATTTCGGGCTTAAATAAAGGGCAACCTGGTGATGCCGAGTTGACTTTACATAGGCTCGGTGAGTTGATGTCAAGTGACAGCGCAGTCACCGTCACACATACAACTGCATGTGATAAGTGTGCAAAAGTTGAAACTTTCACTGGACCCGTGGTAGCAGCACCTCTTATGATTTGCGGCACTGATGAAACTTGTGTTCATGGTGTGAGTGTCAATGTTAAAGTGACAAGTGTCAGAGGTACGGTGGCTATCACTTCTTTAATAGGTCCTGTAGTGGGAGATGTTATTGATGCAACTGGTTATATCTGTTATACTGGTTTGAATTCACGCGGCCATTACACATACTATGATAATCGTAACTGTTTAATGGTCGATGCTGAGAAGGCCTACCATTTTGAGAGAAACCTCTTACAGGTTACGACAGCCATCGCTAGTAATTTCGTTGTTAAAGCATCTAAGATGGAGACTTTGCCTAAGAGCCAAGTTAAAGAACCAAGCAACACACGTGTTTTTAGTGAGGTTGAAGAAACTCCTAAAAATATAGTGCGTGAGTCAAAATTGTTGGCTATTGAGAGTGGTGCAGATTATACAATCTCAACACTCGGTAAATATGCTGACGTGTTCTTTATGACTGGTGACAAAATTCTTAGACTTCTCCTTGAAATCTTTAAATATTTATTGGTTGTTTTTATGTGTCTTAGAAAATCTAAGATGCCTAAAGTTAAAGTAAAACCACCGCATGTTTTTAAGGACATAGGTGCTAAAGTTAGAACGTTGAATTACGTGAGACAACTGAATAAACCAGCCTTGTGGCGTTACGCAAAACTAGTGCTACTTTTAATAGCATTGTACCACTTTTTCTACTTGTTTGTCAGCATACCATTAGTGCATAAAATGGCATGTAGTAGTAGTGTTCAAGCATATAGTAATTCTAGTTTTGTAAAATCAGAAGTCTGTGGTAATTCTATTCTGTGTAAGGCATGTCTGGCATCTTATGATGAATTGGCAGATTTTGATCATCTCCAAGTATCATGGGATTACAAGTCAGACCCGCTGTGGAATAGAGTTTTGCAATTGTCTTACTTTGCATTCTTGGCTGTTTTTGGTAACAATTATGTTAGATGTGTTCTTATGTATTTTGTTTCTCAGTACCTCAATCTGTGGTTGTCATATTTTGGTTATGTTAAGTATAGCTGGTTTCTGCATGTCATCAACTTTGAATCAATTTCTGTTGAATTTGTGATCATAGTTGTAGTTTTTAAGGCAGTTCTCGCACTTAAACATATTTTCTTTCCATGCAGCAACCCTTCATGTAAAACATGTTCTAAAATTGCTAGGCAGACGCGCATTCCTATACAGGTTGTGGTTAATGGTTCAATGAAGACTGTGTATGTCCATGCTAATGGTACTGGTAAACTCTGCAAAAAGCATAATTTTTATTGTAAGAATTGTGACTCTTATGGGTTTGATCACACATTTATTTGTGACGAGATTGTGCGTGACTTGAGTAATAGCGTTAAGCAAACAGTCTACGCTACTGACAGATCATACCAGGAAGTCACGAAAGTTGAATGTTCAGATGGTTTTTACAGATTCTATGTTGGTGAAGAATTCACTGCATATGACTATGATGTAAAACATAAGAAGTATAGTAGTCAGGAGGTGCTTAAGACTATGTTCTTGCTTGATGACTTCATTGTGTACAGCCCATCAGGTTCTTCTCTTGCTAGTGTTAGAAATGTCTGTGTTTACTTTTCACAACTAATTGGTAGGCCTATTAAGATTGTTAATAGTGATTTGCTTGAAGATTTGTCTGTAGACTTTAAGGGAGCACTTTTTAATGCCAAAAAGAATGTTATTAAGAATTCTTTTAACATTGATGTTTCAGAATGTAAAAACCTGGAAGAGTGTTATAAGGCGTGTAACCTTGATGTGACATTCTCCACTTTTGAAATGGCAGTTAATAATGCTCATAGGTTTGGTATATTAATTACAGACCGCTCTTTTAACAACTTCTGGCCATCCAAGATCAAGCCTGGTTCTTCTGGTGTCTCTGCTATGGACATTGGTAAGTGCATGACTTTTGATGCTAAGATTGTTAATGCAAAGGTTTTAACACAACGTGGTAAAAGTGTCGTATGGCTAAGTCAAGACTTTTCTGCACTTAGTTCTACGGCACAAAAGGTTTTAGTTAAAACATTTGTAGAGGAGGGTGTAAACTTTTCACTTACGTTTAATGCTGTGGGTTCAGATGAGGATTTGCCGTATGAGCGTTTCACTGAATCAGTTTCGGCAAAGAGCGGTTCAGGTTTCTTTGACGTACTCAAACAGCTTAAACAAATCTTTTGGTGTTTAGTTTTGTTCATCATTGTCTATGGTTTATGCTCAGTCTACAGTGTTGCAACGCAGTCTTATATTGATTCTGCTGAAGGTTATGAGTACATGGTTATTAAGAATGGTATTGTTCAACCATTTGATGATTCTATTAACTGTGTGCATAATACGTACAAAGGTTTTGCAGTGTGGTTTAAAGCTAAATATGGCTTTGTACCCACATTTGACAAGTCCTGCCCTATTGTTTTAGGAACTGTTTTTGATTTAGGTAACATGAGACCAATCCCGGATGTGCCAGCATATGTAGCACTTGTTGGTAGATCCTTAGTGTTTGCAATTAATGCTGCATTTGGTGTTACTAATGTGTGTTATGATCATATAGGTGCTGCTGTGAGTAAGAATTCTTACTTTGACACTTGTGTTTTCAATGCAGCATGCACTACCCTAGCTGGTCTTGGTGGTACAGTTGTCTATTGCGCTAAGCAAGGCCTTGTTGAGGGTGCTAAGCTTTACAGTGATTTGATGCCTGACTATTATTACGAGCACGCTAGTGGTAACATGGTAAAAATACCAGCTATTATTAGGGGTTTTGGTCTACGGTTTGTAAAAACACAGGCTACAACTTATTGCAGAGTGGGAGAGTGTACTGAAAGTCAAGCTGGTTTTTGCTTTGGTGGTGATAACTGGTTTGTCTATGATAAAGAGTTTGGAGACGGTTACATTTGTGGAAGTTCCATACTGGGATTCTTCAAAAATGTGTTCGCACTCTTTAATTCTAATATTTCAGTAGTGGCTACATCAGGTGTAATGTTGGTTAACATTGTCATTGCATGCTTGGCTATTGCAGCCTGTTATGGTGTTCTTAAGTTTAAAAAGATCTTTGGTGATTGCACTCTCCTAGTAGTTATGATTATTGTTACATTGATTGTTAATAATGTTTCTTACTTTGTAACCCAAAACACATTCTTTATGATTGTTTATGCTATTATCTATTATTTCACAACAAGAAAATTAGCATACCCAGGTATTTTAGATGCAGGGTTTATTATTGCATACTTGAATATGGCTCCGTGGTATGTTCTTGTTTTGTACATAATGGTATTCCTATATGATTCCCTACCATCACTATTCAAACTTAAGGTAACAACAAACCTTTTTGAAGGTGATAAATTTGTGGGTAGTTTTGAATCAGCAGCTATGGGTACTTTTGTTATAGACATGCGTTCATATGAAACACTTGTTAATTCTACATCTTTAGATAGAATTAAGTCATATGCTAACAGCTTTAACAAGTACAAGTACTATACTGGTTCTATGGGAGAGGCTGATTACCGCATGGCTTGTTATGCTCATCTGGGTAAGGCATTAATGGATTATTCTGTTTCAAGAAATGACATGCTTTACACGCCACCAACCGTCAGTGTTAATTCAACACTGCAGTCTGGATTGCGAAAAATGGCACAGCCTAGCGGTATCGTGGAACCCTGTGTTGTGAGGGTAGCCTATGGTAATAACGTCCTAAATGGTCTGTGGCTTGGGGATGAAGTTATCTGTCCTAGACATGTCATTGCTAGTGACACATCGCGTGTGATCAACTATGAGAATGAGTTGTCTAGTGTGCGTTTACATAACTTTTCTATAGCCAAAAATAATGTGTTTTTGGGTGTTGTGTCTGCAAAATATAAGGGTGTAAACCTTGTGCTTAAAGTGAACCAGGTAAACCCTAACACACCAGAACACAAATTTAAGTCTGTGAAATCAGGTGAGAGTTTTAACATTCTTGCTTGTTATGAGGGCTGTCCTGGTAGTGTCTATGGTGTGAACATGAGGAGTCAGGGTACTATCAAGGGTTCATTCATTGCTGGTACTTGCGGGTCAGTAGGTTATGTGTTAGAAAATGGAACTCTCTATTTTGTATACATGCACCACTTAGAGTTAGGTAATGGCTCTCATGTTGGTTCCAACCTTGAAGGTGAAATGTATGGCGGTTATGAAGATCAACCTAGCATGCAATTGGAAGGTACTAACGTCATGTCATCAGATAATGTGGTTGCATTTTTGTATGCTGCACTTATCAATGGTGAGAGATGGTTTGTCACAAATGCATCAATGTCGTTAGAATCCTACAATGCATGGGCCAAAACCAACAGTTTTACGGAAATTGTGTCAACTGATGCTTTTAATATGTTGGCCGCAAAAACTGGTTACAGTGTTGAAAAGTTGCTTGAGTGTATTGTTAGACTCAACAAAGGTTTTGGAGGACGCACTATATTGTCTTATGGCTCACTATGTGATGAATTCACACCCACAGAAGTTATAAGGCAAATGTATGGTGTTAATCTTCAGAGTGGTAAAGTTAAATCTTTGTTTTACCCTGTGATGACTGCTATGGCCATCCTTTTTGCATTTTGGTTGGAGTTCTTCATGTACACACCCTTTACCTGGATTAATCCAACATTTGTTAGTGTTATTCTAGCTATTACAACACTCGTATCTGTGATACTAGTTGCTGGAATCAAGCACAAAATGTTGTTCTTTATGTCGTTTGTAATGCCTAGTGTTATTTTAGCTACTGCATATAATGTGGTCTGGGATTTGACCTATTATGAAAGTTTACAGGTTCTTGTAGAAAATGTTAATACCACCTTTTTACCAGTTGACATGCAGGGTGTTATGCTTGCACTGTTTTGTGTTGTTGTGTTTGTTACATATACTATAAGATTTTTCACATGTAAGCAATCATGGTTTTCATTGTTTGTAACAACTGTTTTTGTGGTGTTTAACATTGTTAAATTGCTAGGTATGGTAGGTGAACCATGGACTGAAGACCACATTTTGCTGTGTCTTGTAAACATGCTTACCATGTTGATAAGTCTTACTACAAAGGACTGGTTTGTTGTTTTTGCATCATACAAAGTTTCTTATTATATAGTTGTATATGTAATGCAGCCAGCTTTTGTTCAAGATTTTGGTTTCGTCAAATGTATAAGCATAATTTACATGGCCTGTGGTTACCTTTTCTGTTGTTATTATGGTATCCTTTATTGGGTTAACAGATTTACATGCATGACATGTGGCGTTTACCAGTTCACAGTTTCATCAGCAGAGCTTAAATACATGACTGCCAATAACTTATCTGCACCTAAGACAGCGTACGATGCTATGATTCTTAGTGTTAAATTGATGGGCATTGGTGGAGAGCGAAACATAAAAATCTCCACTGTGCAGTCTAAACTCATAGAGATGAAGTGTACGAATGTAGTTTTGCTCGGCCTGTTGTCTAAGATGCATGTTGAGTCTAATTCAAAGGAGTGGAATTATTGTGTTAGCTTGCATAATGAGATCAATTTAAGTGATGACCCCGAAGCCGTCCTTGAAAAATTATTAGCACTTATTGCATTCTTTTTGTCTAAGCACAACACATGTGATCTTAGTGATCTTATTGAGTCTTATTTTGAGAATACCACTATCTTACAGAGTGTGGCATCTGCTTATGCTGCTTTGCCTAGCTGGATTGCCTATGAAAAAGCTCGTGCCGACTTAGAAGAGGCTAAGAAGAATGACGTTAGCCCTCAACTTCTGAAACAGCTTACAAAGGCTTGTAATATCGCTAAGAGCGAATTTGAGCGAGAAGCGTCTGTGCAAAAGAAGCTTGATAAAATGGCAGAGCAGGCTGCAGCTAGCATGTACAAAGAGGCACGTGCCGTGGACAGGAAGTCAAAAATAGTTTCAGCTATGCATAGTTTACTTTTTGGTATGCTTAAGAAACTTGACATGTCCAGTGTTAATACTATCATTGAACAGGCGCGTAATGGCGTGTTACCATTGAGTATTATACCAGCTGCCTCAGCTACAAGACTTATTGTTGTAACGCCTAACCTTGAAGTGCTTTCCAAGGTTAGACAAGAAAACAATGTACACTATGCAGGTGCTATCTGGTCAATTGTTGAAGTCAAGGATGCTAATGGCGCTCAAGTCCACTTGAAGGAAGTTACAGCCGCCAACGAGTTGAATTTAGCCTGGCCTTTGAGTATTACATGTGAGAGAACCACAAAGTTGCAGAACAATGAAATTTTGCCAGGTAGACTCAAGGAGAGAGCTGTTAAAGCATCTGCTACAATTGATGGTGATGCTTATGGTAGTGGTAAAGCGCTTATGGCTTCTGAAAATGGTAAAAGTTTCATCTATGCATTTATAGCATCAGACAGCAACCTTAAGTATGTTAAATGGGAGAGCAACAATGATGTGATACCTATTGAACTTGAGGCTCCATTGCGCTTTTATGTTGATGGTGTTAATGGTCCTGAGGTTAAATACTTGTATTTTGTGAAAAACTTAAACACTCTTAGACGTGGTGCTGTTCTTGGTTACATCGGTGCAACAGTTCGTTTGCAAGCCGGTAAACCCACTGAACACCCATCTAATAGTGGTTTGTTGACGTTATGTGCTTTTGCACCTGACCCTGCTAAAGCATATGTTGATGCTGTTAAAAGAGGTATGCAGCCAGTTACTAACTGTGTAAAGATGCTCTCAAATGGTGCCGGTAATGGTATGGCTATTACGAATGGTGTCGAATCTAACACACAACAGGATTCTTATGGTGGTGCATCTGTGTGTATTTATTGTAGATGCCATGTAGAACATCCCGCAATTGATGGGTTGTGCCGCTTTAAAGGTAAATTCGTCCAAGTACCAACCGGCACTCAGGATCCTATTAGATTCTGTATTGAAAATGAAGTCTGTGTCGTTTGCGGTTGCTGGCTTAATAATGGTTGTATGTGTGATCGCACTTCTATGCAAGGCACCACTATCGATCAGAGTTATTTAAACGAGTGCGGGGTTCTAGTGCAGCTCGACTAGAACCCTGTAATGGTACTGATCCAGACCATGTTAGTAGAGCTTTTGACATCTACAATAAAGATGTTGCGTGTATTGGTAAATTCCTTAAGACTAATTGTTCCCGTTTTAGGAATTTAGACAAACATGATGCCTACTACGTAGTCAAGCGGTGTACTAAGAGCGTTATGGACCATGAGCAAGTCTGTTATAACGATCTTAAAGATTCAGGCGCAGTTGCTGAACATGACTTCTTTTTGTACAAGGAAGGTCGGTGTGAATTCGGTAACGTTGCACGTAAGGATCTTACAAAGTACACAATGATGGATCTGTGTTATGCTATTAGAAACTTTGATGAAAAGAACTGTGAAGTCCTTAAAGAGATACTTGTGACATTAGGTGCCTGCAATGAATCTTTCTTTGAAAACAAAGATTGGTTTGACCCAGTAGAAAATGAGGCTATACATGAGGTATACGCAAGACTTGGACCTATAGTAGCCAACGCTATGCTTAAATGTGTAGCGTTTTGTGATGCTATTGTGGAAAAAGGTTACATAGGTATTATAACTCTTGATAACCAAGATCTTAATGGCAATTTTTACGATTTCGGTGATTTTGTAAAAACAGCGCCAGGTTTTGGATGTGCTTGCGTTACATCATATTATTCTTATATGATGCCCTTAATGGGAATGACTTCATGTTTAGAGTCTGAAAATTTTGTGAAAAGTGACATCTATGGTTCAGATTATAAGCAGTATGACTTATTAGCTTATGATTTCACGGATCATAAGGAGAAACTTTTCCAAAAATACTTTAAGCACTGGGATCGAACATACCACCCTAATTGCTCTGATTGTACAAGTGATGATTGCATTATTCATTGTGCTAACTTTAACACGTTGTTTTCAATGACCATACCTAATACTGCATTTGGACCTCTTGTACGTAAAGTTCATATAGATGGTGTACCAGTTGTTGTTACAGCTGGTTATCATTTTAAACAGTTAGGTATTGTGTGGAATCTTGATGTTAAATTAGACACTATGAAGCTGACTATGACAGATCTTCTTAGGTTTGTCACTGACCCTACGTTACTTGTAGCCTCAAGCCCCGCATTGTTAGATCAGCGCACTGTCTGTTTCTCCATTGCAGCTTTGAGTACTGGTGTTACATATCAGACAGTGAAACCAGGTCACTTTAACAAGGATTTCTACGATTTTATAACAGAGCGTGGATTCTTTGAGGAGGGATCTGAGTTGACATTAAAACACTTTTTCTTTGCACAGGGTGGTGAAGCTGCTATGACAGACTTTAATTATTACCGTTACAACAGAGTTACTGTTTTAGACATCTGTCAAGCGCAATTTGTTTACAAAATTGTGTGTAAGTATTTTGAGTGTTATGATGGAGGTTGCATCAATGCTCGAGAGGTTGTTGTTACCAACTATGATAAGAGTGCTGGTTACCCACTTAATAAATTTGGTAAGGCAAGACTTTACTATGAGACTCTATCATATGAGGAGCAGGATGCGCTTTTTGCTTTAACAAAGAGAAATGTGCTACCCACAATGACTCAAATGAATTTGAAATATGCTATTTCTGGAAAGGCTAGAGCTCGTACTGTGGGAGGAGTGTCGCTTCTTTCGACAATGACAACGAGACAGTACCACCAGAAACATTTGAAATCTATTGCCGCCACACGTAATGCTACCGTAGTTATTGGGACAACTAAATTCTATGGTGGCTGGGACAACATGCTAAAGAATTTGATGCGTGACGTAGATAATGGCTGTTTGATGGGATGGGACTATCCAAAGTGCGACCGTGCTTTGCCTAATATGATACGAATGGCTTCTGCTATGGTATTGGGTTCTAAGCACGTTGGGTGTTGTACACATAGTGATAGATTCTATCGACTCTCTAATGAGTTAGCTCAAGTACTTACCGAGGTTGTACATTGTACAGGTGGTTTTTATATAAAACCAGGTGGTACAACTAGTGGTGATGGTACTACAGCATATGCCAACTCAGCTTTTAACATCTTTCAAGCTGTTTCTGCTAATGTTAATAAGCTCTTAGGAGTGGATTCAAACACCTGTAACAACGTTACAGTTAAGTCTATACAACGCAAGATATATGACAATTGTTATCGTAGTAGCAGTGTTGATGACGACTTTGTTGTCGAATACTTTAGTTACTTAAGAAAGCACTTTTCTATGATGATTTTGTCTGATGATGGTGTTGTTTGTTACAACAAAGATTATGCTGATCTTGGTTATGTAGCAGACATTGGTGCATTTAAGGCTACCTTATATTATCAGAACAATGTATTTATGTCTACAGCTAAGTGTTGGGTAGAACCAGATCTTAATGTTGGACCACATGAATTTTGTTCGCAACACACTCTACAGATTGTAGGACCTGATGGTGATTACTACCTACCTTATCCAGACCCTTCTAGAATTTTGTCAGCAGGTGTTTTTGTTGATGATATTGTTAAGACAGACAACGTCATTATGCTTGAACGTTATGTGTCTTTAGCAATTGATGCATATCCACTCACAAAACACCCTAAACCTGCATATCAAAGAGTATTCTATGCTCTTCTTGATTGGGTTAAGCACTTACAGAAGACTCTAAATGCTGGTATACTTGACTCATTCTCTGTCACTATGTTAGGGGATGGTCAAGATAAATTCTGGAGTGAAGAATTTTATGCCAGTCTTTATGAAAAGTCTACTGTTTTGCAAGCTGCTGGTATGTGTGTTGTTTGTGGTTCACAAACTGTGTTACGTTGCGGAGACTGTTTAAGGAGACCTCTCTTGTGTACCAAGTGTGCCTACGACCATGTCATGGGTACAAaGCATAAaTTCATTATGTCTATCACACCATATGTGTGTAGTTATAATGGTTGCACTGTCAATGATGTTACAAAATTGTTTTTGGGAGGTCTTAATTATTACTGTACTGAACACAAACCACAATTATCATTCCCGCTCTGTGCTAATGGTAATGTGTTTGGATTGTACAAGAGTAGTGCAATTGGTTCTGAAGATGTTGATGATTTCAACAAACTTGCTGTTTCAGACTGGACCAATGTAGAGGATTATAAACTCGCTAACAATGTTAAAGAAACTTTGAAAATCTTCGCTGCTGAAACTGTGAAAGCAAAGGAGGAGTCTGTTAAAGCTGAATATGCTTATGCCATATTAAAGGAGGTAGTCGGCCCTAAGGAAGTTGTACTCCAATGGGAATCCTCTAAGATTAAACCTCCACTTAACAGAAATTCTGTTTTCACATGTTTTCAGATAAACAAGGATACTAAAATTCAGTTAGGTGAATTTGTGTTTGAGCAGTCAGAATATGGTAGTGACTCTGTTTACTATAAAAGTACAAGCACTACTAAGCTGGCACCGGGTATGGTTTTTGTGTTGACGTCTCATAATGTGAGTCCACTTAAAGCTAGCATTTTAGTCAACCAAGAGAAGTACAATACCATATCCAAGCTCTATCCTACGTTCAACATAGCGGAGGCCTATACCACATTGGTGCCTTACTATCAAATGATTGGTAAGCAAAAATTTACGACTATCCAAGGTCCTCCTGGTAGTGGTAAATCACATTGTGTTATAGGTTTGGGTCTGTATTATCCTCAAGCTAGAATTGTCTACACAGCATGTTCACATGCAGCAGTTGATGCTTTATGTGAAAAAGCTTCCAAGAACTTTAATGTTGATAAATGTTCAAGGATAATACCTCAAAGAATCAGAGTTGATTGCTATATGGGTTTTAAACCTAATAATACCAATGCACAATATTTGTTTTGCACAGTTAATGCTTTGCCTGAATCTAACTGTGATATTGTGGTTGTGGATGAAGTGTCAATGTGTACAAACTATGATCTCAGTGTTATAAATAGTAGACTGAGCTATAAACATATCGTTTACGTAGGTGATCCACAACAACTTCCAGCACCTAGAACCCTGATTAACAAAGGTACACTCCAGCCTGAGGATTACAACGTTGTGACTCAGAGGATGTGTAAACTAGGACCTGATGTATTCTTGCACAAATGTTATAGATGCCCAGCTGAAATTGTTAAAACAGTCTCTGCGCTCGTTTATGAGAATAAGTTCTTACCTGTCAACCCTGAGTCAAAGCAGTGCTTTAAGATGTTTGTAAAAGGTCAAGTTCAGATTGAATCTAACTCTTCTATAAACAACAAGCAACTAGAGGTTGTCAAGGCATTTTTAGTACATAACCCAAAATGGCGTAAAGCTGTTTTTATCTCACCCTATAACAGTCAAAATTATGTGGCACGACGTCTACTAGGTCTGCAAACTCAAACCGTAGACTCTGCGCAAGGTAGTGAGTATGATTATGTCATCTACACACAGACATCCGACACACAACATGCTATTAATGTCAACAGATTTAATGTTGCCATTACTAGAGCAAAAATTGGCATTCTCTGTGTCATGTGTGATAGAAGGATGTATGATAATCTTGATTTCTATGAACTCAAAGATTCAAAGATTGGCTTGCAGGCAAAAccTGAAACTTGTGGTTTGTTCAAAGATTGCTCAAAGAATGACCAGTATATACCACCAGCATATGCTACGACATATATGAGTTTGTCTGATAATTTTAAGACAAGTGACGGCTTAGCTGTTAACATCGGCACAAAGGATGTTAAATATGCTAACGTTATCTCATATATGGGGTTCAGGTTCGAGGCCAATGTACCAGGTTATCACACATTGTTTTGCACAAGAGACTTTGCTATGCGTAATGTGAGAGCATGGCTTGGTTTTGATGTCGAAGGTGCACATGTCTGTGGTGACAATATTGGAACTAATGTACCACTACAGCTGGGCTTTTCAAATGGTGTTGATTTCGTAGTACAAACTGAAGGATGTGTTGTTACTGAAAAAGGTAATAGCATTGAAGTTGTAAAAGCAAGAGCGCCACCGGGTGAGCAATTTGCACATTTGATACCACTCATGAGGAGAGGTCAATCCTGGCACATTGTTAGACGTCGTATAGTGCAGATGGTTTGTGACTATTTCGATGGCTTGTCAGACATCTTAATTTTTGTGCTATGGGCTGGTGGTCTTGAGCTTACAACTATGCGATACTTTGTTAAGATTGGAAAACCACAAAGATGTGAGTGCGGCAAAATGGCAACTTGCTATAGTAGCTCCCAATGTGTCTATGCTTGTTTTAAACATGCATTAGGATGTGACTATTTGTATAATCCTTATTGCATTGACATTCAACAATGGGGCTACACAGGTTCTCTGAGCATGAACCATCATGAAGTTTGTAACATTCATAGAAATGAGCATGTCGCTAGTGGTGATGCTATCATGACTAGATGCCTTGCTATATATGATTGTTTTGTTAAACGTGTAGATTGGTCCATTGTGTACCCTTTTATTGAAAACGAAGAGAAGATCAATAAAGCTGGTCGCATTGTACAATCACATGTCATGAGAGCTGCTCTTAAAGTTTTCAACCCTGCTGCAATTCACGATGTTGGTAATCCAAAAGGTATTCGTTGTGCTACGACACCCATACCATGGTTTTGTTATGATCGTGACCCTATTAACAATAATGTTAGATGTCTGGAGTATGATTACATGGTACATGGACAAATGAATGGTTTAATGTTGTTTTGGAATTGTAACGTGGACATGTACCCAGAGTTCTCAATTGTTTGTAGATTTGACACTCGAACGCGCTCAAAGCTGTCATTAGAAGGTTGTAATGGTGGTGCATTGTATGTTAACAATCATGCCTTTCACACACCAGCTTATGATAGAAGAGCATTTGCCAAGCTTAAACCTATGCCATTCTTTTATTACGATGAAAGTGACTGTGAGTTTATTGATGGACAACCTAATTACGTACCACTTAAGTCCAATGTTTGCATAACTAAATGTAACATTGGTGGTGCAGTCTGCAAGAAACATGCCGCACTCTATAGAGCATATGTTGAGGACTACAATGTGTTTATACAAGCAGGCTTTACAATTTGGTGTCCTCAAAATTTTGACACTTATATGTTGTGGCAAGGTTTTGTTAATGGCAAAGCACTCCAGAGTTTAGAAAATGTAGCTTTTAATGTCGTCAAGAAAGGTGCCTTCGCTGATTTAAAAGGCGACTTACCAACAGCTGTTGTAGCTGATAAGATCATGGTGAGGGATGGACCTACTGACAAGTGTATTTTCACAAATAAAACTAGTTTGCCTACAAATGTGGCTTTTGAGCTCTATGCAAAGCGCAAACTTGGACTCACACCTCCATTAACAATACTTAGGAATCTAGGTGTTGTCGCAACACATAAATTTGTGTTGTGGGATTACGAAGCTGAATGTCCTTTCTCAAACTTCACTAAGCAAGTGTGTGCTTACACTGATCTTGACGGTGAAGTTGTAACATGTTTTGATAATAGTATTAGTGGTTCTTTCGAACGCTTTACTACTACGAAAGATGCAGTGCTTATTTCTAATAACGCTGTGAAAGGACTTAGTGCCATTAAATTACAATATGGCTTTTTGAATGATTTACCTGTAAGTACTGTGGGAAACAAACCTGTCACATGGTATATCTATGTGCGCAAGAATGGCGAGTACGTCGAACAGATTGACAGTTATTACACACAAGGACGTACTTTTGAAACCTTCAAACCTCGTAGTACAATGGAAGAAGACTTTCTTAGTATGGATACTACACTCTTCATCCAAAAGTATGGTCTTGAGGATTATGGTTTCGAAgcaGTTGTATTTGGAGATGTTTCTAAAACTACCATCGGTGGTATGgcaCTTCTTATATCACAAGTGCGCCTTGCAAAAATGGGTTTGTTTTCTGTCCAAGAATTTATGAATAATTCTGACAGTACACTGAAAAGTTGTTGTATAACATATGCTGATGATCCAGCTTCTAAGAATGTGTGCACTTATATGGACATACTCTTGGACGACTTTGTGACCATTGTTAAGAGCTTAGATCTTAACGTTGTGTCGAAAGTTGTGGATGTTATTGTAGATTGTAAGGCATGGAGATGGATGTTGTGGTGTGAGAATTCACAAATTAAAACCTTCTATCCACAACTCCAATCTTCCGAGTGGAATCCGGGTTATAGCATGCCTACACTTTACAAGATACAGCGTATGTGTCTCGAACGGTGTAATCTCTACAATTATGGTGCACAAGTGAAGTTACCTGACGGCATTACTACTAATGTCGTTAAGTATACCCAGTTGTGTCAATACCTCAATACCACTACACTGTGTGTCCCACATAAAATGCGCGTTCTGCACTTAGGGGCAGCAGGTGCTAATGGTGTTGCTCCTGGTACCACAGTATTAAAAAGATGGTTGCCAGATGATGCCATATTGGTTGATAACGATATGAGAGATTACGTTTCCGACGCAGACTTCAGTGTTACAGGTGATTGTACTAACCTCTATATTGAGGATAAATTTGATTTACTTATATCTGATTTATATGATGGCTCAACCAAGTCTATAGACGGTGAAAATACGTCAAAAGATGGCTTCTTCACATACATTAATGGTTTTATTAATGAGAAGCTAGCACTTGGAGGTTCTGTTGCCATCAAAATCACTGAATTTAGTTGGAATAAAAGTTTATATGAATTAATTCAAAGATTTGAGTATTGGACTGTGTTTTGTACAAGTGTTAACACCTCGTCATCAGAAGGTTTTCTGGTAGGTATTAATTACTTAGGACCATACAGTGACAAGGCCATAGTGGATGGGAATATAATGCATGCCAATTATATATTTTGGAGAAACTCTACAATTATGGCATTGTCACATAACTCAGTTCTAGATACTCCTAAATTTAAGTGTCGCTGTAATAACGCACTTGTTGTTAATTTAAAAGAAAGAGAATTAAATGATATGGTTGTTGGATTGCTAAGGAAGGGTAAGTTACTCATTAGAAATAATGGCAAGCTACTAAACTTTGGTAATCATTTAGTTAATGTGCCATGATTGTGCTCGTAACTTGCCTCTTGTTGTTATGTTCATACCACACAGTTTTGAGTACAACAAATAATGAATGCATACAAGTTAACGTAACACAATTGGCTGGCAATGAAAACCTTATCAGAGATTTTCTGTTTAGTAACTTTAAAGAAGAAGGAAGTGTAGTTGTTGGTGGTTATTACCCTACAGAGGTGTGGTACAACTGCTCTAGAACAGCTCGAACTACTGCCTTTCAGTATTTTAATAATATACATGCCTTTTATTTTGTTATGGAAGCCATGGAAAATAGCACTGGTAATGCACGTGGTAAACCATTATTATTTCATGTGCATGGTGAGCCTGTTAGTGTTATTATATCGGCTTATAGGGATGATGTGCAACAAAGGCCCCTTTTAAAACATGGGTTAGTGTGCATAACTAAAAATCGCCATATTAACTATGAACAATTCACCTCCAACCAGTGGAATTCCACATGTACGGGTGCTGACAGAAAAATTCCTTTCTCTGTCATACCCACGGACAATGGAACAAAAATCTATGGTCTTGAGTGGAATGATGACTTTGTTACAGCTTATaTTAGTGGTCGTTCTTATCACTTGAACATCAATACTAATTGGTTTAACAATGTCACACTTTTGTATTCACGCTCAAGCACTGCTACCTGGGAATACAGTGCTGCATATGCTTACCAAGGTGTTTCTAACTTCACTTATTACAAGTTAAATAACACCAATGGTCTAAAAACCTATGAATTATGTGAAGATTATGAACATTGCACTGGCTATGCTACCAATGTATTTGCTCCGACATCAGGTGGTTACATACCTGATGGATTTAGTTTTAACAATTGGTTCTTGCTTACAAATAGTTCCACTTTTGTTAGTGGCAGGTTTGTAACAAATCAACCATTATTGATTAATTGCTTGTGGCCAGTGCCCAGTTTTGGTGTAGCAGCACAAGAATTTTGTTTTGAAGGTGCACAGTTTAGCCAATGTAATGGTGTGTCTTTAAATAACACAGTGGATGTTATTAGATTCAACCTTAATTTCACTGCAGATGTACAATCTGGTATGGGTGCTACAGTATTTTCACTGAATACAACAGGTGGTGTCATTCTTGAAATTTCATGTTATAGTGACACAGTGAGTGAGTCTAGTTCTTACAGTTATGGTGAAATCCCGTTCGGCATAACTGACGGACCACGATACTGTTATGTACTTTACAATGGCACAGCTCTTAAATATTTAGGAACATTACCACCCAGTGTAAAGGAAATTGCTATTAGTAAGTGGGGCCATTTTTATATTAATGGTTACAATTTCTTTAGCACATTTCCTATTGGTTGTATATCTTTTAATTTAACCACTGGTGTTAGTGGAGCTTTTTGGACAATTGCTTACACATCGTATACTGAAGCATTAGTACAAGTTGAAAACACAGCTATTAAAAATGTGACGTATTGTAACAGTCACATTAATAACATTAAATGTTCTCAACTTACTGCTAATTTGAATAATGGATTTTATCCTGTTGCTTCAAGTGAAGTAGGTTTCGTTAATAAGAGTGTTGTGTTATTACCTAGCTTTTTCACATACACCGCTGTCAATATAACCATTGATCTTGGTATGAAGCTTAGTGGTTATGGTCAACCCATAGCCTCGACACTAAGTAACATCACACTACCAATGCAGGATAACAATACTGATGTGTACTGTATTCGTTCTAACCAATTCTCAGTTTATGTTCATTCCACTTGCAAAAGTTCTTTATGGGACAATATTTTTAATCAAGACTGCACGGATGTTTTAGAGGCTACAGCTGTTATAAAAACTGGTACTTGTCCTTTCTCATTTGATAAATTGAACAATTACTTGACTTTTAACAAGTTCTGTTTGTCGTTGAGTCCTGTTGGTGCTAATTGCAAGTTTGATGTTGCTGCACGTACAAGAACCAATGAGCAGGTTGTTAGAAGTCTATATGTAATATATGAAGAAGGAGACAACATAGTGGGTGTACCGTCTGATAATAGCGGTCTGCACGATTTGTCTGTGCTACACCTAGACTCCTGTACAGATTACAATATATATGGTAGAACTGGTGTTGGTATTATTAGACGAACTAACAGTACGCTACTTAGTGGCTTATATTACACATCACTATCAGGTGATTTGTTAGGCTTTaAAAATGTTAGTGATGGTGTCATTTATTCTGTGACGCCATGTGATGTAAGCGCACAAGcGGCTGTTATTGATGGTGCCATAGTTGGAGCTATGACTTCCATTAACAGTGAACTGTTAGGTCTAACACATTGGACAACGACACCTAATTTTTATTACTACTCTATATATAATTACACAAGTGAGAGGACTCGTGACACTGCAATTGACAGTAACGATGTTGATTGTGAACCTGTCATAACCTATTCTAATATAGGTGTTTGTAAAAATGGTGCTTTGGTTTTTATTAACGTCACACATTCTGACGGAGACGTGCAACCAATTAGCACTGGTAATGTCACGATACCTACAAATTTTACCATATCTGTGCAAGTTGAATACATGCAGGTTTACACTACACCAGTATCAATAGATTGTGCAAGATACGTTTGTAATGGTAACCCTAGATGTAACAAATTGTTAACACAATATGTGTCTGCATGTCAAACTATTGAACAAGCACTTGCAATGGGTGcCAGACTTGAAAACATGGAGGTTGATTCCATGTTGTTTGTCTCGGAAAATGCCCTTAAATTGGCATCTGTTGAGGCGTTCAATAGTACAGAAAATTTAGATTCTATTTACAAAGAATGGCCTAGCATAGGTGGTTCTTGgCTAGGAGGTCTAAAAGATATACTACCGTCCCATAATAGCAAACGtAAGTATGGTTCTGCTATAGAAGATTTGCTTTTTGATAAAGTTGTAACATCTGGTTTAGGTACAGTTGATGAAGATTATAAACGTTGTACTGGTGGTTACGACATAGCAGACTTGGTGTGTGCTCAATATTACAATGGCATCATGGTTCTACCAGGTGTAGCTAATGCTGACAAGATGACTATGTACACAGCATCACTTGCAGGTGGTATAACATTAGGTGCACTTGGTGGTGGCGCCGTGGCTATACCTTTTGCAGTAGCAGTACAGGCTAGACTTAATTATGTTGCTCTACAAACTGATGTATTGAATAAAAACCAACAGATCCTGGCTAATGCCTTCAATCAAGCTATTGGTAACATTACACAGGCTTTTGGTAAGGTTAATGATGCTATACATCAAACATCACAAGGTCTTGCCACTGTTGCTAAAGCGTTGGCAAAAGTGCAAGATGTTGTCAACACACAAGGGCAAGCTTTAAGTCACCTTACAGTACAATTGCAAAATAATTTTCAAGCCATTAGTAGTTCTATTAGTGATATTTATAACAGGCTTGACGAACTGAGTGCTGATGCACAAGTTGATAGGCTGATTACAGGTAGACTTACAGCACTTAATGCATTTGTGTCTCAGACTCTAACCAGACAAGCAGAGGTTAGGGCTAGTAGACAACTTGCCAAAGACAAGGTTAATGAATGTGTTAGGTCTCAGTCTCAGAGATTCGGATTCTGTGGTAATGGTACACATTTGTTTTCACTAGCAAATGCAGCACCAAATGGCATGATTTTCTTTCATACAGTACTATTACCAACAGCTTATGAAACTGTAACAGCTTGGTCAGGTATTTGTGCTTCAGATGGCGATCGCACTTTCGGACTTGTCGTTAAAGATGTGCAGTTGACGTTGTTTCGTAATCTAGATGACAAGTTCTATTTGACCCCCAGAACTATGTATCAGCCTAGAGTTGCAACTAGTTCTGATTTTGTTCAAATTGAAGGGTGTGATGTGTTGTTTGTCAACGCGACTGTAATTGATTTGCCTAGTATTATACCTGACTATATTGACATTAATCAAACTGTTCAAGACATATTAGAAAATTACAGACCAAACTGGACTGTACCTGAATTTACACTTGATATTTTCAACGCAACCTATTTaAATCTGACTGGTGAAATTGATGACTTAGAGTTTAGGTCAGAAAAGCTACATAACACTACAGTAGAACTTGCCATTCTCATTGATAACATTAATAATACATTAGTCAATCTTGAATGGCTCAATAGAATTGAAACTTATGTAAAATGGCCGTGGTATGTGTGGCTACTAATCGGTTTAGTAGTGGTCTTCTGCATACCATTGTTACTATTTTGCTGTCTGAGTACTGGATGTTGTGGGTGCTTTGGTTGCCTTGTAAGTTGTTGCAATTCTCTTTGTAGTAGAAGACAATTTGAAAGCTACGAACCTATCGAAAAGGTTCACATCCATTAACTAAACGATTTATGGACGCCGTCAAGTCTATTGGCATCTCTGTTGACGCTGTACTTGACGAATTAGATTCCATTGCTTTTGCTGTAACACTTAAAGTTTTATTTAATTCTGGTAAATTACTTGTGTGTATAGGTTTTGGTGATACTTTTGAAGAGGCTGAACAAAAAGCTTATGCCAAATCAAAACTGGTATAAGTTACCTTTTGTAGTCAGATTACGTATCATAAATAATACAAAACCTAGAACAGCAAGCACTATAAAACGCAGACGAAGGGCTGTTATTGACTACATAAAAATCGCTATTCTCAACGCGACGCGAAAATGATTGGCGGACTGTTTCTTAACGCTCTAAGTTTTATAGTTACTAATCAACATGTGATTGCTAATAATACATCACATGTTAATACTATAGTACAACAACACCATGTTGTTAGTGCTAATATACCTATTAAAAGTTTTCATTTAGAGTTCAGCATTGCTGTGCTCTTCGTTTTATTTTTAGCTTTGTACCGTAGTACAAACTTTAAAGTGTGTGTCGGTGTCTTAATGTTTAAGATAGTATCAATGACACTTATAGGACCTATGCTCATAGCATTTGGTTACTACATAGATGGCATTGTGACAATAACTGTCTTAGCTTTAAGATTTATTTACTTAGCATACTTTTGGTATGTTAATAGTAGATTTGAATTCGTTTTATACAATACGACGACACTAATGTTTGTACACGACAGAGCTGCACCGTTTATGAGAAGTTCTCACGGCTCTATTTGTGTCACACTATACGGAGGCATAAATTACATGTTCGTGAATGATCTTACGTTGCATTTTGTAGATCCTATGCTTGGCATATCTATACGTGGCCTAGTTCATGCTGACCTAACAGTTGTTAGAGCAGTTGAACTTCTCAATGGTGATTTTATCTATATATTTTCACAGGAGGCCGTCGTAGGAGTTTACAATGCAGCTTTTTCTCAGGCGGTTATAAACGAAATTGATTTGAAAGACGAAGAAGAGCGTGTCTATGATGTTTCCTAGGGCATTTACTATCATAGACGACCATGGTATGGTTGTTAGCGTTTTCTTCTGGCTCCTGTTGATAATTATATTGATATTGTTTTCAATAGCATTGCTAAATGTTATTAAATTGTGTATGGTGTGCTGCAATTTGGGTAAGACTATTGTAGTACTACCTGCTCGCCATGCATATGATGCCTATAAGACCTTTATGCAGATTAAGGCATACAATCCCGACGAAGCATTTTTGGTTTGAACTAAACAAAATGAAGTACATTTTGTTAATACTCGCGTGCATTATTGCATGCGTTTATGGAGAACGCTACTGTGCCATGCAAAATACAGGCTCgCAGTGCATTAATGGCACAGATTCATCATGTAGCACCTGTTTTGAACGTGGTGgTCTTATTTGGCATCTGGCTAACTGGAACTTCAGCTGGTCTGTAATATTGATTGTTTTTATAACAGTGTTAaAATATGGAAGACCGCAATTCAGCTGGCTCGTTTATGGCATTAAAATGCTGATCATGTGGCTATTATGGCCTATTGTTCTAGCGCTTACGATTTTTAATGCATACTCTGAGTACCAAGTTTCCAGATATGTAATGTTCGGCTTTAGTGTTGCAGGTGCAGTTGTAACGTTTGCACTATGGATGATGTATTTTGTGAGATCTATTCAGCTGTATAGACGGACCAAATCATGGTGGTCTTTTAATCCTGAAACCAATGCGATTCTTTGTGTCAATGCATTGGGTAGAAGCTATGTACTCCCTCTTGATGGCACTCCTACAGGTGTTACTCTTACCCTACTTTCAGGAAATCTATACGCTGAAGGTTTTAAAATGGCTGGTGGTCTTACCATCGAGCATTTGCCTAAATATGTCATGATTGCTACGCCTAGTAGAACCATCGTTTACACATTAGTTGGAAAACAACTAAAGGCAACTACTGCCACTGGATGGGCTTACTATGTAAAATCTAAAGCTGGTGATTACTCAACAGAAGCACGTACTGATAATTTGAGTGAACATGAAAAATTATTACATATGGTGTAACTAAACTTCTAAATGGCCACACAGGGACAACGCGTCAACTGGGGAGATGAACCTTCCAAAAGACGTGGTCGTTCTAACTCTCGTGGTCGGAAGAATAACACTATACCTCTTTCATTCTTCAATCCCATCCAACTCGAACCAGGATCAAAATTTTGGAGCGTATGTCCGAGAGATTTTGTTCCCAAGGGAATAGGTAACAAGGATCAACAAATTGGTTATTGGAATAGACAAGAGCGTTACCGCATTGTCAAAGGTCAGCGTAAGGAACTTCCTGAGAGGTGGTTTTTCTACTTCTTAGGCACAGGACCTCAAGCTGATGCTAAATTTAAAGACAAGATTGATGGAGTCTTCTGGGTTGCAAAGGATGGTGCCATGAATAAACCAACAACACTTGGCACTCGTGGTACCAACAATGAATCCAAACCACTGAAATTTGATGGTAAGATACCACCGCAATTTCAGCTTGAAGTGAACCGATCTAGGAACAACTCAAGAAGTGGTTCTCAGTCTAGATCTGTCTCTAGAAACAGGTCTCAATCCAGAGGAAGACAACAATCCAATAATCAGAATAATGTTGAGGATACAATTGTAGCTGTGCTTCAGAAATTAGGTGTTACTGAAAAGCAAAGGTCACGTTCTAAATCTAGAGATCGTGGTGACTCTAAACCTAGAGACACAACACCTAATAATGCCAACAAACACACCTGGAAGAAGACTGCAGGTAAAGGTGATGTGACAAATTTCTATGGTGCTAGAAGTGCTTCAGCTAACTTTGGTGATAGTGATCTCGTTGCCAACGGTAACGCTGCCAAATCCTACCCTCAGATAGCTGAATGCGTTCCATCAGTGTCTAGCATGCTCTTCGGTAGTCAATGGTCTGCTGAAGATGATGGTGATCAAGTGAAAGTCACGCTCACTCATACCTATTACCTGCCAAAAGATGATGCCAAAACCAGCCAATTCCTAGAACAGATTGACGCTTACAAGCGGCCATCTCAAGTGGCTAAAGATCAGAGGCAAAGAAAATCTCGTTCTAAGTCTGCTGAGAAGAAGCCTGAGGAATTGTCTGTAACTCTTGTAGAGGCATATACGGATGTGTTTGATGACACACAGGTTGAGATGATTGATGAGGTTACGAACTAAACGCATGCTCGTTTTCCTCCATGCTGTACTTGTTACAGTTTTAATCTTACCACTAATTGGTAGAATCCAATTACTGGAAAGGTTATTACTCAGTCATCTGCTTAATCTTACAACAGTCAGTAATGTTTTAGGTGTGCCTGACAGTAGTCTGCGTGTAAATTGCTTACAGCTTTTGAAACCAGACTGTCTTGATTTTAACATCTTACACAAAGTTTTAGCAGAAACCAGATTACTAGTAGTAGTACTGCGAGTGATCTTTCTGGTTCTTCTAGGGTTTTCCTGCTATACATTGCTAGGTGCATTATTTTAACATCATGATTGTTGTACTCCTTGTGTGTGTTTTCTTGGCTAATGGACTTAAAGCTACTATTGTGCAATCTGACCCTCATGAACATCCAGTTCTTACATGGGAACTTTTGCAACATTTCATAGGAAGCACTCTCTACATTACAACACATCAGATTTTAGCATTACCGCTTGGATCGCGTGTTGAGTGTGAGAGTGTTGAAGGTTTCAATTGCACATGGCCTGGTTTTCAAAAGCCTGCACATGATCACATTGATTTCTATTTTGATCTTTCTAATCCTTTCTATTCCTTTGTAGATAATTTTTATATTGTAGGTGATGGAAATCAAAGAATTAATCTTAGATTAGTTGGTGCTGTGCCAAAACAAAAGAGATTAAATGTTGGTTGTTATATATCATTTGCTGTTGACCTTCCATTTGGAACTCAGATATACCATGACAGAGATTTTCAACACCCTGTTAGTGGTAGACATCTAGAGTGTACTCACAGAGTCTACTTTGTGAAGTATTGTCCACACAACCTGTATGGTTATTGCTTTAATGAGAAGCTGAAAGTTTATAACTTGACGCAACTCAGAAGCAAGAAGGTTTTCGACAAAATCAACCAACATCATAAAACTGAGTTATAAGGCAACCCGATGTCTAAAACTGGTCTTTCCGAGGAATTG

**References**

1. Larkin MA, Blackshields G, Brown NP, Chenna R, McGettigan PA, McWilliam H, Valentin F, Wallace IM, Wilm A, Lopez R, Thompson JD, Gibson TJ, Higgins DG. 2007. Clustal W and Clustal X version 2.0. Bioinformatics 23:2947-8.

2. Fu Y, Fu Z, Su Z, Li L, Yang Y, Tan Y, Xiang Y, Shi Y, Xie S, Sun L, Peng G. 2023. mLST8 is essential for coronavirus replication and regulates its replication through the mTORC1 pathway. mBio doi:10.1128/mbio.00899-23:e0089923.
